# Genome-Wide Identification and Characterization of Fox Genes in the Honeybee, *Apis cerana*, and Comparative Analysis with Other Bee Fox Genes

**DOI:** 10.1155/2018/5702061

**Published:** 2018-04-16

**Authors:** Hongyi Nie, Haiyang Geng, Yan Lin, Shupeng Xu, Zhiguo Li, Yazhou Zhao, Songkun Su

**Affiliations:** ^1^College of Bee Science, Fujian Agriculture and Forestry University, Fuzhou 350002, China; ^2^Institute of Apiculture, Chinese Academy of Agricultural Sciences, Beijing 100093, China

## Abstract

The forkhead box (Fox) gene family, one of the most important families of transcription factors, participates in various biological processes. However, Fox genes in Hymenoptera are still poorly known. In this study, 14 Fox genes were identified in the genome of *Apis cerana*. In addition, 16 *(Apis mellifera)*, 13 *(Apis dorsata)*, 16 *(Apis florea)*, 17 *(Bombus terrestris)*, 16 *(Bombus impatiens)*, and 18 *(Megachile rotundata)* Fox genes were identified in their genomes, respectively. Phylogenetic analyses suggest that FoxA is absent in the genome of *A. dorsata* genome. Similarly, FoxG is missing in the genomes *A. cerana* and *A. dorsata*. Temporal expression profiles obtained by quantitative real-time PCR revealed that Fox genes have distinct expression patterns in *A. cerana*, especially for three genes *ACSNU03719T0 (AcFoxN4)*, *ACSNU05765T0 (AcFoxB)*, and *ACSNU07465T0 (AcFoxL2)*, which displayed high expression at the egg stage. Tissue expression patterns showed that *FoxJ1* is significantly higher in the antennae of *A. cerana* and *A. mellifera* compared to other tissues. These results may facilitate a better understanding of the potential physiological functions of the Fox gene family in *A. cerana* and provide valuable information for a comprehensive functional analysis of the Fox gene family in Hymenopterans.

## 1. Introduction

The forkhead box (Fox) belongs to a large and diverse group of transcription factor families. It is characterized by a highly conserved forkhead (FKH) DNA-binding domain, which consists of approximately 100 residues with three *α*-helices, three *β*-sheets, and two “wing” regions that flank the third *β*-sheet [1–4]. The Fox gene family was initially identified in the embryo of *Drosophila* [5]. Over the past two decades, numerous Fox genes have been identified from a wide variety of taxa and have been classified into 23 subfamilies from FoxA to FoxS [6–8]. Increasing evidences show that Fox genes mediate a multitude of physiological functions. For instance, the FoxO family is related to longevity, metabolism, development, tumor suppression, immunity, and mediation of insulin [1, 9–12]. FoxJ1 contributes to motile cilia formation and colorectal cancer progression [13, 14]. FoxJ2 is specifically expressed in meiotic spermatocytes in adult mouse testes and controls meiosis during spermatogenesis in male mice [15]. In *Drosophila melanogaster*, loss of FoxL1 affects the salivary gland position and morphology during embryonic development [16]. FoxA functions as a pioneer factor to facilitate androgen receptor transactivation and prostate cancer growth [17]. Several Fox members (FoxJ1, FoxM1, FoxO1, and FoxO3) modulate neurogenesis in adults [18]. FoxN2/3 is crucial for the formation of the larval skeleton in sea urchin [19]. In humans, a series of studies have reported that Fox genes are associated with developmental disorders and diseases, including cancer, Parkinson's disease, autism spectrum disorder, ocular abnormalities, defects in immune regulation and function, and deficiencies in language acquisition [20].

The exploration of the functions of the Fox genes has been extensively carried out in mammals, especially in humans. After the publication of several insect genomes, Fox genes have been identified in species such as *A. mellifera* (16), *D. melanogaster* (20), *Aedes aegypti* (18), *Anopheles gambiae* (20), *Bombyx mori* (17), *Heliconius melpomene* (18), and *Danaus plexippus* (19), and their functions have been revealed at the same time [19, 21–24]. However, Fox genes in Hymenopterans remain poorly understood. Honeybees are social insects, which are important model organisms for neurobiological, developmental, and sociobiological studies [25–27]. However, Fox genes of honeybees have not been intensively investigated. Western *(A. mellifera)* and eastern *(A. cerana)* honeybees are the two most important species in the genus *Apis*. In 2008, FoxP was identified in the brain of *A. mellifera*, and its expression in the worker brain was increased after eclosion, suggesting a role for FoxP in the developing and maturation of worker brains [28]. Phylogenetic analyses identified 14 Fox genes in the genome of *A. mellifera* [29]. Similar analyses on *A. cerana* have not been conducted so far.

*A. cerana* differs in several biological traits compared with *A. mellifera*. Eastern bees exhibit efficient adaptations for collecting sporadic nectar in mountains or forests and extreme weather conditions. They also show varroa mite resistance, cooperative group-level defense, long-haul flights, and effective hygienic behaviors [30–33]. The genomic sequence of *A. cerana* was published in 2015 and has provided a wealth of information for understanding the molecular basis of social behavior and eusocial evolution [34].

In this study, we described all Fox genes of seven Hymenoptera species: *A. cerana*, *A. mellifera*, *A. dorsata*, *A. florea*, *B. terrestris*, *B. impatiens*, and *M. rotundata*. The phylogenetic analysis of these genes was performed with reference to the Fox genes of three other insect species in which these genes were already characterized, *B. mori*, *D. melanogaster*, and *Danaus plexippus* [21]. Moreover, we provide a more detailed analysis for *A. cerana*, presenting the structural, spatial, and temporal expression profiles of its Fox genes. These data will pave the way for further molecular studies on the biological traits of *A. cerana*. Moreover, given the model status of the honeybee is for biological research, the knowledge gained in our study will be valuable for further explorations of other social insects.

## 2. Materials and Methods

### 2.1. Fox Gene Identification in *A. cerana* and Other Bees

To identify the complete list of the *A. cerana* Fox genes, known Fox proteins sequences of *D. melanogaster*, *B. mori*, and *D. plexippus* were used as query sequences to search for the homologous sequences against the *A. cerana* database using a basic local alignment search tool (BLAST, v2.3.0+), with an *E* value threshold of 10^−10^. Known Fox amino acid sequences of three species were downloaded from Flybase (http://flybase.org/), SilkDB (http://silkworm.genomics.org.cn/), and MonarchBase (http://monarchbase.umassmed.edu/). Redundant sequences were removed. To eliminate false-positive proteins, the domains of the candidate sequences were predicted using the Pfam online server (http://pfam.xfam.org/) to check whether they harbor a FKH domain. Only sequences with such clear FKH domains were retained. The same procedure was applied to search Fox family members in the protein databases of *A. mellifera*, *A. dorsata*, *A. florea*, *B. terrestris*, *B. impatiens*, and *M. rotundata*. Predicted protein sequences of *A. mellifera* were downloaded from BeeBase (http://hymenopteragenome.org/beebase/). The other translated protein sequences belonging to *A. cerana*, *A. dorsata*, *A. florea*, *B. terrestris*, *B. impatiens*, and *M. rotundata* were downloaded from the National Center for Biotechnology Information (NCBI) FTP site (ftp://ftp.ncbi.nih.gov/genomes/).

The molecular weight of AcFox was predicted using ExPASY (http://www.expasy.org/) [35], and the structures were predicted with GSDS 2.0 (http://gsds.cbi.pku.edu.cn/) [36].

### 2.2. Phylogenetic Analysis

The complete protein sequences of Fox from the 10 species *(D. melanogaster*, *B. mori*, *D. plexippus*, *A. cerana*, *A. mellifera*, *A. dorsata*, *A. florea*, *B. terrestris*, *B. impatiens*, and *M. rotundata)* were initially aligned using Clustal W (V1.83) [37]. Protein sequences and accession numbers are provided in Supplemental File 1. The phylogenetic tree for Fox was constructed using the neighbor-joining (NJ) method in the program MEGA7.0. The Poisson model, pairwise deletion of gaps, and uniform rates were set in the NJ tree reconstruction. The accuracy of the tree topology was tested by bootstrap analysis with 1000 resampling replicates.

### 2.3. Multiple Sequence Alignment

A multiple sequence alignment was constructed with the Clustal W multiple alignment program in BioEdit 7.05. Then, alignment was artificially edited and visualized using GeneDoc software. Finally, the secondary structures of the FKH domain were predicted with SWISS-MODEL (https://www.swissmodel.expasy.org/) [38].

### 2.4. Bee Rearing and Sample Collection

Bees of *A. cerana* and *A. mellifera* were bred at the College of Bee Science, Fujian Agriculture and Forestry University, during the autumn of 2016. For *A. cerana*, eggs at different developmental stages were collected directly from combs and then mixed as egg specimens. For collection of larvae and pupae, we used a method previously described [39], which allows to determine the precise time of oviposition. Briefly, queens of *A. cerana* were caged at the comb using an excluder cage (7.2 × 5.1 × 2.2 cm) for 6 h, which made them lay eggs in the confinement region. The queen was then removed to another confinement cage in the hive while the eggs developed within the laying area, which remained enclosed. In this way, several areas for larvae and pupae collection were established within the hive, for which the precise timing of egg laying and development was known. For temporal expression analysis, brood combs of *A. cerana* were maintained in their original colonies, and samples were collected at 24 h intervals from hatching to imago. Newly emerged workers (NEW), nurses, and foragers were collected as previous methods [40, 41]. NEW were obtained from capped brood frames freed from any other bees that were placed in an incubator for 3-4 h. This period was sufficient for the emergency of the new bees. Nurses were workers entering the cells containing larvae. Foragers were workers returning to the colony with pollen loads on their hind legs. After specimen collection, tissue samples of the three bee categories (antennae, wings, midgut, head, thorax, abdomen, front leg, middle leg, and hind leg) were dissected on ice. The number of bees collected within each category/tissue combination was sufficient to ensure the analyses on Fox expression.

For *A. mellifera*, adult workers were caught randomly at the hive entrance and dissected to obtain samples of the nine types of tissue described above. The antennae and wings of about 50 worker bees were dissected, and the other seven types of tissues (midgut, head, thorax, abdomen, front leg, middle leg, and hind leg) from 10 worker bees were separated, respectively. All samples were snap frozen in liquid nitrogen and stored at −80°C prior to RNA isolation.

### 2.5. Expression Patterns Based on qRT-PCR

The total RNA of various samples was isolated using TransZol UP (TransGen Biotech) according to manufacturer's instructions. RNA was detected in agarose gel, and the concentration and purity was measured using the Nanodrop 2000 spectrophotometer (Thermo Fisher Scientific, Waltham, MA, USA). All quality results of extracted RNA are listed in Figure S1. RNA has three bands in electrophoresis, and the OD_260/280_ ratio was in the range of 1.9~2.1. Then, the RNA was purified with absolute alcohol and treated with DNaseI. Two micrograms of the total RNA was used to synthesize cDNA with PrimeScript RT reagent kits (RR037A, Takara). qRT-PCR was performed using an ABI7500 real-time PCR system (Applied Biosystems) as previously described [42]. The relative expression of each gene was calculated with 2^−△△Ct^ [43]. The *A. cerana actin-related protein* 1 *(ACSNU01044T0/HM640276)* was used as an internal control for normalization of sample loading due to its previous application as referece genes in *Apis cerana* [44]. The primers are listed in Table S1. Each sample was triplicated, and all data were presented as the mean ± standard deviation.

Statistical analyses were performed using GraphPad Prism 5.0. Student's *t*-test was used to evaluate statistical significance (^∗∗∗^ *p* < 0.001).

## 3. Results

### 3.1. Identification of Fox Genes

A BLAST (v2.3.0+) search against the genome of *A. cerana*, *A. mellifera*, *A. dorsata*, *A. florea*, *B. terrestris*, *B. impatiens*, and *M. rotundata* was performed using known Fox protein sequences of *B. mori* (17 Fox proteins), *D. melanogaster* (20 Fox proteins), and *D. plexippus* (19 Fox proteins) to identify all Fox homologs. Fourteen Fox genes were identified in the *A. cerana* genome (Table 1). The molecular weight of each *A. cerana* Fox protein was predicted by an online tool termed Compute pI/Mw in the ExPASy website (http://web.expasy.org/compute_pi/). Fox genes were named according to the nomenclature proposed by Song et al. and Kaestner et al. [21, 45]. For *A. mellifera*, *A. dorsata*, *A. florea*, *B. terrestris*, *B. impatiens*, and *M. rotundata*, 16, 13, 16, 17, 16, and 18 Fox genes were, respectively, identified in their genomes (Table S2).

### 3.2. Phylogenetic Analysis of the Fox Genes

**Figure 1:**
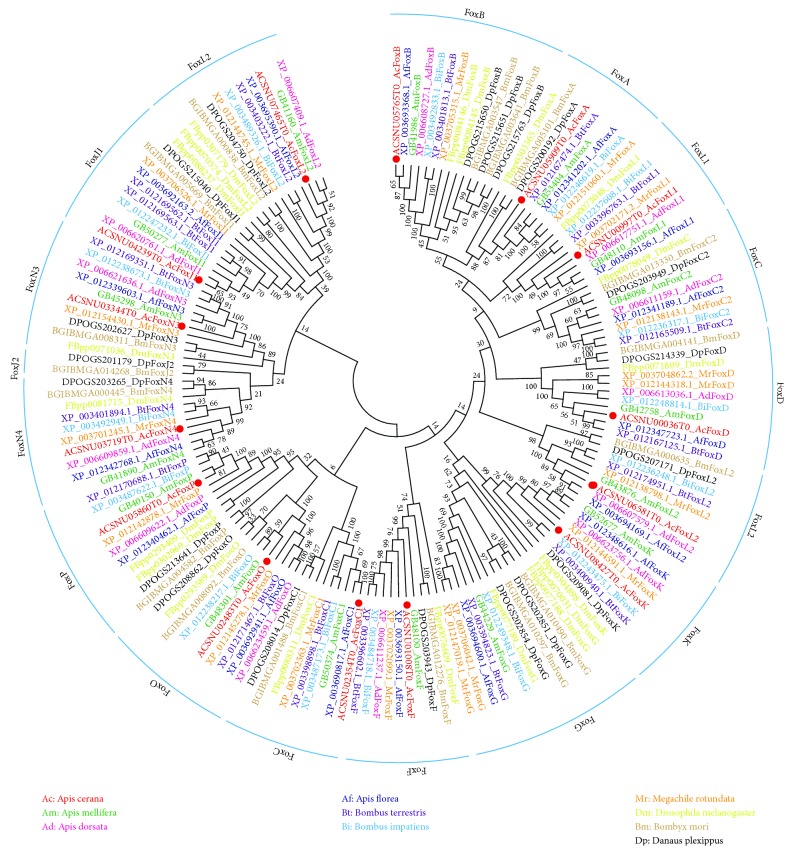
Phylogenetic tree of Fox including the 7 bee species considered in this work *(A. cerana*, *A. mellifera*, *A. dorsata*, *A. florea*, *B. terrestris*, *B. impatiens*, and *M. rotundata)* and the 3 other insect species used as reference *(B. mori*, *D. melanogaster*, and *D. plexippus)*. Colors represent different species. Red dots indicate *A. cerana* Fox genes. Protein sequences and accession numbers are provided in Supplemental File 1.

To further elucidate the phylogenetic relationship among insect Fox proteins, the Fox genes of all 7 bee species were compared with the known insect Fox proteins of *B. mori*, *D. melanogaster*, and *D. plexippus* employing a phylogenetic approach. A total of 166 insect Fox sequences were used to construct the phylogenetic tree (Figure 1). All amino acid sequences are listed in Supplemental File 1. The topologies of the subclades were similar to those reported in a previous study on Fox proteins in other insect species including *B. mori* and *D. melanogaster* [21, 23]. The phylogenetic analysis showed that the AcFox genes could be divided into 13 subfamilies (Figure 1). Notably, the two members of the FoxL2 subfamilies, which are found in 7 bee species, were not assigned to the same cluster, suggesting great differences in the sequences of different members within the FoxL2 subfamilies. Interestingly, we found no FoxJ2 subfamily in the 7 bee species investigated, whereas it was present in *B. mori* and *D. plexippus*; FoxG was absent in *A. cerana* and *A. dorsata*, but was present in all other insects investigated. The FoxA subfamily was specifically absent in *A. dorsata*, but it was present in all the other species investigated (Figure 1 and Table S2).

### 3.3. Multiple Sequence Alignment and Structure Analysis of the *A. cerana* Fox Proteins

All Fox proteins contain a highly conserved FHK domain with approximately 100 residues (DNA-binding domain). Most of this domain consists of three *α*-helices, three *β*-sheets, and two wing regions flanking the *β*-sheets [3, 4]. Multiple sequence alignment was performed with the FKH domains to assess the sequence conservation of AcFoxs, (Figure S3). Six of the proteins (ACSNU00036T0, ACSNU05765T0, ACSNU05909T0, ACSNU06581T0, ACSNU02354T0, and ACSNU01008T0) possessed four *α*-helices and three *β*-sheets. Four proteins (ACSNU03344T0, ACSNU04239T0, ACSNU07465T0, and ACSNU08427T0) consisted of three *α*-helices and three *β*-sheets. ACSNU05860T0 and ACSNU02483T0 harbored four *α*-helices and one *β*-sheet. ACSNU00997T0 comprised four *α*-helices and two *β*-sheets. ACSNU03719 had two *α*-helices and one *β*-sheet. Although these Fox proteins differed in the number of *α*-helices and *β*-sheets, they all exhibited the canonical FKH domain.

**Figure 2:**
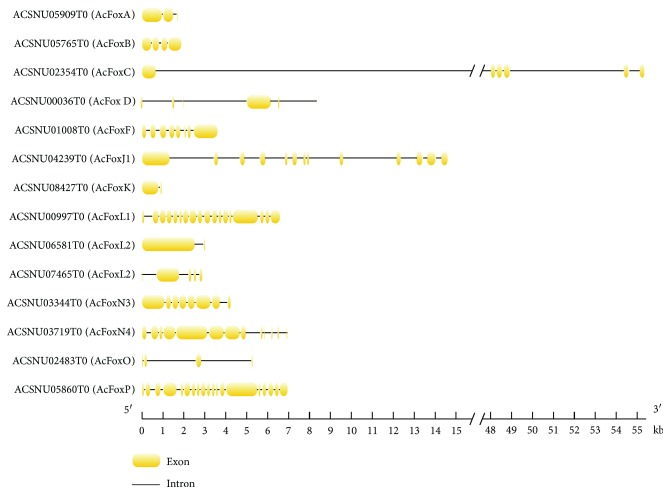
Structures of Fox genes in *A. cerana*. The putative structures of the AcFox genes consist of different numbers of exons and introns.

**Figure 3:**
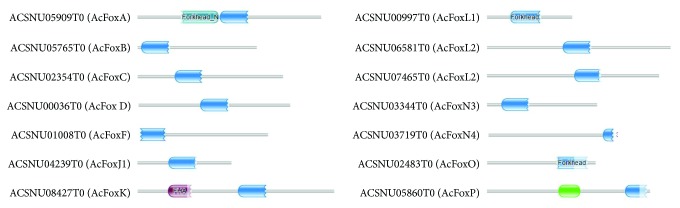
Domains of the Fox proteins in *A. cerana*. Conserved domains of the AcFox protein: dark cyan, forkhead N-terminal region; blue, forkhead domain; purple, FHA domain; green, FOXP coiled-coil domain.

The structures of the 14 AcFox genes showed a high degree of complexity with exon numbers ranging from two to nineteen (Figure 2). All the AcFox proteins contained only one FKH domain, except for the AcFoxA, AcFoxK, and AcFoxP proteins, which contained an extra N-terminal FKH region, FHA domain, and FOXP coiled-coil domain, respectively (Figure 3). These genes might have additional functions relative to other AcFox proteins.

### 3.4. Spatial and Temporal Expression Profiles of the *A. cerana* Genes

**Figure 4:**
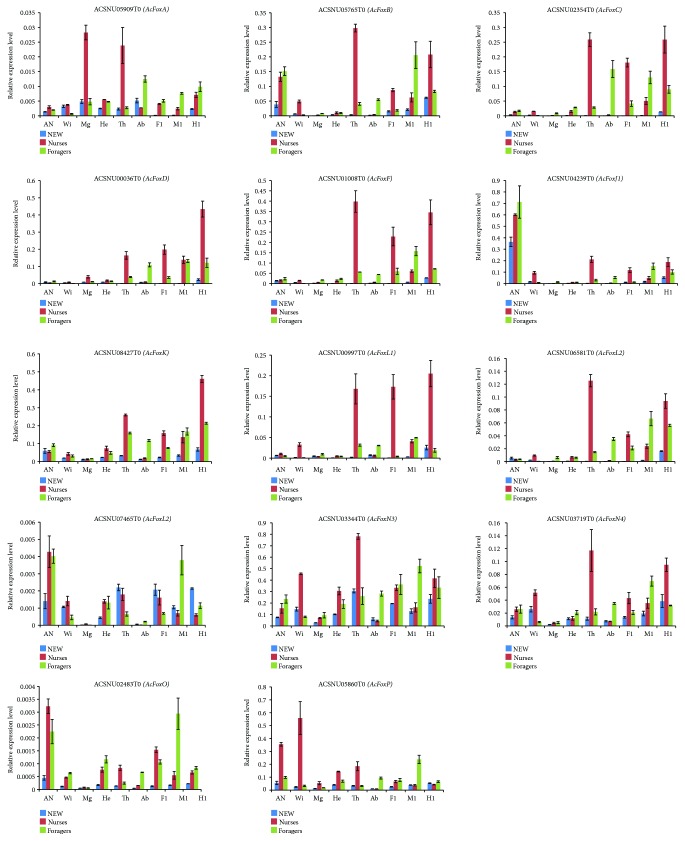
The tissue expression profiles of *A. cerana* Fox family genes in the development of newly emerged workers (NEW), nurses, and foragers. The cDNA templates were derived from the antennae (An), wings (Wi), midgut (Mg), head (He), thorax (Th), abdomen (Ab), front legs (Fl), middle legs (Ml), and hind legs (Hl).

Expression profiles of *A. cerana* Fox genes in the nine tissue types collected for the three bee categories defined (NEW, nurses, and foragers) were obtained using quantitative real-time PCR (qRT-PCR) (Figure 4). Gene expression patterns differed among tissues. Interestingly, *ACSNU04239T0 (FoxJ1)* was significantly more expressed in the antennae of all three categories of *A. cerana*. All genes, except for *ACSNU07465T0*, were more expressed in the thorax of nurses than in that of NEW and foragers. Of the 14 genes, 10 exhibited higher expression in the hind legs of nurses than in those of NEW and foragers. In nurses, the expression level of two genes *(ACSNU00036T0* and *ACSNU08427T0)* was twice as higher in the hind legs as in the middle and the front legs; thus, they might have a role in the formation of the bee's corbiculae. Finally, three genes *(ACSNU07465T0*, *ACSNU02483T0*, and *ACSNU05860T0)* were more expressed in the middle legs of foragers compared with their front and hind legs, which may suggest a role in pollen unloading. *ASCNU05909T0* was highly expressed in the midgut and thorax of nurses, but poorly expressed in other tissues of NEW, nurses, and foragers. In addition, *ACSNU05860T0* was more expressed in the wings of nurses and less expressed in other tissues of the three categories. In honeybees, workers exhibit age-related division of labor. Young bees, such as nursing workers, are engaged in brood care, whereas the oldest bees (foragers) are responsible for collecting nectar and pollen outside the hive [46, 47]. In the present study, the high expression of most FOX genes in the tissues of nurses implied that they might have evolved distinct functions for the behaviors of nurses.

**Figure 5:**
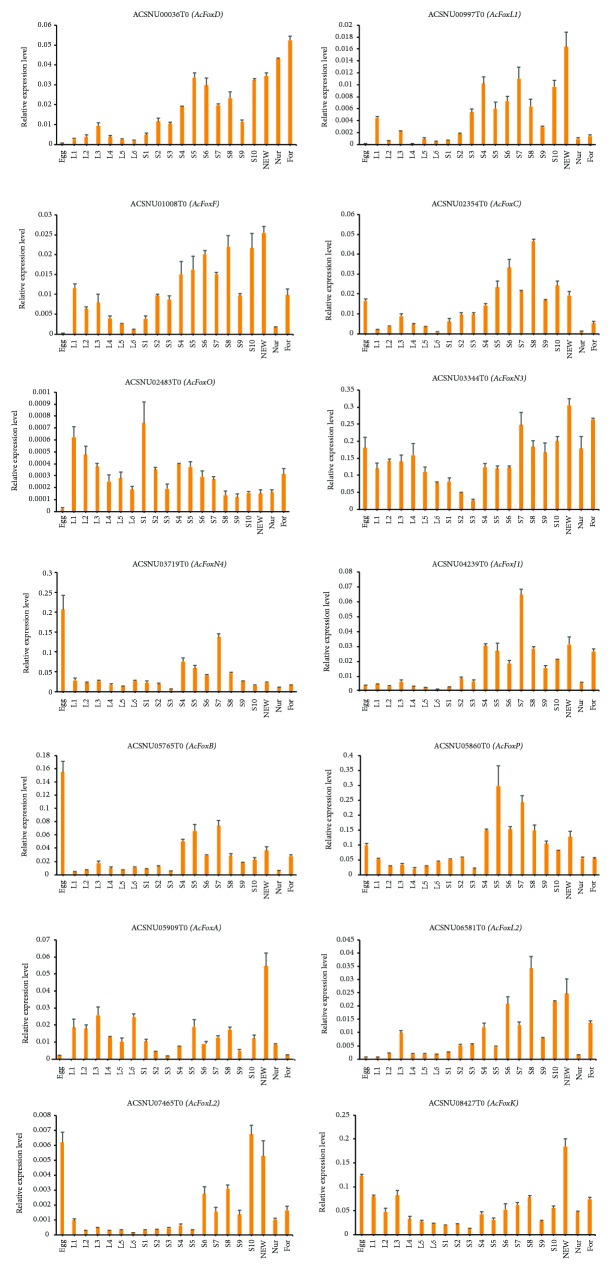
Analysis of the temporal expression pattern of 14 Fox genes in *A. cerana*. Egg: mix eggs from day 1 to 3; L1–L6: day of larval stage, from day 1 to day 6; S1–S10: day of pupal stage after cell sealing, from day 1 to day 10; NEW: newly emerged workers; Nur: nurses; For: foragers.

The temporal expression of all Fox genes was analyzed along with 20 development stages, from egg hatching to adult stage, including NEW, nurses, and foragers (Figure 5). Three genes *(ACSNU03719T0*, *ACSNU05765T0*, and *ACSNU07465T0)* were highly expressed at egg stage, suggesting that they might play a pivotal role in embryonic development. The expression of two other genes *(ACSNU01008T0* and *ACSNU02483T0)* was very weak at egg stage but increased dramatically on day 1 of larval stage and then gradually decreased from day 2 to day 6. This suggests that they might be crucial for larval growth of *A. cerana*.

### 3.5. High Levels of *FoxJ1* Expression in the Antennae of *A. mellifera* Antennae

**Figure 6:**
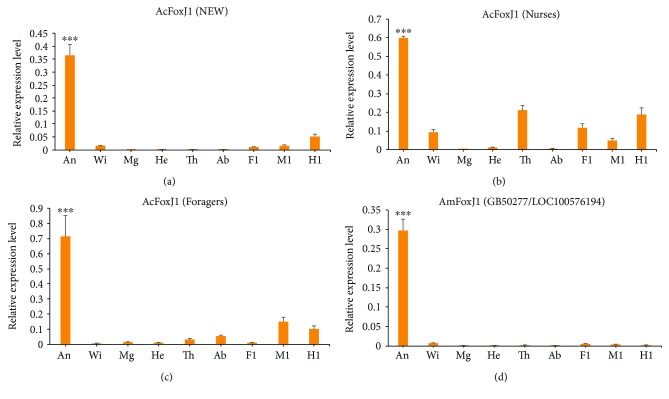
Expression patterns of *FoxJ1* in *A. cerana* and *A. mellifera*. (a, b, and c) The tissue expression profiles of *A. cerana* FoxJ1 from newly emerged workers (NEW), nurses, and foragers, respectively. (d) The tissue expression profiles of FoxJ1 in adult *A. mellifera*. ^∗∗∗^ *p* < 0.001.

*FoxJ1* was highly expressed in the antennae of all three categories of *A. cerana* (NEW, nurses, and foragers). By contrast, it was only slightly or not expressed in all other tissues. To determine whether *FoxJ1* was also highly expressed in the antennae of *A. mellifera*, the expression profile of *FoxJ1* (GB50277/LOC100576194), the homologous gene of AcFoxJ1 of *A. cerana*, was examined by qRT-PCR in the same nine different tissue types as in *A. cerana* (Figure 6). The results indicate that *FoxJ1* is also highly expressed in the antennae of adult *A. mellifera*, which is consistent with the profiles of AcFoxJ1 found in *A. cerana*.

## 4. Discussion

In the present study, our goal was to characterize the Fox genes of *A. cerana* and to perform a similar analysis in other 6 bee species. In this way, we aimed at achieving the first systematic comparison of Fox genes in Hymenoptera. We found 14 Fox genes in the genome of *A. cerana* and 16, 13, 16, 17, 16, and 18 Fox genes in the genomes of *A. mellifera*, *A. dorsata*, *A. florea*, *Bombus terrestris*, *B. impatiens*, and *M. rotundata*, respectively. Temporal expression profiles obtained by quantitative real-time PCR revealed that Fox genes have distinct expression patterns in *A. cerana*, especially for three genes *(ACSNU03719T0*, *ACSNU05765T0*, and *ACSNU07465T0)*, which displayed high expression at the egg stage. Tissue expression patterns showed that *FoxJ1* is highly expressed in the antennae of *A. cerana* and *A. mellifera* compared to other tissues.

It was reported that Fox genes of *M. musculus* and *Homo sapiens* could be classified into 23 subfamilies designed as FoxA to FoxS [21]. Nevertheless, the subfamilies FoxE, FoxH, FoxI, FoxQ, FoxR, and FoxS were completely absent from all seven species of Hymenoptera, two species of Lepidoptera *(B. mori* and *D. plexippus)*, and one species of Diptera *(D. melanogaster)* (Tables S2). Thus, the functions mediated by these genes were either inessential or they were compensated by other proteins in these species. FoxG disappeared from the *A. cerana* and *A. dorsata* genomes, while FoxA was only absent in the genome of *A. dorsata*. Therefore, FoxA and FoxG might be potential molecular markers for distinguishing *A. dorsata* and/or *A. cerana* from other bee species.

Previous studies showed that FoxJ1 mediates multiple physiological processes, especially ciliogenesis, embryonic development, spontaneous autoimmunity inhibition, and malignancy [13, 14, 48–52]. FoxJ1 was mostly reported in mammals, and its functions in insects are virtually unknown. In this study, *FoxJ1* was more expressed in the antennae of adult workers of *A. cerana* and *A. mellifera*, and the temporal expression pattern showed that *AcFoxJ1 (ACSNU04239T0)* was highly expressed from day 4 after cell sealing to NEW stage, which was consistent with the development of antennae. Meanwhile, we found that it was also expressed at the nurse and forager stages, and a higher level of expression was detected in foragers of *A. cerana*. In the honeybee, as in other insects, the antennae are the principal olfactory organs and are covered by sensilla that host olfactory receptors detecting chemical signals in the environment. In this study, *FoxJ1* was highly expressed in the antennae of *A. cerana* of NEW, nurses, and foragers, and high expression was observed from day 4 after cell sealing, followed by a continuous expression in the nurses and foragers (Figures 5 and 6). Tissue-specific genes may be relevant for the specific physiological functions of the corresponding tissues. Therefore, FoxJ1 might be involved either in the development of antennae or in the detection of specific volatiles at a certain stage. FoxJ1 was discovered in all insect genomes investigated so far, including Hymenoptera, Lepidoptera, and Diptera (Table S2). A previous study [53] showed that *AmFoxJ1 (LOC100576194)*, the homologous gene of *AcFoxJ1*, has a high expression with RPKM (reads per kilobase per million mapped reads) values of 149.2, 129.6, and 111.4 at the antennae of NEW, nurses, and foragers, respectively. Because FoxJ1 was abundantly expressed in the antennae of *A. cerana* and *A. mellifera*, it could exhibit a similar trend in the antennae of other insects. If this were the case, FoxJ1 would be a promising target gene for the study of insect olfactory systems. Additional work is necessary to investigate this topic.

In 2010, 14 Fox genes were identified in the *A. mellifera* genome [29]. All of them, except for FoxQ2, were also identified in *A. cerana* in the present study. Our results showed that FoxQ is absent in seven species of Hymenoptera, one species of Diptera, and two species of Lepidoptera. Yet, it is present in *M. musculus* and *H. sapiens* (Table S2). Only one member of both FoxL2 and FoxC subfamilies was discovered in previous research on *A. mellifera*, but we identified two members under each subfamily both in *A. mellifera* and *A. cerana*. Hence, we discovered two novel Fox genes belonging to these two subfamilies. More importantly, we identified the FoxJ1 gene in *A. mellifera*, which was present in all species investigated. The difference with previous study [29] may be due to the updating of the *A. mellifera* genome annotation from releases 102 to 103.

## 5. Conclusion

In this study, we identified several candidate Fox genes for further functional studies in the honeybee as well as in other insect species. For instance, FoxJ1 with its high level of expression in the antennae constitutes an attractive target for future research addressing its role in insect olfaction. Experiments combining RNAi-based knockdown of this gene are feasible in the honeybee. The coupling of such an approach with behavioral experiments studying olfactory learning and discrimination could reveal the role of this gene in this behavioral context. Similar studies could be conceived for other Fox genes not only in *A. mellifera* and *A. cerana* but also in other insect species, thus enabling an integrative understanding of Fox genes in several aspects of insect biology.

## Figures and Tables

**Table 1:** Summary of Fox genes in the *Apis cerana.*

Subject ID	Fox subfamily	Scaffold and interval	Exon number	Length (bp)	Predicted molecular weight (Da)
ACSNU05909T0	AcFoxA	Scaffold_0060(+): 536,171–537,866	3	2439	89,108.77
ACSNU05765T0	AcFoxB	Scaffold_0056(+): 967,977–969,846	4	1098	40,956.31
ACSNU02354T0	AcFoxC	Scaffold_0013(+): 2,876,905–2,932,333	6	1335	44,159.55
ACSNU00036T0	AcFoxD	Scaffold_0001(+): 1,036,746–1,045,196	6	1395	51,329.14
ACSNU01008T0	AcFoxF	Scaffold_0005(+): 464,006–467,605	8	1173	42,614.24
ACSNU04239T0	AcFoxJ1	Scaffold_0034(+): 1,044,883–1,059,544	13	873	32,344.77
ACSNU08427T0	AcFoxK	Scaffold_0135(+): 374,918–375,848	2	1803	65,700.29
ACSNU00997T0	AcFoxL1	Scaffold_0005(+): 387,351–393,959	18	798	30,899.18
ACSNU06581T0	AcFoxL2	Scaffold_0075(+): 438,361–441,354	2	1692	61,603.24
ACSNU07465T0	AcFoxL2	Scaffold_0100(−): 552,954–555,827	5	1602	59,830.75
ACSNU03344T0	AcFoxN3	Scaffold_0024(−): 313,506–317,738	8	1026	38,832.03
ACSNU03719T0	AcFoxN4	Scaffold_0028(+): 112,830–119,805	14	1152	43,596.57
ACSNU02483T0	AcFoxO	Scaffold_0015(−): 81,499–86,777	4	1005	37,405.76
ACSNU05860T0	AcFoxP	Scaffold_0059(−): 145,652–152,625	19	1512	56,200.78

## Abbreviations

Fox: Forkhead box
FKH: Forkhead
qRT-PCR: Quantitative real-time PCR
NEW: Newly emerged workers.

## Supplementary Materials

Supplementary Materials Figure S1: RNA electrophoresis photo of different developmental stages and different tissues in *A. cerana*. (A) RNA electrophoresis photo of different developmental stages in *A. cerana*. Egg: mix eggs from day 1 to day 3; L1–L6: day of larval from day 1 to day 6; S1–S10: day of individuals after sealing from day 1 to day 10; NEW: newly emerged workers; Nur: nurses; For: foragers. (B) RNA electrophoresis photo of different tissues on the development of newly emerged workers (NEW), nurses, and foragers. The different tissues are as follows: antennae (An), wing (Wi), midgut (Mg), head (He), thorax (Th), abdomen (Ab), front leg (Fl), middle leg (Ml), and hind leg (Hl). Figure S2: qRT-PCR melting curve of 14 primers using different templates of cDNA. (A) Melting curve of 14 primers using templates of 9 tissues of NEW. These genes are ACSNU00036T0, ACSNU00997T0, ACSNU01008T0, ACSNU02354T0, ACSNU02483T0, ACSNU03344T0, ACSNU03719T0, ACSNU04239T0, ACSNU05765T0, ACSNU05860T0, ACSNU05909T0, ACSNU06581T0, ACSNU07465T0, and ACSNU08427T0, respectively. Each primer was detected using nine different tissues, including antennae, wing, midgut, head, thorax, abdomen, front leg, middle leg, and hind leg. (B) Melting curve of 14 primers using templates of 9 tissues of nurses. Each primer was detected using nine different tissues. (C) Melting curve of 14 primers using templates of 9 tissues of foragers. Each primer was detected using nine different tissues. (D) Melting curve of 14 primers using templates of 20 different developmental stages. Figure S3: protein sequence alignment of *A. cerana* forkhead domains and their secondary structure. (A) The alignment of the amino acid sequences of *A. cerana* forkhead domains. (B) The secondary structure of *A. cerana* forkhead domains. The secondary structural elements are indicated with black cylinders and arrows representing *α*-helices and *β*-strands, respectively. Table S1: list of primer pairs used in qRT-PCR. Table S2: distribution of the members of the Fox gene subfamilies in *A. cerana* and other species. Supplemental File 1: accession numbers and protein sequences of Fox members in the phylogenetic analysis.
