# Supplementary material for: Genome-Wide Identification and Characterization of Fox Genes in the Honeybee, Apis cerana, and Comparative Analysis with Other Bee Fox Genes
Source: Int J Genomics. 2018 Apr 16;2018:5702061. doi: 10.1155/2018/5702061 (PMC5926511; doi:10.1155/2018/5702061)
Supplement: Supplementary Materials — Figure S1: RNA electrophoresis photo of different developmental stages and different tissues in A. cerana. (A) RNA electrophoresis photo of different developmental stages in A. cerana. Egg: mix eggs from day 1 to day 3; L1–L6: day of larval from day 1 to day 6; S1–S10: day of individuals after sealing from day 1 to day 10; NEW: newly emerged workers; Nur: nurses; For: foragers. (B) RNA electrophoresis photo of different tissues on the development of newly emerged workers (NEW), nurses, and foragers. The different tissues are as follows: antennae (An), wing (Wi), midgut (Mg), head (He), thorax (Th), abdomen (Ab), front leg (Fl), middle leg (Ml), and hind leg (Hl). Figure S2: qRT-PCR melting curve of 14 primers using different templates of cDNA. (A) Melting curve of 14 primers using templates of 9 tissues of NEW. These genes are ACSNU00036T0, ACSNU00997T0, ACSNU01008T0, ACSNU02354T0, ACSNU02483T0, ACSNU03344T0, ACSNU03719T0, ACSNU04239T0, ACSNU05765T0, ACSNU05860T0, ACSNU05909T0, ACSNU06581T0, ACSNU07465T0, and ACSNU08427T0, respectively. Each primer was detected using nine different tissues, including antennae, wing, midgut, head, thorax, abdomen, front leg, middle leg, and hind leg. (B) Melting curve of 14 primers using templates of 9 tissues of nurses. Each primer was detected using nine different tissues. (C) Melting curve of 14 primers using templates of 9 tissues of foragers. Each primer was detected using nine different tissues. (D) Melting curve of 14 primers using templates of 20 different developmental stages. Figure S3: protein sequence alignment of A. cerana forkhead domains and their secondary structure. (A) The alignment of the amino acid sequences of A. cerana forkhead domains. (B) The secondary structure of A. cerana forkhead domains. The secondary structural elements are indicated with black cylinders and arrows representing α-helices and β-strands, respectively. Table S1: list of primer pairs used in qRT-PCR. Table S2: distribution of the members of [file 5702061.f1.doc]

**Genome-wide identification and characterization of Fox genes in the honeybee, *Apis cerana* and comparative analysis with other bees Fox genes**

Hongyi Nie^1, #^, Haiyang Geng ^1, #^, Yan Lin^1^, Shupeng Xu ^1^, Zhiguo Li^1^, Yazhou Zhao^1, 2^, Songkun Su^1,*^

^1^College of Bee Science, Fujian Agriculture and Forestry University, Fuzhou 350002, China;

hnhynie@126.com (H. N.); haiyang.fafu@qq.com (H. G.); 331451781@qq.com (Y. L.); 460255556@qq.com (S. X.); 569670606@qq.com (Z. L.);

^2^Institute of Apiculture, Chinese Academy of Agricultural Sciences, Beijing 100093, China;

zhaoyazhou@caas.cn

^#^ These authors contributed equally to this work.

* To whom correspondence should be addressed: Songkun Su, College of Bee Science, Fujian Agriculture and Forestry University, Fuzhou 350002, China, Tel.: +86-0591-83739448, E-mail: susongkun@zju.edu.cn

**Supplementary information, Figure S1**


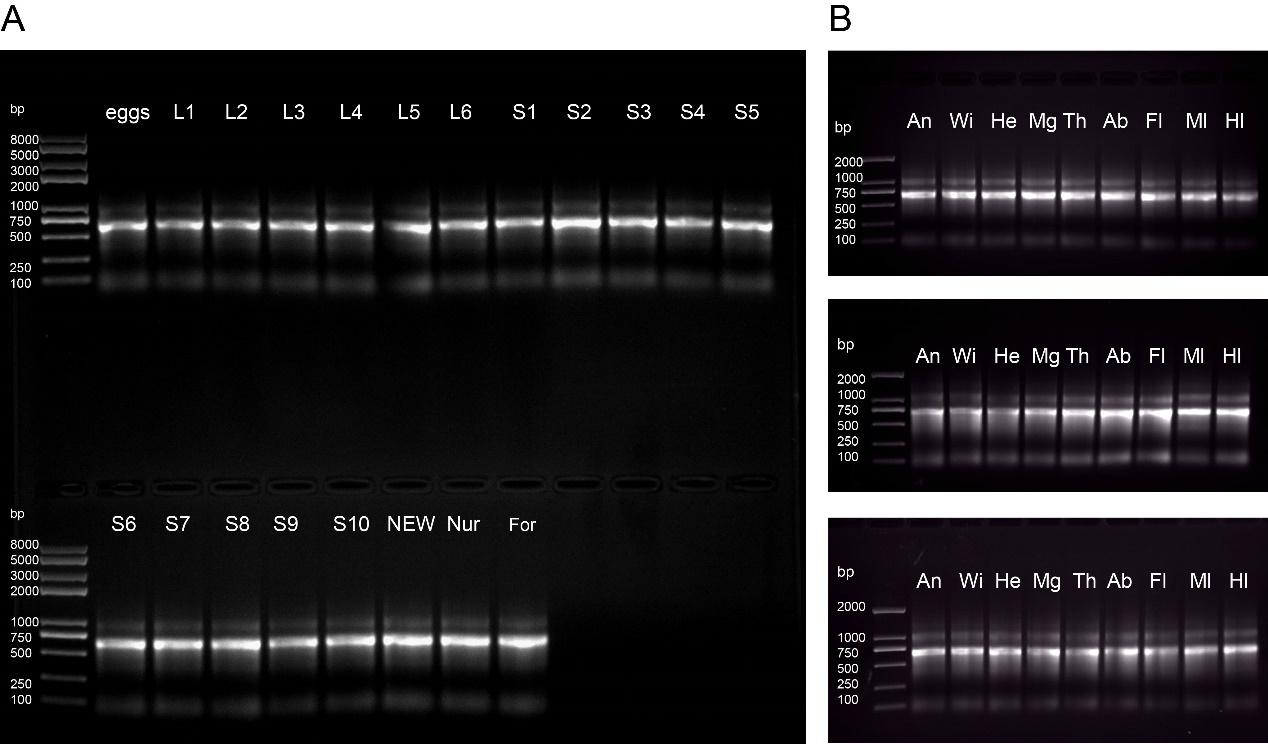


**Figure S1** RNA electrophoresis photo of different developmental stages and different tissues in *A. cerana*. (A) RNA electrophoresis photo of different developmental stages in *A. cerana*. Egg: Mix eggs from day 1 to day3, L1-L6: day of larval from day 1 to day 6, S1-S10: day of individuals after sealing from day 1 to day 10, NEW: New emerged workers, Nur: Nurses, For: Foragers. (B) RNA electrophoresis photo of different tissues on the development of newly emerged worker (NEW), nurses and foragers. The different tissues are as follows. antennae (An), wing (Wi), midgut (Mg), head (He), thorax (Th), abdomen (Ab), front leg (Fl), middle leg (Ml) and hind leg (Hl).

**Supplementary information, Figure S2**


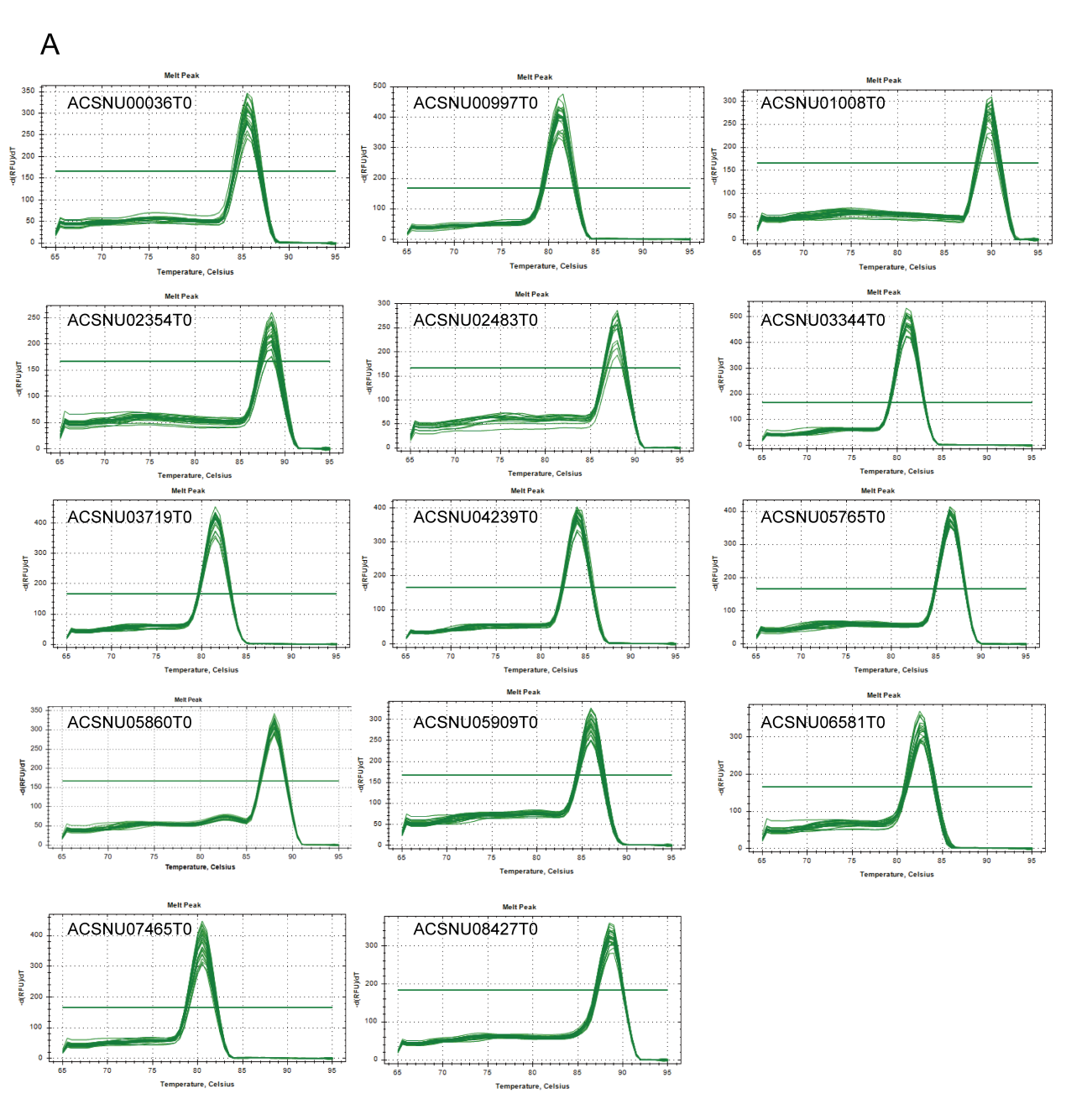


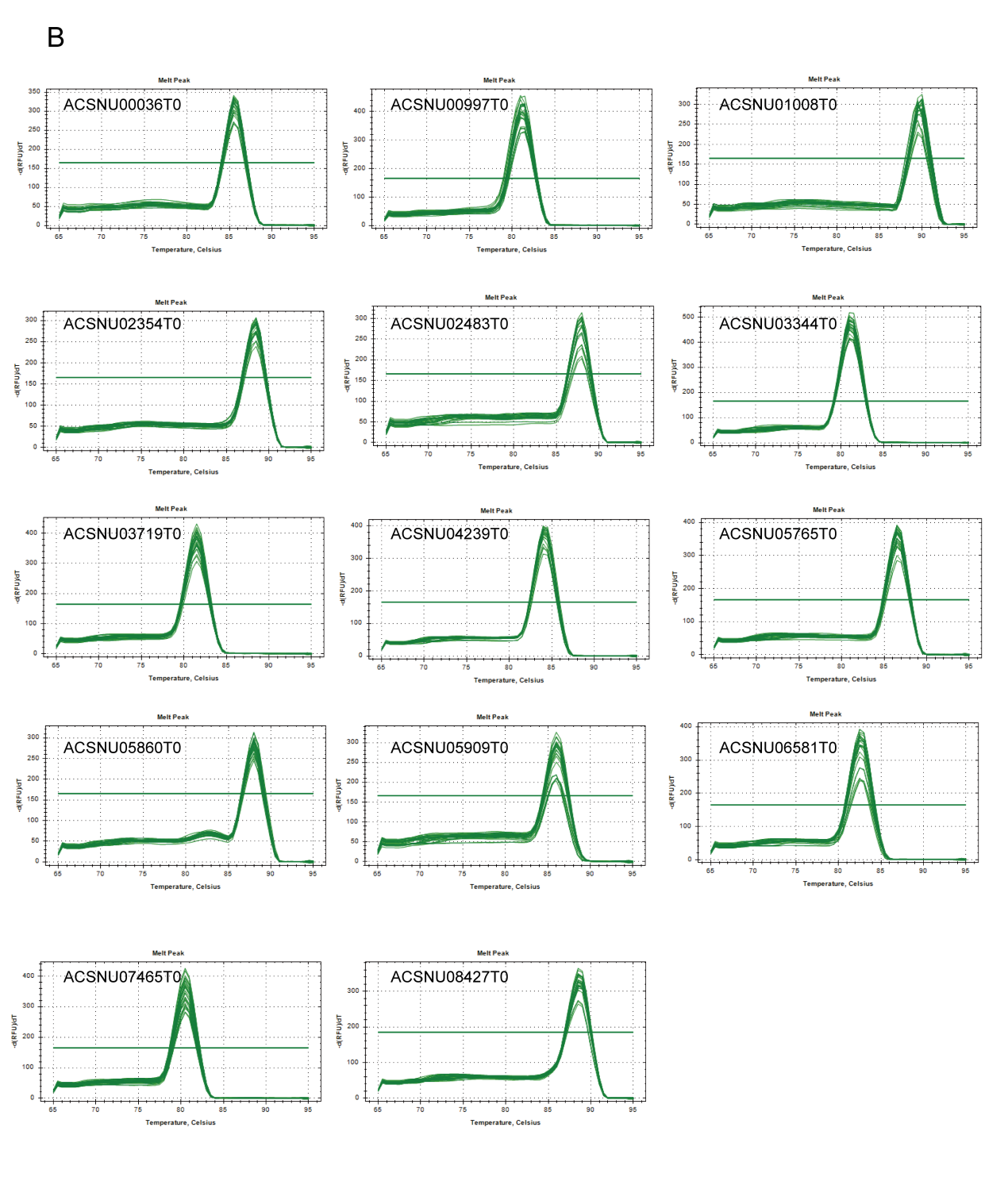

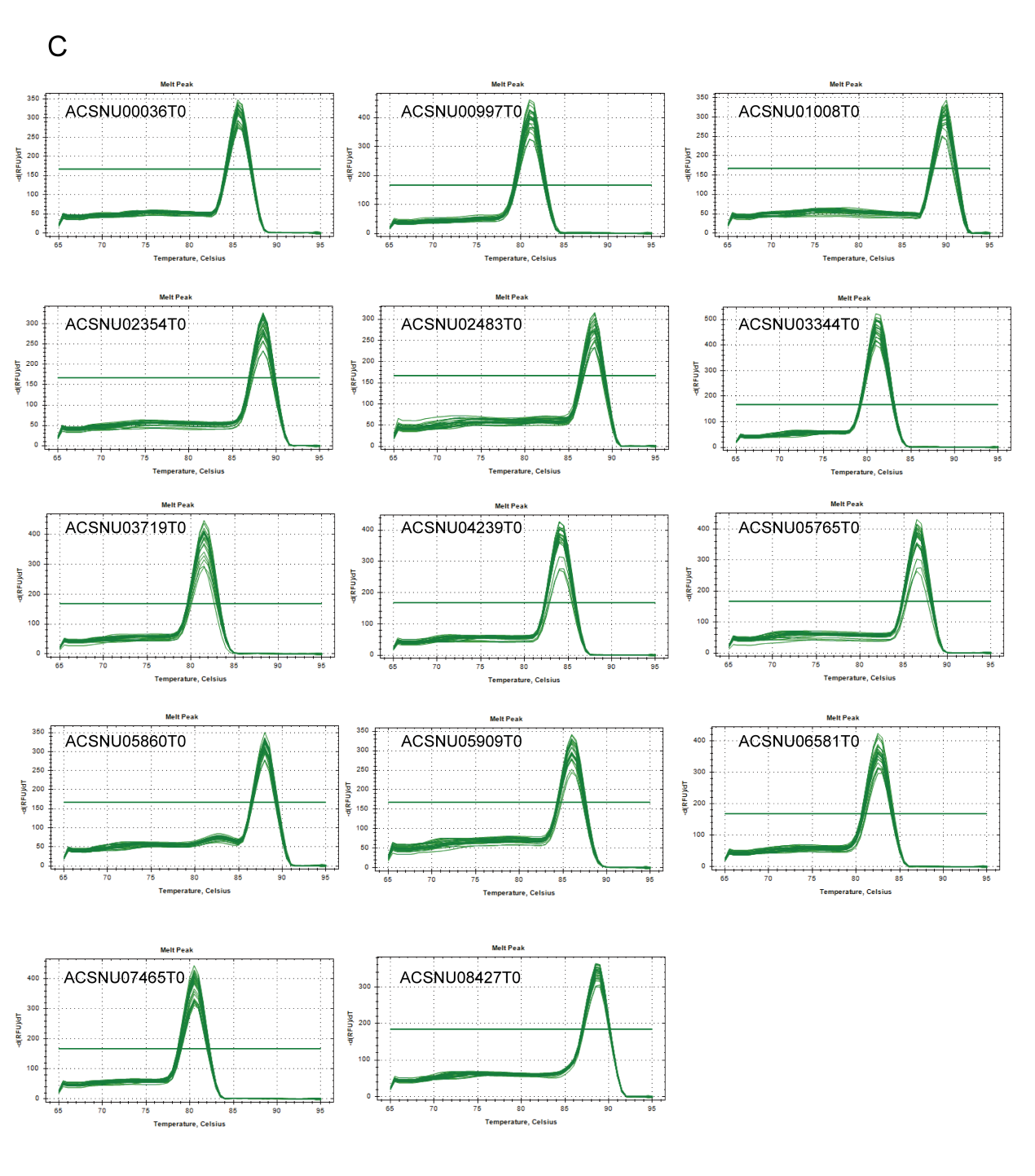

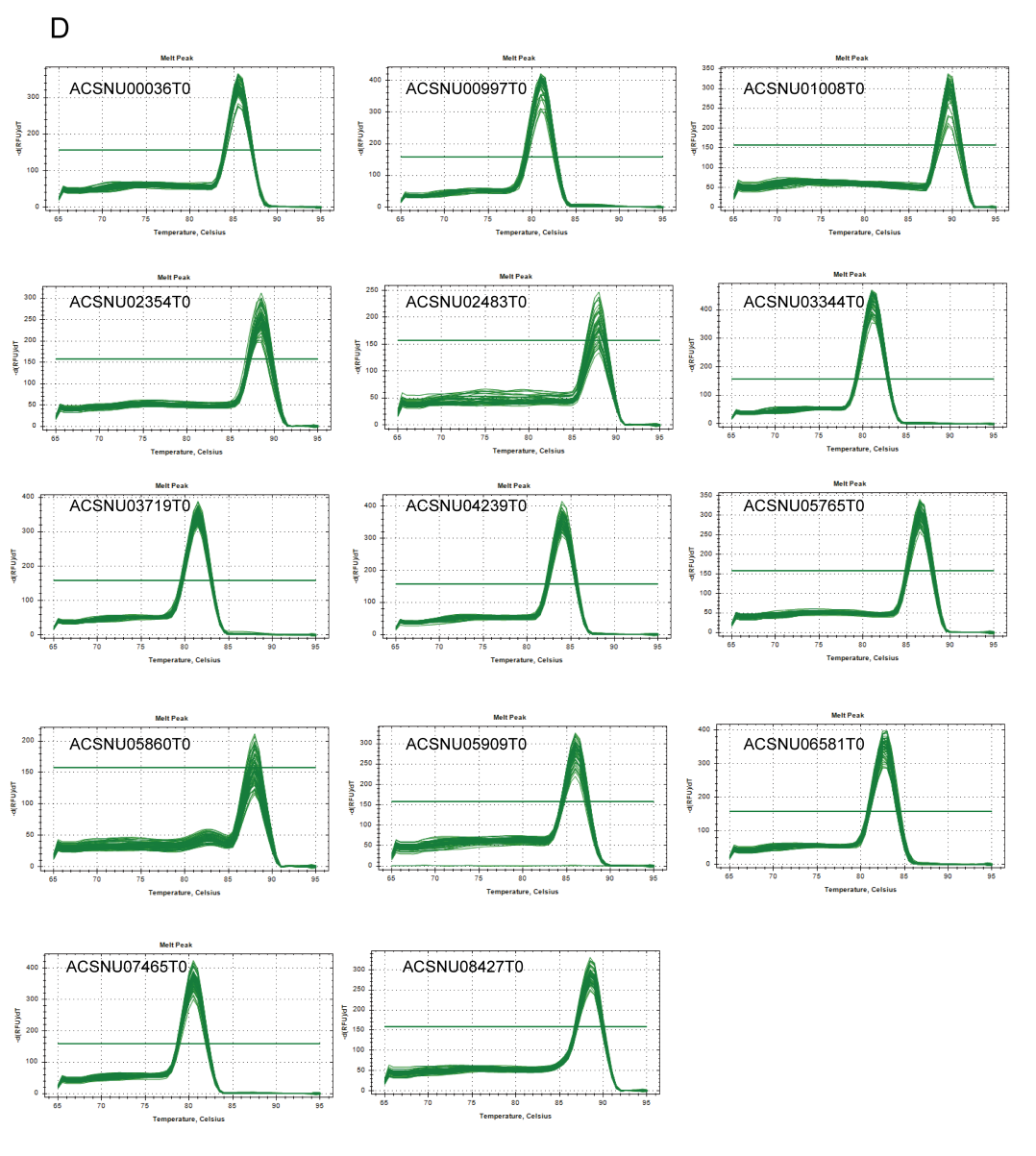


**Figure S2** qRT-PCR melting curve of 14 primers using different template cDNA. (A) Melting curve of 14 primers using templates of 9 tissues of NEW. These genes are *ACSNU00036T0*, *ACSNU00997T0*, *ACSNU01008T0*, *ACSNU02354T0*, *ACSNU02483T0*, *ACSNU03344T0*, *ACSNU03719T0*, *ACSNU04239T0*, *ACSNU05765T0*, *ACSNU05860T0*, *ACSNU05909T0*, *ACSNU06581T0*, *ACSNU07465T0*, *ACSNU08427T0*, respectively. Each primer was detected using nine different tissues, including antennae, wing, midgut, head, thorax, abdomen, front leg, middle leg and hind leg. (B) Melting curve of 14 primers using templates of 9 tissues of nurses. Each primer was detected using nine different tissues. (C) Melting curve of 14 primers using templates of 9 tissues of foragers. Each primer was detected using nine different tissues. (D) Melting curve of 14 primers using templates of 20 different developmental stages.

**Supplementary information, Figure S3**


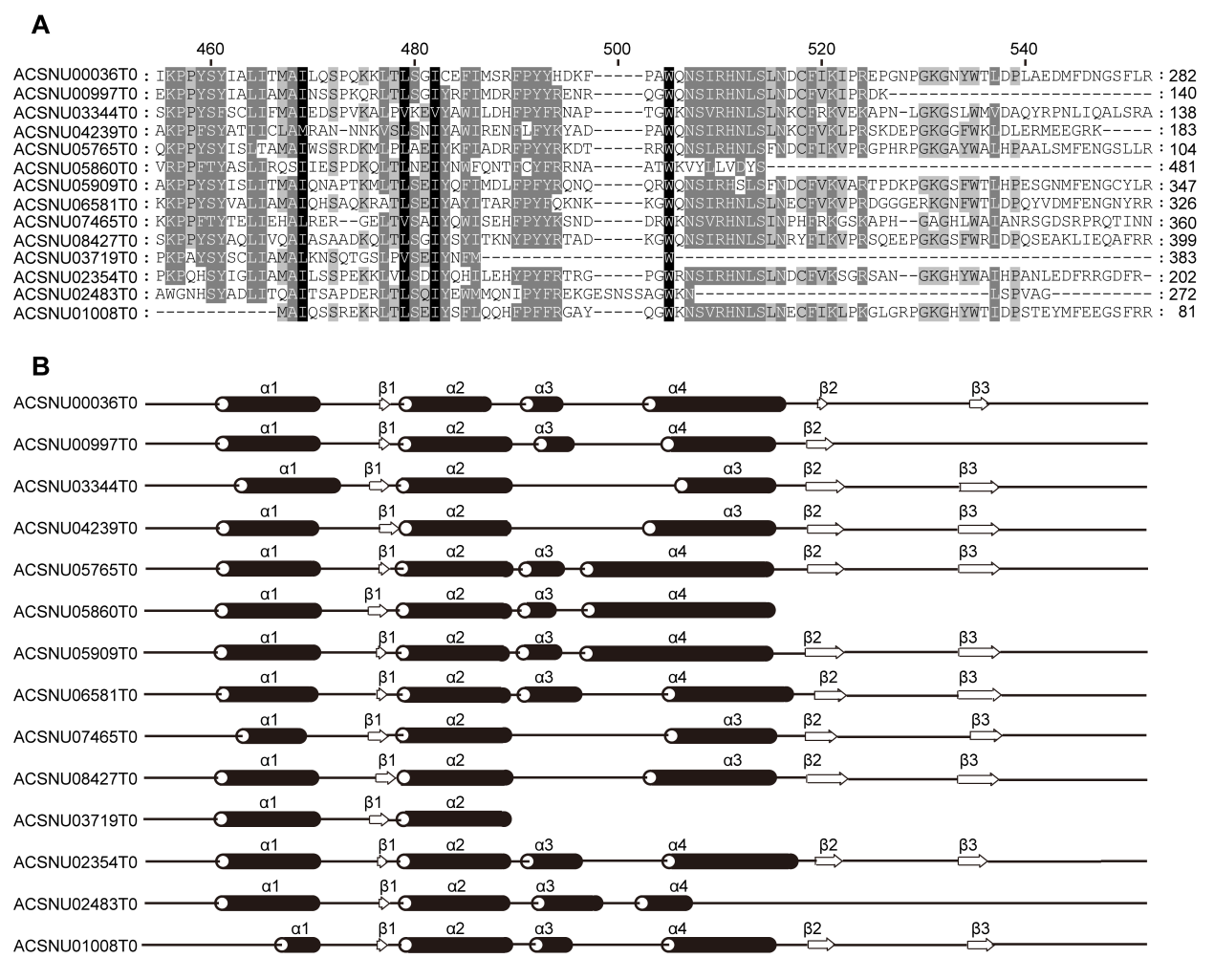


**Figure S3** Protein sequence alignment of *A. cerana* Forkhead domains and their secondary structure. (A) The alignment of the amino acid sequences of *A. cerana* Forkhead domains. (B) The secondary structure of *A. cerana* Forkhead domains. The secondary structural elements are indicated with black cylinders and arrows representing α-helices and β-strands, respectively.

**Table S1** List of primer pairs used in qRT-PCR

| **No.** | **Genes** | **Fox**  **subfamily** | **Forward primer** | **Reverse prime** | **Amplicon size(bp)** |
| --- | --- | --- | --- | --- | --- |
| 1 | ACSNU05909T0 | FoxA | GACAAATCTTATCGGAGGAGC | TGATGAACTGATAAATCTCGGAC | 127 |
| 2 | ACSNU05765T0 | FoxB | GTTCGCCAAGTTTCACCAC | AAAGTAGGAGAGCTGAGACACG | 185 |
| 3 | ACSNU02354T0 | FoxC | CGATGACGGGAATTTGGG | ATCCGACTCGATTTGGTGTT | 191 |
| 4 | ACSNU00036T0 | FoxD | CCAGGCTCCATCCTCGTA | TCGTGGTAGTAGGGGAATCG | 168 |
| 5 | ACSNU01008T0 | FoxF | GGTGTCCTCCGATTGGC | GTGGTGCTGCTGGTCCTT | 229 |
| 6 | ACSNU04239T0 | FoxJ1 | ATTTGGAGAGAATGGAGGAAG | TCGGAGGAGATACGCTACTG | 189 |
| 7 | ACSNU08427T0 | FoxK | TCGCTTACTCGGTGCTATGT | AAGATTTCAAGGTGTCGTCGT | 273 |
| 8 | ACSNU00997T0 | FoxL1 | CGTAATAAGATACAAACCACAACC | TCCAATAACTCCCTTTTCCAG | 284 |
| 9 | ACSNU06581T0 | FoxL2 | TAGCACCACCAACAGCAATC | TCTGGCGGTAATGTAAGCG | 226 |
| 10 | ACSNU07465T0 | FoxL2 | ATGGTGTCCAATAACGAATC | TTTAGACCCAAAGCAGTAGG | 176 |
| 11 | ACSNU02483T0 | FoxO | GCTTGTAAACAGCAGCGTGAT | CGGCTTCTTATTTCCCTCG | 255 |
| 12 | ACSNU03344T0 | FoxN3 | GCGTTATCTCGTGCTCCTT | ACTATCTACTTCGGTATCTGTGTTG | 174 |
| 13 | ACSNU03719T0 | FoxN4 | AAGATTCCGAGGATGAGCA | ATTCTAATACCAGACATACCGTTT | 148 |
| 14 | ACSNU05860T0 | FoxP | CGGGCTGTGAAGTTATTTGTG | TCAGAGCGGTGGAGAGATTT | 273 |
| 15 | ACSNU01044T0 | / | CCTAGCACCATCCACCATGAA | GAAGCAAGAATTGATCCACCAA | 87 |

**Table S2** Distribution of the members of the Fox gene subfamilies in *A. cerana* and other species.

Arabic number indicates the number of the members in different subfamilies in corresponding species. The data sources and orthologue descriptions could be seen in supplementary material. The Arabic numeral zero with gray shadows represents the absence of the subfamily in corresponding species. Data of *M.musculus* and *H. sapiens* are obtained from previous study (Song et al., 2015). *A. cerana*, *Apis cerana*; *A. mellifera*, *Apis mellifera*; *A.dorsata*, *Apis dorsata*; *A.florea*, *Apis florea*; *B.terrestris*, *Bombus terrestris*; *B.impatiens*, *Bombus impatiens*; *M.rotundata*, *Megachile rotundata*; *D.melanogaster*, *Drosophila melanogaster*; *B. mori*, *Bombyx mori*; *D.plexippus*, *Danaus plexippus*; *M.musculus*, *Mus musculus*; *H. sapiens*, *Homo sapiens.*

| **Fox subfamily** | ***A. cerana*** | ***A.mellifera*** | ***A.dorsata*** | ***A.florea*** | ***B.terrestris*** | ***B.impatiens*** | ***M.rotundata*** | ***D.melanogaster*** | ***B. mori*** | ***D.plexippus*** | ***M.musculus*** | | | ***H. sapiens*** |
| --- | --- | --- | --- | --- | --- | --- | --- | --- | --- | --- | --- | --- | --- | --- |
| **FoxA** | 1 | 1 | 0 | 1 | 1 | 1 | 1 | 1 | 1 | 1 | | 3 | 3 | |
| **FoxB** | 1 | 1 | 1 | 1 | 1 | 1 | 1 | 2 | 2 | 3 | | 2 | 2 | |
| **FoxC** | 1 | 2 | 1 | 2 | 2 | 2 | 2 | 2 | 2 | 2 | | 2 | 2 | |
| **FoxD** | 1 | 1 | 1 | 1 | 1 | 1 | 2 | 1 | 1 | 1 | | 4 | 6 | |
| **FoxE** | 0 | 0 | 0 | 0 | 0 | 0 | 0 | 0 | 0 | 0 | | 2 | 2 | |
| **FoxF** | 1 | 1 | 1 | 1 | 1 | 1 | 1 | 1 | 1 | 1 | | 2 | 2 | |
| **FoxG** | 0 | 1 | 0 | 1 | 1 | 1 | 2 | 3 | 2 | 2 | | 1 | 1 | |
| **FoxH** | 0 | 0 | 0 | 0 | 0 | 0 | 0 | 0 | 0 | 0 | | 1 | 1 | |
| **FoxI** | 0 | 0 | 0 | 0 | 0 | 0 | 0 | 0 | 0 | 0 | | 3 | 4 | |
| **FoxJ1** | 1 | 1 | 1 | 1 | 2 | 1 | 1 | 1 | 1 | 1 | | 1 | 1 | |
| **FoxJ2** | 0 | 0 | 0 | 0 | 0 | 0 | 0 | 0 | 1 | 1 | | 2 | 3 | |
| **FoxK** | 1 | 1 | 1 | 1 | 1 | 1 | 1 | 1 | 0 | 1 | | 2 | 2 | |
| **FoxL1** | 1 | 1 | 1 | 1 | 1 | 1 | 1 | 1 | 0 | 0 | | 2 | 2 | |
| **FoxL2** | 2 | 2 | 2 | 2 | 2 | 2 | 2 | 2 | 2 | 2 | | 1 | 1 | |
| **FoxN3** | 1 | 1 | 1 | 1 | 1 | 1 | 1 | 1 | 1 | 1 | | 2 | 3 | |
| **FoxN4** | 1 | 1 | 1 | 1 | 1 | 1 | 1 | 1 | 1 | 1 | | 2 | 2 | |
| **FoxO** | 1 | 1 | 1 | 1 | 1 | 1 | 1 | 1 | 1 | 1 | | 4 | 7 | |
| **FoxP** | 1 | 1 | 1 | 1 | 1 | 1 | 1 | 2 | 1 | 1 | | 4 | 8 | |
| **FoxQ** | 0 | 0 | 0 | 0 | 0 | 0 | 0 | 0 | 0 | 0 | | 1 | 1 | |
| **FoxR** | 0 | 0 | 0 | 0 | 0 | 0 | 0 | 0 | 0 | 0 | | 2 | 2 | |
| **FoxS** | 0 | 0 | 0 | 0 | 0 | 0 | 0 | 0 | 0 | 0 | | 1 | 1 | |
| **Total** | **14** | **16** | **13** | **16** | **17** | **16** | **18** | **20** | **17** | **19** | | **45** | **58** | |

**Supplemental File 1**

>ACSNU00036T0

MKKKKKKKTQEREKQKGDERGEANYKRGGRKSGVAISRRDTLDRFTRKEI

EREEISRVIIFNENREAREEIECAWRCAREAITMEPRGMMMSNLSCDGCA

DSDRDSDSSSMIVDPVSLDKNDLSPSQSSSSPIIPSSPVPPTTTSSRNRG

GSRSKKSYSMMSGGNGQQKGNASGQAPSSYGNDKMSSSLIKPPYSYIALI

TMAILQSPQKKLTLSGICEFIMSRFPYYHDKFPAWQNSIRHNLSLNDCFI

KIPREPGNPGKGNYWTLDPLAEDMFDNGSFLRRRKRYKRPPPHYVLRDRA

IMATFAICGDRGPCPGGGGGHPGALAYPSAAYLSPPPGLPLLDFSPSTLE

ALKLGGFLEPPPPLYKPVPITAPPIRQIDPAPTRTTAIPSGHPPAEKKRN

FSIDALIGKQAANEQNCGGLLDLSPSEHREIRSQASAFSPLNDGETEEEE

EKRRRKETRRGVGL

>ACSNU00997T0

MESNSYQNHPFSISKIYSCDLSRGCELWSTEETFPTMCPLPNYYSLVPDI

LNPRSIPLSWDLPVIRYKPQPRYEKPPYSYIALIAMAINSSPKQRLTLSG

IYRFIMDRFPYYRENRQGWQNSIRHNLSLNDCFVKIPRDKVVGNDNAEDQ

AGKGSYWTLDPSASEMFEHGNYRRRRMRMQKGFIQDNKQMEQDRAMVSVS

SEILSNLKCKEKNEQECKTVDTKLNAEGNTRNSKIIYTEAQGHCVKSMMF

TIENILRKSTNKTPV

>ACSNU01008T0

MAIQSSREKRLTLSEIYSFLQQHFPFFRGAYQGWKNSVRHNLSLNECFIK

LPKGLGRPGKGHYWTIDPSTEYMFEEGSFRRRPRGFRRKCQALKPQYPQY

FSGSGPVQAAGYENLAPGAVEYANGYQNQYQNYQEYAMYAPGGAVSSDWP

YPEAAYKAAPPIAEVTYKTTEVTYKTGESSVYRNGEIVAFKSEPGYVARS

QEQLTYRPADGFAVKDQQHHPEPVYKESEAIMSYKCPSNPAPTTQAPGQD

YYVGYGLAGVNSNVNVGMQAIQEQTGSSSPAGNVNSPHSGCQTPVTDNGI

KMQCSNSNSNSSGGGLIDRKPSYFGHPAGSVTLSSLSSLSSLNLSNIGGL

SSGLSISNIPGTVSSNVHHTTTAPPTTMYYDPIKYSMSNV

>ACSNU02354T0

MCSNESPPPAKEPLAVGLGNPMTGFLPGLEHYRLQLYHYAMAERLRLAQQ

LHPQHPGVGHPPSTAPAHLGMGPGFPAPLPLYPAAAAAAGYPSRLALSMA

LLHPHHQRIPEEPKPQHSYIGLIAMAILSSPEKKLVLSDIYQHILEHYPY

FRTRGPGWRNSIRHNLSLNDCFVKSGRSANGKGHYWAIHPANLEDFRRGD

FRRRKAQRKVRRHMGLAVDEEPDSPSPPPLPATPPPPATLGPPVASQPMT

GIWAPHHHHPHHQHQQQQQQQQQTFQSQAVRQSSSFQPSRKRQFDVASLL

APDDQHQIESDLRSAKSRRFSCSEDEMHDLQEADQDEEDVDVDVVAETNA

LPSPNTPSASPDSKLVSPTTTHWTNQGIQSTGQQLPGLHLQNHLQARNYY

VAATSSAGPTGPICMTIYQRLLLDVIDLLRVNNTVQYACITFEL

>ACSNU02483T0

MKNRKKYSHFGVETKIILLIAFSIGEIFSINSEQNCARKTIQHEHTFDTP

KDFAKVCREETAKQQSRRFSPRSGPSSLSKHGHVTDGAACKQQRDICKGT

APFEVLAVKVDYMEETNMMDSVPVSASGSKTGLQPQQGVPYDLESGFEPQ

TRARSHTWPLPSPEEYIEGNKKPNVSVRKEGVEDVESGTPPQHGSLGQGT

GLLPVKKNSSRRNAWGNHSYADLITQAITSAPDERLTLSQIYEWMMQNIP

YFREKGESNSSAGWKNLSPVAGMSPSYQQSEPSPTTLGSNQQQTLQYMMQ

TQQCQQQQLPQRQQQSQTGKFHMSRTSGRIDDYL

>ACSNU03344T0

MESVKNHYSISQNNQTKISLNNNNLPVSNRNKHPTHIPYDPHLHRNSKPP

YSFSCLIFMAIEDSPVKALPVKEVYAWILDHFPYFRNAPTGWKNSVRHNL

SLNKCFRKVEKAPNLGKGSLWMVDAQYRPNLIQALSRAPFPPPTVQTLSS

PEKPQKKNISTRLPDPTLFPYLSKRLASSNIIDNTDTEVDSDVDAAAAAM

LSFKHGPIILNHNKDRKRKVPESEMLVPVITRSSSEDHTYSCITSVRQES

KYPRKETNTDFDEQRKIAEGADALLNLAGVTTPLNHGRAHHIQSINSTSK

SESSSKLKKRSASNDYSNMSEKRRKHWPKWSEGKRYLKQII

>ACSNU03719T0

MLDTESRHGTELVQGPTRDPWLTMDYYLGTDSLSLQEMLDVDIKCEIEGV

IGGHTELGFNFTDMSALEMDDDPIGCQNDLSGWFGSTSLLNGNSNSSNSN

FNLDLSGSDAASIMVNPNSVMPHMALRSPTPNNNRRHFSFSPKKDIKEKI

DIEEEIENEGSNYTENENENENENENENENENENENENENENENENENEN

ENENENENEIENENENENENENENEQENEIENDTETEQYENDMTETEEDS

EDEQEVSKSVTPSKSKTISISATKMTSPQYTKAQTTPKINGMSGIRIQNF

KVLQSSNQMPQHVRKQIYNNNVHINPGSTTISTVKREKEFDLSDYDDKPY

PKPAYSYSCLIAMALKNSQTGSLPVSEIYNFMW

>ACSNU04239T0

MVKMKGESIDVEKRMNRDSPSMKENMHNDDCDVEAELTSLSWLQSLDITS

ASSLPTPPCSPSPPPVTRQPRKKLSPLIKAELDLAANADKYRTDPDAKPP

FSYATIICLAMRANNNKVSLSNIYAWIRENFLFYKYADPAWQNSIRHNLS

LNKCFVKLPRSKDEPGKGGFWKLDLERMEEGRKSKRRATMSQRIRGSKKL

TSGKTMVTNDAQSNCAPSINCPSTLYETPPSSVSPPIPDCLSEIPDSTQV

SLSEDDLTGLLIATAGWDENQLDLLDSLLDTLCNVPKFDT

>ACSNU05765T0

MPRPSRDTYGDQKPPYSYISLTAMAIWSSRDKMLPLAEIYKFIADRFPYY

RKDTRRWQNSLRHNLSFNDCFIKVPRGPHRPGKGAYWALHPAALSMFENG

SLLRRRKRFKLHKPDKELLKSELQALASAMPPPHSESSPVLSINPNAQTS

SLTVANLHRLRDDLLRWELQERRLAMTSGGNSSPSFTTPEAGSSYYLLSP

EVRQRLAGTDEILRGYESNLLQAGSWSFPGFQVSPYVSQLSYFQTDIPES

RQICAGNAEAGVTEKTDSRLFLETARGATIESADGKVRCPPLEAGISNQT

SIQPGQKKKKKPFTIENIIAPDDEQISSNSEDEKRSSGHLLVPRPLYAGF

SFGLPTSKVPYETAT

>ACSNU05860T0

APPSQNPSQMMISPASGIHQMQQLLQQHILSPTQLQSFMQQHSLYLQQQQ

QQHHQDSSSEHASNQERFGYFSSLKDHQHQFAELGRKKLEQAIQQLQEQL

QLNVIQQTHLLQTADKKKASAPLQQLALQQQRLIQQLQITQSQYLLQQGL

GLQGHNPSSGLQPGEGLPMWKSDTSDGPESHQNSNVPKSVAGLNVSSRRS

DMNGTTPLDEKPLDVSSNDKVHPLYGHGVCKWPGCEVICEDYQAFLKHLN

TEHTLDDRSTAQARVQMQVVSQLEIQLQKERDRLTAMMHHLHVAKQMASP

EPPKSSESSTGSSIPKLNLSTALMSQPPPNFGVSQVSPVSMSALVSAVRS

PAGGQLPPSAGAPMPPIPNMSNMSGMPPLPNMPGSMPTMPTMPSMAGPIR

RRISDKSALSLAGEIQRNREFYKNADVRPPFTYASLIRQSIIESPDKQLT

LNEIYNWFQNTFCYFRRNAATWKVYLLVDYSKRKKKRKEKNLFSLDTCGT

RGL

>ACSNU05909T0

MGGRDGSRGRVDSDWRDKGWKIRRVSYYQSPVKWDKTKIRVEAAAEGNGG

AMKRGGKTRWVGKETMASRWTRGVLEVEDNPGGVYDRETARMKTKQQPAT

PAPVSSMTMLQSQKLYSDAGSLGGAMTSAAMSTMGSMAPTYSSINSVGCM

PMGMSMGVGVGPSCSPQGAGGFNMSTMSSAMGMASMGGGGMGGYGSASMG

GGGACMSAVGYGPLTPGGGAGVTRDPLSLAEPDSPNSALQRARTDKSYRR

SYTHAKPPYSYISLITMAIQNAPTKMLTLSEIYQFIMDLFPFYRQNQQRW

QNSIRHSLSFNDCFVKVARTPDKPGKGSFWTLHPESGNMFENGCYLRRQK

SLGDGSTAAATAAAAAAAAGIAETSAAEVRGERGGGGLLARRARERLRRG

GGGGGGGRGRGGYVRRRCEKRKMVGGDGRGNRREQYEGDVTVVKRSPLKF

HEEITVNDRSGWRVDRRKNTHIRSTSGRNLRSSVIERINTGLVEKQRRAD

TISLFPQPRYFFTSRGTAVKKLDGKLIAVFLLISVVIIKQERCGQGKYLR

QDSGPIATIKPLSSSNVSGGEKVGKFRRFAIFANDAIVESAPFRFAEAAA

ARSADQQWKIGWLGAWQRTSAADHRGACLDLPIRQTGRIAEQSRYNGRLY

TLSTSSFFDKNSSSLLGSWVQEERGDKIEQIRLVRNRIATFATRENEEMC

YEERNVATLQRSNNKHAAKKREGRQISRIAFFLSAEAIPRNDSNDQANAY

IHGSVIRNHVPFEATREEKKMKGNDDLPFLTNSNAIRYKSLHNRSLPKEI

KFEFEVSIRNEY

>ACSNU06581T0

MEVAYEDRIFGCSSHKGRRTRRGRSLIMEGLPAYWEDLAVQHSVNAAATA

SIFMNYDLDKPRMVSHVIPVAGELQQGNGNLGVTGLGGMQESSHSLKIKQ

EPFQVSPPSPLPPLHQVSSFVSEMQNGCIGVASKELDSSVNLTRGLTSHH

TSLSHHHSHHSLHSPNSAVSMHSTGSTHHHLDEKGSSPTGALHSTTNSNP

STPSTPSTPTTVADGSSTATVTGNGNTNNDSASKKPPYSYVALIAMAIQH

SAQKRATLSEIYAYITARFPYFQKNKKGWQNSIRHNLSLNECFVKVPRDG

GGERKGNFWTLDPQYVDMFENGNYRRRRRMKRPYRNAPYKPLFGDPFTPT

HVHLGPRNLFGPSPPSYAPSTYTRYDTSAWSLQQSQLPYSHCPSVSASLI

SVIIFIIYPKIEVRSIITKFGFFLKLQPQLQPMQSMQIPNMNGYSQLGTS

LNKAFQGNYLDVPGGTTSSSGSMTSGSFGSSFARRHDAAMTQETVPTRCY

WPEMVNVKEEPGTVAVSSTSVGSVGVPSGMMGSTTPSGVSTTGFAPMEFQ

SRSKCFINCRRKR

>ACSNU07465T0

MIGPSSGIGDVPEHELSDNQSRGGKKRIFEDLDIENFCEEVQNGHKRYQY

QEIEAPMEMVASNQVINDTASLFSPLSAQEAVDECSVGRLEVLLVDGTNC

TWTTVSELESQQSIADITASPSTSTSSSENRQEQVIQEVQIEETSYPIDC

DYWEPVVTVQSSNMQQNKPMMPSGSNQQQQFDDQETGNLSWLLDFKLDPF

IEAADEKSTVSLSKDIHNGNKARMNGRSYGSAYINDAKKSYNENGSLHQE

SNHNYSSLDNRNFASSRCNGPKKPPFTYTELIEHALRERGELTVSAIYQW

ISEHFPYYKSNDDRWKNSVRHNLSINPHFRKGSKAPHGAGHLWAIANRSG

DSRPRQTINNSIINSSTKQISKNNIEIENLRKNVSQMNPIDEVEAATASI

TQQSNEEETENIVNSVTLEHCAEEILSGIKKEVEVQYLVPMMVSNNESTH

PNQQTQELHYPVKESDFLNPVSKEVVAEECGLISEGYLVTDLNPTALGLN

MIEPEIITPENLFGEELSFQFYELSSPSQIQSA

>ACSNU08427T0

MYLSTYLSKYLSIYLYLSTSPFSPSLILSQRRVRLLGAMSTTYSRTQESD

AWALLALKSAPASPTKMQWNPEAKGAPIARLEGREFEYMVRQRRITIGRN

SSKGEVDVNMGHSSFISRRHLEIFYDHPFFFMTCNGKNGVFVDGVFQRKG

APAFQLPKTCTFRFPSTNIRLVFQSLVDEQEQSNIPVSSPPKHRAPLPPL

RINIPDTGYSSPFPSPTGTISAANSCPASPRAGQGRRNISADLQMVAVYA

AAVANDPQNSNMERHDGGQSSNRQISPELGVESRYRSGSSSGPNGTTAHC

SPPKDDSKPPYSYAQLIVQAIASAADKQLTLSGIYSYITKNYPYYRTADK

GWQNSIRHNLSLNRYFIKVPRSQEEPGKGSFWRIDPQSEAKLIEQAFRRR

RQRGVPCFRAPFGLSSRSAPASPSHVGISGLMTPECLSREASPGPESYPD

STVSSPAGQLTSQSAPGSPGHPYASSSQSSHKGRLMQQITVVTNGVSGDT

TREDSYIFSDKYVSGNTTEEHSLSPAGQYSPAPVIVQTTYNYSGSFIGPD

AGVGVAKRSHEESDSSPGSPAPLAIVESPEPPEHQQPSTKRQRVHEMDDH

>GB40150

MSRKGGKDKSFPLCTFDGQTDSVFGKAYTRPNPFKEVPRARHKLLLRLVPDDGIMDHDTD

GDGAINLSTSQRPSAATTPNGDTPSYGQDQQDNDQATSLFAALKQQQQQQQPRDTVPSSR

ERVENRDRDRLSVRNRENNRNEIGGGVTEQSQPAQQQQQQQQQQQQELTIEYQSNGKLSP

AGHAVTAAPMTQQKQPIITQQSQQPSSGAPGPQPSPHQSPQAPQRGSPPNPSQGPPPGGP

PGAPPSQNPSQMMISPASGIHQMQQLLQQHILSPTQLQSFMQQHSLYLQQQQQQHHQDSS

SEHASNQERFGYFSSLKDHQHQFAELGRKKLEQAIQQLQEQLQLNVIQQTHLLQTADKKK

ASAPLQQLALQQQRLIQQLQITQSQYLLQQGLGLQGHNPSSGLQPGEGLPMWKSDTSDGP

ESHQNSNVPKSVAGLNGLLNSTVSSRRSDMNGTTPLDEKPLDVSSNDKVHPLYGHGVCKW

PGCEVICEDYQAFLKHLNTEHTLDDRSTAQARVQMQVVSQLEIQLQKERDRLTAMMHHLH

VAKQMASPEPPKSSESSTGSSIPKLNLSTALMSQPPPNFGVSQVSPVSMSALVSAVRSPA

GGQLPPSAGAPMPPIPNMSNMSGMPPLPNMPGSMPTMPTMPSMAGPIRRRISDKSALSLA

GGLYDEGTVRRRVAVDRSGIDINEGLPYMLERAGLDVQQEIQRNREFYKNADVRPPFTYA

SLIRQSIIESPDKQLTLNEIYNWFQNTFCYFRRNAATWKNAVRHNLSLHKCFMRVENVKG

AVWTVDEVEFYKRRPQRACSTTGGVPSKSPTLTHSPTMYGDALNANLQAALGDSNMGFLN

NSMCTSTTTSPDKEHVLAHNDLMSPLDEPAVHIKQEGQSPEGGKLTRLIKRELVDAPADQ

EGDDDQVDEREYPESHGHDSGQDEDMAEDLSMAPDIMTPEDQIEA

>GB41160

MIGPSSGIGDVPEHELSDNQSRGGKKRIFEDLDIENFCEEVQNGHKRYQYQEIEAPMEMV

ASNQVINDTASLFSPLSAQEAVDECSVGRLEVLLVDGTNCTWTTVSELESQQSIADITAS

PSTSTSSSENRQEQVIQEVQIEETSYPIDCDYWEPVVTVQSSNIQQNKPMPSGSNQQQQF

DDQETGNLSWLLDFKLDPFIEAADEKSTVSLSKDIHNGNKARMNGRSYGSAYINDAKRSY

NENGSLHQESNHNYSSLDNRNFASSRCNGPKKPPFTYTELIEHALRERGELTVSAIYQWI

SEHFPYYKSNDDRWKNSVRHNLSINPHFRKGSKAPHGAGHLWAIANRSGDSRPRQTINNS

IINSSTKQISKNNIEIENLRKNVSQMNPIDEVEAATASITQQSNEEETENIINSVTLEHC

AEEILSGIKKEVEVQYLVPMMVSNNESTHPNQQTQELHYPVKESDFLNPVSKEVVAEECG

LISEGYLVTDLNPTALGLNMIEPEIITPENLFGEELSFQFYELSSPSQIQSA

>GB41890

MLDTESRHGTELVQGPTRDPWLTMDYYLGTDSLSLQEMLDVDIKCEIEGVIGGHTELGFN

FTDMSALEMDDDPIGCQNDLSGWFGSTSLLNGNSNSSNSNFNLDLSGSDAASIMVNPNSV

MPHMALRSPTPNNNKRLFSFSPKRDMKEKIDFEEEIEGRGRYRKNENENENENENENENE

NENENENENENENENENENENENENENENEIENENENENENENENEQENEIENDTETEQY

ENDITETDEEDSEDEQEVSKSVTPSKSKTISISAAKMTSPQYTKAQTTPKINGMSGIRIQ

NFRVLQSSNQMPQHVRKQIYNNNVHINQGSTTIGTVKREKEFDLSDYDDKPYPKPAYSYS

CLIAMALKNSQTGSLPVSEIYNFMCEHFPYFKTAPNGWKNSVRHNLSLNKCFEKIEKPAG

NGNQRKGCLWAINPAKVAKMDEEVQKWSRKDPLAIKKAMIYPDHLELLERGEMKYAGSGD

VSEETESSGDEAVEESTTYEESIHGHITANSVTDSYDESSQDCDIDIHEHLYDEIDIEDN

KEALHMHLNISKQEPFEYELNSGTKRQKTLTGAIQGNYVYQPVTTSRRKTPLLVRAGAGN

SSFLKID

>GB41986

MPRPSRDTYGDQKPPYSYISLTAMAIWSSRDKMLPLAEIYKFIADRFPYYRKDTRRWQNS

LRHNLSFNDCFIKVPRGPHRPGKGAYWALHPAALSMFENGSLLRRRKRFKLHKPDKELLK

SELQALASAMPPPHSESSPVLSINPNAQTSSLTVANLHRLRDDLLRWELQERRLAMTSGG

NSSPSFTTPEAGSSYYLLSPEVRQRLAGTDEILRGYESNLLQAGSWSFPGFQVSPYVSQL

SYFQTDIPESRQICAGNAEAGVPEKTDSRLFLETARGATIESADGKVRCPPLEAGISNQT

SIQPGQKKKKKPFTIENIIAPDDEQISSNSEDEKRSSGNLLVPRPLYAGFSFGLPTSKVP

YETAT

>GB42758

MEPRGMMMSNLSCDGCADSDRDSDSSSMIVDPVSLDKNDLSPSQSSSSPIIPSSPVPPTT

TSSRNRGGSRGKKSYSMMSSSGNGQQKGNGSGQAPSSYGNDKMSSSLIKPPYSYIALITM

AILQSPQKKLTLSGICEFIMSRFPYYHDKFPAWQNSIRHNLSLNDCFIKIPREPGNPGKG

NYWTLDPLAEDMFDNGSFLRRRKRYKRPPPHYVLRDRAIMATFAICGGDRGPCPGGGGGH

PGALAYPSAAYLSPPPGLPLLDFSPSTLEALKLGGFLEPPPPLYKPVPITAPPIRQIDPA

PTRTTAIPSGHPPAEKKRNFSIDALIGKQAANDQNCGGLLDLSPSEHREIRSQASAFSPL

V

>GB43876

MVSHVIPVAGELQQGNGNLGVTGLGGMQESSHSLKIKQEPFQVSPPSPLPPLHQVSSFVS

EMQNGCIGVASSKELDSSVNLTRGLASHHTSLTHHHSHHSLHSPNSAVSMHSTGSTHHHL

DEKGSSPTGALHSTTNSNPSTPSTPSTPTTVADGSSTATHSAQKRATLSEIYAYITARFP

YFQKNKKGWQNSIRHNLSLNECFVKVPRDGGGERKGNFWTLDPQYVDMFENGNYRRRRRM

KRPYRNAPYKPLFGDPFTPTHVHLGPRNLFGPSPPSYAPSTYTRYDTSAWSLQQSQLPYS

HCPSLQPQLQPMQSMQIPNMNGYSQLGTSLSNYLDVPGGTTSSSGSMTSGSFGSSFARRH

DAAMTQETVPTRCYWPEMVNVKEEPGTVAVSSTSVGSVGVPSGMMGSTTPSGVSTTGFAP

MEFQSRSKCFM

>GB44229

MVNMEGSGEQQTVTASPPPRVPLKSSFSIRSILPEACAGTPAPPASRTASPEISHVDESE

DSSDLDVTGDGGAETPPLDCSRNAATSANSSAEHKDCKDRQNSDEKKKCEKPPYSYNALI

MMAIRQSPEKRLTLNGIYEYIMRHFPYYENNKQGWQNSIRHNLSLNKCFVKVPRHYDDPG

KGNYWMLDPSSEDVFIGGTTGKLRRRTTAASRSRLAAFKRSVVLGGLYPSAYAPPGWPAS

LYTLPYLHRAAAAGYPPATGAYSTPAGYPASLLPGAAATGSTTSLPCKPQPLPAAAAPPQ

HGPFSMERLLQPPTAAGYPPGIAASAIPVSATPYDFYSTLRSLAAHQHQSTAAAFAHNQQ

PTTRYHQAPLLSQPTSATASPGSSPEPMSPHSPPVTVCNSVQQPRSLPHSPPQLLLKPIT

VLTGRQS

>GB45298

MTSKYGVSISWVLCRVCLRCPSLENLRRLPSVYANTDKEDTSHNMPPDRPDASDVETGIG

LSTQVFNFGDTVYTMKEKENASVFVETNSTDELSMSEKSIEKNSQSDDDLTSLSWLHQQN

LLKGLDISNPTKEMKNENVLNNNVCDDMADYSENTNSISSLDDGYCPGDNNSKINNTTSH

GNNQNYQVSSKNGQKSMQIFQESVKNHYSISQNNQTKISLNNNNLPVSNRNKHPTHIPYD

PHLHRNSKPPYSFSCLIFMAIEDSPVKALPVKEVYAWILDHFPYFRNAPTGWKNSVRHNL

SLNKCFRKVEKAPNLGKGSLWMVDAQYRPNLIQALSRAPFPPPTVQTLSSPEKPQKKNIS

TRLPDPTLFPYLSKRLASSNIIDNTDTEVDSDVDAAAAAMLSFKHGPIILNHNKDRKRKV

PESEMLVPVITRSSSEDHTYSCITSVRQESKYPRKETNTDFDEQRKIAEGADALLNLAGV

TTPLNHSRAHHIQSINSTSKSESSSKLKKRSASNDYSNMSEKRRKHWPKWSEGKRYLKQI

I

>GB48098

MHTLFSEQNAYYRHATAVPVGMGTPSYPGVGAAPGYYEQYRYGGYATAAGYPVSGITQQH

IHHPGKDMVKPPYSYIALIAMAIQNAPDKKITLNGIYQFIMERFPYYRENKQGWQNSIRH

NLSLNECFVKVPRDDKKPGKGSYWSLDPDSYNMFDNGSYLRRRRRFKKKDALKEKEEALK

RQGLVPEKQRQNQEETKPSNIVIPPPSDASGTKKLASLETTLCKPKREPVNETGSHCMAV

QAKYGLHSPIQDTKTAVVTTTTAVAVAGQSVIQTALGHQVHQAHQVHQDAGIQDVSMGLD

PTSFSVDALMTTRENSAALMTRENQHHTHHPHGLQHPHPHSHSIMTSRESMGAGVVSRDH

LPSVCTTTTTTTAAVAAMMATTGYGHTSSVSRSNGSPPGTMYAPYCTPTAGYIMDHAEYN

SRNHNNTAGHSQWYQEAASPDAAAIYQDTQSPSCQLYRSSPPTPLSSSAAPSPPLPHHRG

LEEHHTDHYDPSLVYVKQEWIPCTEDLAKEWN

>GB48100

MDQTVIVPSTPRGGPSSMDNGSRLAMKNETDSSVHMHHPQDHQSSVTHSNSSAPPTVVPR

PSNLTVSNGPLSQDQAIDALMCGQSTAELPRKPGARRQEKPPYSYIALIVMAIQSSREKR

LTLSEIYSFLQQHFPFFRGAYQGWKNSGWKNSVRHNLSLNECFIKLPKGLGRPGKGHYWT

IDPSTEYMFEEGSFRRRPRGFRRKCQALKPQYPQYFSAGGGPVSVQAAAGYENLAPAAVE

YANGYQNHQYQNYQEYAAMYAAPGGAVSSDWPYPETTAYKTAPPPPPIAEVTYKTTEVTY

KTDESSVYRNGEIVAFKSEPGYVAARSQEQLTYRTADGFAVKDHAAEPQVYKENEAMMAS

SYNRASNPAQPGQDYYVGYGLAVSNANNVGMQGIQEQTGNSSPVGNVNSPHSGCQTPVTD

NGIKMQCSNSNSNSSGGGLIDRKPSYFGHPAGSVTLSSLSSLSSLNLSNIGGLGSGLSIS

NIPGAAVSSNIHHATTTPPTTMYYDPIKYSMSNV

>GB48110

MESNSYQNHPFSISKIYTCDLSRGCELWSTEETFPTMCPLPNYYSLVPDILNPRSIPLSW

DLPIIRYKPQPRYEKPPYSYIALIAMAINSSPKQRLTLSGIYRFIMDRFPYYRENRQGWQ

NSIRHNLSLNDCFVKIPRDKVVGNDNAEDQAGKGSYWTLDPSASEMFEHGNYRRRRMRRQ

KGFIQDNKQMEQDRTMVSVSSEILSNLKCKEKNEQECKMVDTKNAEGNTRNSKIIYTEAQ

GHCVKSMMFTIENILRKSTNKTSV

>GB48301

MKNRKKYSHFGVETKIILLIAFSIGEIFLINSEQDCAQKTTQHEYTFDTSKDFAKVCRVE

IAKQQSRRFSPRSGPSSLSKHGHVTDGAACKQQRDICEETAPLEVLAVKVDYMEETNMMD

SVPVSASGSKTGLQPQQGVPYDLESGFEPQTRARSHTWPLPSPEEYIEGNKKPNVSVRKE

GVEDVESGTPPQHGSLGQGTGLLPVKKNSSRRNAWGNHSYADLITQAITSAPDERLTLSQ

IYEWMMQNIPYFREKGESNSSAGWKNSIRHNLSLHSRFMRVQNEGTGKSSWWMINRDAKP

GKSRRRAITMETSKFEKRRGRVRKKIEALKNGGLQADTTTSPSNSVNEGLDLFPDSPLQS

GSGFQLSPDFRPRASSNASSCGRLSPITAIPRKPEWTPTYTLSYSPEQLAGSLAETMKLE

SYQMYHTTQPSHQHHTGPPPSYYESQYQRSNSLSSGSSSFALQPTPQSANQQRCPIHGLQ

PCACQMNLSPVAGMSPSYQQSEPSPTTLGNNQQQTLQYMMQTQQCQQQQLPQRQQQSQTG

VTDSSSSQTSTPCDPTPSTMMGQLMGALNNSTLLDDLNINIESLHGGFDCNVEEVIKHEL

SMDGTLDFNFQQSVMGTAAIQVSDNNIGQNGAVSQNNVIGTTTTGNATAGVYVSTNAATP

AAPPSWVH

>GB50277

MVKMKGESIDVEKRMNPDSPSMKENMRNDDCDVEAELTSLSWLQSLDITSASSLPTPPCS

PSPPPVTRQPRKKLSPLIKAELDLAANADKYRTDPDAKPPFSYATIICLAMRANNNKVSL

SNIYAWIRENFLFYKYADPAWQNSIRHNLSLNKCFVKLPRSKDEPGKGGFWKLDLERMEE

GRKSKRRASMSQRIRGSKKLTSGKTTMVTNDAQSNGAPSINCPSTLYETPPSSVSPPIPD

CLSEIPDSTQVSLSEDDLTGLLIATAGWDENQLDLLDSLLDTL

>GB50374

MCSNESPPPAKEPLAVGLGNPMTGFLPGLEHYRLQLYHYAMAERLRLAQQLHPQHPGVGH

PPSTAPAHLGMGPGFPAPLPLYPAAAAAAGYPSRLALSMALLHPHHQRIPEEPKPQHSYI

GLIAMAILSSPEKKLVLSDIYQHILEHYPYFRTRGPGWRNSIRHNLSLNDCFVKSGRSAN

GKGHYWAIHPANLEDFRRGDFRRRKAQRKVRRHMGLAVDEEPDSPSPPPLPATPPPPATL

GPPVASQPMTGIWTPHHHHHPPHHQQQQQQQQQQQQQQQTAFQNQGARQSSSTFQPSRKR

QFDVASLLAPDDQHHQIESDLRSAKSRRFSCSEDEMHDLQEAEQDEEDVDVDVVAETNAL

PSPNTPSASPDSKLGVSPPTATQLWTNQGIQGATGQQLAGLHLQNHLQGARNYYVAATSS

TVGPTV

>GB52677

MSTTYSRTQESDAWALLALKSAPASPTKMQWNPEAKGAPIARLEGREFEYMVRQRRITIG

RNSSKGEVDVNMGHSSFISRRHLEIFYDHPFFFMTCNGKNGVFVDGVFQRKGAPAFQLPK

TCTFRFPSTNIRLVFQSLVDEQEQSNIPVSSPPKHRAPLPPLRINIPDTGYSSPFPSPTG

TISAANSCPASPRAGQGRRNISADLQMVAVYAAAVANDPQNSNMERHDGGQSSNRQISPE

LGVESRYRSGSSSGPNGTTAHCSPPKDDSKPPYSYAQLIVQAIASAADKQLTLSGIYSYI

TKNYPYYRTADKGWQNSIRHNLSLNRYFIKVPRSQEEPGKGSFWRIDPQSEAKLIEQAFR

RRRQRGVPCFRAPFGLSSRSAPASPSHVGISGLMTPECLSREASPGPESYPDSTVSSPAG

QLTSQSAPGSPGHPYASSSQSSHKGRLMQQITVVTNGVSGDTTREDKYVSGNTTEEHSLS

PAGQYSPAPVIVQTTYNYSGSFIGPDAGVGVAKRSHEESDSSPGSPAPLAIVESPEPPEH

QQPSTKRQRVHEMDDH

>GB53404

MLQSQKLYSDAGSLGGAMTSAAMSTMGSMAPTYSSINSVGCMPMGMSMGVGVGPSCSPQG

AGGFNMSSMSSAMGMASMGGGGMGGYGSASMGGGGACMSAVGYGPLTPGGGAGVARDPLS

LAEPDSPNSALQRARTDKSYRRSYTHAKPPYSYISLITMAIQNAPTKMLTLSEIYQFIMD

LFPFYRQNQQRWQNSIRHSLSFNDCFVKVARTPDKPGKGSFWTLHPESGNMFENGCYLRR

QKRFKDEKKELTRQNNKHQQHHTGAAAAAAAVAVAAAAGHNSPTSHELAHAAKKSAASSL

HHGPQQQDDKDLHSLVTSHHHHHAAAAAAASLHQHHASLKSDAADIGGLLGPDLGAAHDE

LTAMVSRSLHPHLISEPAAALHHGMAGSLKQEPPYTAASHPFSITRLLPGATAATSPGAQ

DIKPPEMKMYEQLHQSYANFGSPHHPHAHSAPPSHHHHNGMHAASNAATPMHNMTNHHHQ

EYYQSPLYHHATSVASSSAPPPSTVATAAPGL

>XP_006607409.1[Apis dorsata]

MIGPSAGIGDVPEHELSDNQSRGGKKRIFEDLDIENFCEEVQNGHKRYQYQEIEAPMEMVASNQVINDTA

SLFSPLSAQEAVDECSVGRLEVLLVDGTNCTWTTVSELESQQNIADITASPSTSTSSSENRQEQVIQEVQ

IEETSYPIDCDYWEPVVTVQSSNIQQNKPMMPSGSNQQQQFDDQETGNLSWLLDFKLDPFIEAADEKSTV

SLSKDIHNGNKARMNGRSYGSAYINDAKRSCNENGSLHQESNHNYSSLDNRNFASSRCNGPKKPPFTYTE

LIEHALRERGELTVSAIYQWISEHFPYYKSNDDRWKNSVRHNLSINPHFRKGSKAPHGAGHLWAIANRSG

DSRPRQTINNSIINSSTKQISKNNIEIENLRKNVSQMNPIDEVEAATASITQQSNEEETENIVNSVTLEH

CAEEILSGIKKEVEVQYLVPMMVSNNESTHPNQQTQELHYPVKESDFLNPVSKEVVAEECGLISEGYLVT

DLNPTALGLNMIEPEIITPENLFGEELSFQFYELSSPSQIQSA

>XP_006607579.1[Apis dorsata]

MNYDLDKPRMVSHVIPVAGELQQGNGNLGVTGLGGMQESSHSLKIKQEPFQVSPPSPLPPLHQVSSFVSE

MQNGCIGVTSKELDSSVNLTRGLTSHHTSLSHHHSHHSLHSPNSAVSMHSTGSTHHHLDEKGSSPTGALH

STTNSNPSTPSTPSTPTTVADGSSTATVTGNGNTNNDSASKKPPYSYVALIAMAIQHSAQKRATLSEIYA

YITARFPYFQKNKKGWQNSIRHNLSLNECFVKVPRDGGGERKGNFWTLDPQYVDMFENGNYRRRRRMKRP

YRNAPYKPLFGDPFTPTHVHLGPRNLFGPSPPSYAPSTYTRYDTSAWSLQQSQLPYSHCPSLQPQLQPMQ

SMQIPNMNGYSQLGTSLTFQGNYLDVPGGTTSSSGSMTSGSFGSSFARRHDAAMTQETVPTRCYWPEMVN

VKEEPGTVAVSSTSVGSVGVPSGMMGSTTPSGVSTTGFAPMEFQSRSKCFM

>XP_006608727.1[Apis dorsata]

MPRPSRDTYGDQKPPYSYISLTAMAIWSSRDKMLPLAEIYKFIADRFPYYRKDTRRWQNSLRHNLSFNDC

FIKVPRGPHRPGKGAYWALHPAALSMFENGSLLRRRKRFKLHKPDKELLKSELQALASAMPPPHSESSPV

LSINPNAQTSSLTVANLHRLRDDLLRWELQERRLTMTSGGNSSPSFSTPEAGSSYYLLSPEVRQRLAGTD

EILRGYESNLLQAGSWSFPGFQVSPYVSQLSYFQTDIPESRQICAGNAEAGVPEKTDSRLFLETARGATI

ESADGXVRCPPLEAGISNQTSIQPGQKKKKKPFTIENIIAPDDEQISSNSEDEKRSSGHLLVPRPLYAGF

SFGLPTSKVPYETAT

>XP_006609622.1[Apis dorsata]

MDHDTDGDGAINLSTSQRPSAATTPNGDTPSYGQDQQDNDQATSLFAALKQQQQQQQPRDTVPSSREREN

RDRDRLSVRNRENNRNEIGGGVTEQSQPTQQQQQQQELTIEYQSNGKLSPAGHAVTAAPMTQQKQPIITQ

QSQQPSSGAPGPQPSPHQSPQAPQRGSPPNPSQGPPPGGPPGAPPSQNPSQMMLSPASGIHQMQQLLQQH

ILSPTQLQSFMQQHSLYLQQQQQQHHQDSSSEHASNQERFGYFSSLKDHQHQFAELGRKKLEQAIQQLQE

QLQLNVIQQTHLLQTADKKKASAPLQQLALQQQRLIQQLQITQSQYLLQQGLGLQGHNPSSGLQPGEGLP

MWKSDTSDGPESHQNSNVPKSVAGLNGLLNSTVSSRRSDMNGTTPLDEKPLDVSSNDKVHPLYGHGVCKW

PGCEVICEDYQAFLKHLNTEHTLDDRSTAQARVQMQVVSQLEIQLQKERDRLTAMMHHLHVAKQMASPEP

PKSSESSTGSSIPKLNLSTALMSQPPPNFGVSQVSPVSMSALVSAVRSPAGGQLPPSAGAPMPPIPNMSN

MSGMPPLPNMPGSMPTMPTMPSMAGPIRRRISDKSALSLAGGLYDEGTVRRRVAVDRSGIDINEEIQRNR

EFYKNADVRPPFTYASLIRQSIIESPDKQLTLNEIYNWFQNTFCYFRRNAATWKNAIRTNLSLHKCFVRY

EDDFGSFWMVDDAEFVKRRHLSRGRPRKYDPTPSPTPPHLSAQGVPSKSPTLTHSPTMYGDXSNAI

>XP_006609859.1[Apis dorsata]

MLDTESRHGTELVQGPTRDPWLTMDYYLGTDSLSLQEMLDVDIKCEIEGVIGGHTELGFNFTDMSALEMD

DDPIGCQNDLSGWFGSTSLLNGNSNSSNSGSDAASIMVNPNSVMPHMALRSPTPNNNRRHFSFSPKKDLK

EKIDIEEEIENEGRNYTENENENENENENENENENENENENENENENENENENENENENENENEIENENE

NENENENENEQENEIENDTETEQYENDITETEEDSEDEQEVSKSVTPSKSKTISISATKMTSPQYTKAQT

TPKINGMSGIRIQNFKVLQSSNQMPQHVRKQIYNNNVHINPGSTTISTVKREKEFDLSDYDDKPYPKPAY

SYSCLIAMALKNSQTGSLPVSEIYNFMCEHFPYFKTAPNGWKNSVRHNLSLNKCFEKIEKPAGNGNQRKG

CLWAINPAKVAKMDEEVQKWSRKDPLAIKKAMIYPDHLELLERGEMKYAGSGDVSEETESSGDEAVEEST

TYEESIHGHITANSVTDSYDESSQDCDIDIHEHLYDEIDIEDNKEALHMHLNISKQEPFEYELNSGTKRQ

KTLTGAIQGNYVYQPVTTSRRKTPLLVRAGAGNSSFLKID

>XP_006611159.1[Apis dorsata]

MHTLFSEQNAYYRHATAVPVGMGTPSYPGVGAAPGYYEQYRYGGYATAAGYPVSGITQQHIHHPGKDMVK

PPYSYIALIAMAIQNAPDKKITLNGIYQFIMERFPYYRENKQGWQNSIRHNLSLNECFVKVPRDDKKPGK

GSYWSLDPDSYNMFDNGSYLRRRRRFKKKDALKEKEEALKRQGLVPEKQRNQEETKPSNIVIPPPSDTSG

GKKLGTLETTLCKPKREPVNDTGSHCMAVQAKYGLHSPIQDTXSTTTTAAVAVGGQSVIQTALGHQVHQA

HQVHQDAGIQDVSMGLDPTSFSVDALMTTRENSAALMTRENQHHTHHPHGLQHPHPHSHSIMTSRESMGA

GVVSRDHLPSVCTTTTTTTAAVAAMMATTGYGHTSSVSRSNGSPPGTMYAPYCTPTAGYIMDHAEYNSRN

HNNTAGHSQWYQEAASPDAAAIYQDTQSPSCQLYRSSPPTPLSSSAAPSPPLYHRYYQDCNTVNTSGNLP

ITLHKY

>XP_006611237.1[Apis dorsata]

MDQTVIVPSTPRGSAVDNSRIAMKNETDSSIHIQDQSTVSNSGPTVVPRANLTIANPLSQDQSLDSLMCS

QSSTELPRKPGARRQEKPPYSYIALIVMAIQSSREKRLTLSEIYSFLQQHFPFFRGAYQGWKNSVRHNLS

LNECFIKLPKGLGRPGKGHYWTIDPSTEYMFEEGSFRRRPRGFRRKCQALKPQYPQYFSGSGPVSVQTAG

YENLAPGAMEYANGYQNQYQNYQEYAMYAPGAAVSADWPYPEATYKTPPIAEVTYKTTEVTYKTGESSVY

RNGEIVAFKSEPGYVARSQEQLTYRAADGFSVKDHQQHHPEPVYKENETMMSYKCPSNPAPTTQPPGQDY

YVGYGLAGVNNTGTNNVNVGMQGIQEQTGNSSPVGNVNSPHSGCQTPVTDNSIKMQCSNSNSNSSGGGLI

DRKPSYFGHPAGSVTLSSLSSLSSLNLSNIGGLSSGLSISNIPGAVSSNIHHTATSTPPTTMYYDPIKYS

MSNV

>XP_006613036.1[Apis dorsata]

MEPRGMMMSNLSCDGCADSDRDSDSSSMIVDPVSLDKNDLSPSQSSSSPIIPSSPVPPTTTSSRNRGGSR

SKKSYSMMSGGNGQQKSNASGQAPSSYGNDKMSSSLIKPPYSYIALITMAILQSPQKKLTLSGICEFIMS

RFPYYHDKFPAWQNSIRHNLSLNDCFIKIPREPGNPGKGNYWTLDPLAEDMFDNGSFLRRRKRYKRPPPH

YVLRDRAIMATFAICGDRGPCPGGGGGQPGALAYPSAAYLSPPPGLPLLDFSPSTLEALKLGGFLEPPPP

LYKPVPITAPPIRQIDPAPTRTTAIPSGHPPAEKKRNFSIDALIGKQAANDQNCGGLLDLSPSEHREIRS

QASAFSPLV

>XP_006617751.1[Apis dorsata]

MESNSYQNHPFSISKIYSCDLSRGCELWSTEETFPTMCPLPNYYSLVPDILNPRSIPLSWDLPIIRYKPQ

PRYEKPPYSYIALIAMAINSSPKQRLTLSGIYRFIMDRFPYYRENRQGWQNSIRHNLSLNDCFVKIPRDK

VVGSDNTEDQAGKGSYWTLDPSASEMFEHGNYRRRRMRRQKGFIQDKQMEQDRTMVSVSSEIFSNLKCKE

KNEQECKTVDTKNTEENTRNSKIIYTEAQGHCVKSMMFTIENILRKSTNKAPV

>XP_006620761.1[Apis dorsata]

MVKMKGESIDVEKRMNRDSPSMKENMHNDDCDVEAELTSLSWLQSLDITSASSLPTPPCSPSPPPVTRQP

RKKLSPLIKAELDLAANADKYRTDPDAKPPFSYATIICLAMRANNNKVSLSNIYAWIRENFLFYKYADPA

WQNSIRHNLSLNKCFVKLPRSKDEPGKGGFWKLDLERMEEGRKSKRRATMSQRIRGSKKLTSGKTTVTND

AQSNGAPSINCSSTLYETPPSSVSPPIPDCLSEIPDSTQVSLSEDDLTGLLIATAGWDENQLDLLDSLLD

TL

>XP_006621636.1[Apis dorsata]

MTSKYGVSISWVLCRVCLRCPSLENLRRLPSVYSNTDKKNTSHNMPPDRPDASDVETGIGLSTQVFNFGD

TVYTMKEKENASVFVETNSTDELSMSEKSIEKNSQSDDDLTSLSWLHQQNLLKGLDISNPTKEIKNENVL

NNNVCDDMADYSENTNSISSLDDGYCPGDSNSKINNTTSHGNNQNYQVSSKNGQKSMQIFQESVKNHYSI

SQNNQTKISLNNNNLPVSNRNKHPTHIPYDPHLHRNSKPPYSFSCLIFMAIEDSPVKALPVKEVYAWILD

HFPYFRNAPTGWKNSVRHNLSLNKCFRKVEKAPNLGKGSLWMVDAQYRPNLIQALSRAPFPPPTVQTLSS

PEKPQKKNISTRLPDPTLFPYLSKRLASSNIIDNTDTEVDSDVDAAAAAMLSFKHGPIILNHNKDRKRKV

PESEMLVPVITRSSSEDHTYSCITSVRQESKYPRKETNTDFDEQRKIAEGADALLNLAGVTTPLNHGRAH

HIQSINSTSKSESSSKLKKRSASNDYSNMSEKRRKHWPKWSEGKRYLKQII

>XP_006623459.1[Apis dorsata]

MEETNMMDSVPVSASGSKTGLQPQQGVPYDLESGFEPQTRARSHTWPLPSPEEYIEGNKKPNVSVRKEGV

EDVESGTPPQHGSLGQGTGLLPVKKNSSRRNAWGNHSYADLITQAITSAPDERLTLSQIYEWMMQNIPYF

REKGESNSSAGWKNSIRHNLSLHSRFMRVQNEGTGKSSWWMINRDAKPGKSRRRAITMETSKFEKRRGRV

RKKIEALKNGGLQADTTTSPSNSVNEGLDLFPDSPLQSGSGFQLSPDFRPRASSNASSCGRLSPITAIPR

KPEWTPTYTLSYSPEQLAGSLAETMKLESYQMYHTTQPSHQHHTGPPPSYYESQYQRSNSLSSGSSSFAL

QPTPQSANQQRCPIHGLQPCACQMNLSPVAGMSPSYQQSEPSPTTLGSNQQQTLQYMMQTQQCQQQQLPQ

RQQQSQTGVTDSSSSQTSTPCDPTPSTMMGQLMGALNNSTLLDDLNINIESLHGGFDCNVEEVIKHELSM

DGTLDFNFQQSVMGTAAIQVSDNNIGQNGAVSQNNVIGTTTTGNATAGVYVSTNAATPAAPPSWVH

>XP_006623756.1[Apis dorsata]

MYLSTYLSKYLSIYLYLSTSPFSPSLILSQRRVRLLGAMSTTYSRTQESDAWALLALKSAPASPTKMQWN

PEAKGAPIARLEGREFEYMVRQRRITIGRNSSKGEVDVNMGHSSFISRRHLEIFYDHPFFFMTCNGKNGV

FVDGVFQRKGAPAFQLPKTCTFRFPSTNIRLVFQSLVDEQEQSNIPVSSPPKHRAPLPPLRINIPDTGYS

SPFPSPTGTISAANSCPASPRAGQGRRNISADLQMVAVYAAAVANDPQNSNMERHDGGQSSNRQISPELG

VESRYRSGSSSGPNGTTAHCSPPKDDSKPPYSYAQLIVQAIASAADKQLTLSGIYSYITKNYPYYRTADK

GWQNSIRHNLSLNRYFIKVPRSQEEPGKGSFWRIDPQSEAKLIEQAFRRRRQRGVPCFRAPFGLSSRSAP

ASPSHVGISGLMTPECLSREASPGPESYPDSTVSSPAGQLTSQSAPGSPGHPYASSSQSSHKGRLMQQIT

VVTNGVSGDTTREDSYIFSDKYVSGNTTEEHSLSPAGQYSPAPVIVQTTYNYSGSFIGPDAGVGVAKRSH

EESDSSPGSPAPLAIVESPEPPEHQQPSTKRQRVHEMDDH

>XP_003690817.1[Apis florea]

MCSNESPPPAKEPLAVGLGNPMTGFLPGLEHYRLQLYHYAMAERLRLAQQLHPQHPGVGHPPSTAPAHLG

MGPGFPAPLPLYPAAAAAAGYPSRLALSMALLHPHHQRIPEEPKPQHSYIGLIAMAILSSPEKKLVLSDI

YQHILEHYPYFRTRGPGWRNSIRHNLSLNDCFVKSGRSANGKGHYWAIHPANLEDFRRGDFRRRKAQRKV

RRHMGLAVDEEPDSPSPPPLPATPPPPATLGPPVASQPMSGIWTPHHHHPHHQQQQQQQQQQQQQQQTFQ

QSQALRQSSSFQPSRKRQFDVASLLAPDDQHQIESDLRSAKSRRFSCSEDELHDLQEADQDEEDVDVDVV

AETNALPSPNTPSASPESKLVSPTTHHWTNQGIQNTGQQLPGLHLQNHLQARNYYVAATSSTGPTV

>XP_003692941.1[Apis florea]

MEETNMMDSVPVSASGSKTGLQPQQGVPYDLESGFEPQTRARSHTWPLPSPEEYIEGNKKPNVSVRKEGV

EDVESGTPPQHGSLGQGTGLLPVKKNSSRRNAWGNHSYADLITQAITSAPDERLTLSQIYEWMMQNIPYF

RXKGESNSSAGWKNSIRHNLSLHSRFMRVQNEGTGKSSWWMINRDAKPGKSRRRAITMETSKFEKRRGRV

RKKIEALKNGGLQADTTTSPSNSVNEGLDLFPDSPLQSGSGFQLSPDFRPRASSNASSCGRLSPITAIPR

KPEWTPTYTLSYSPEQLAGSLAETMKLESYQMYHTTQPSHQHHTGPPPSYYESQYQRSNSLSSGSSSFAL

QPTPQSANQQRCPIHGLQPCACQMNLSPVAGMSPSYQQSEPSPTTLGSNQQQTLQYMMQTQQCQQQQLPQ

RQQQSQTGVTDSSSSQTSTPCDPTPSTMMGQLMGALNNSTLLDDLNINIESLHGGFDCNVEEVIKHELSM

DGTLDFNFQQSVMGTAAIQVSDNNVGQNGAVSQNNVIGTTTTGNATAGVYVSTNAATPAAPPSWVH

>XP_003693150.1[Apis florea]

MDQTVIVPSTPRGSAMKNETESNLHLQDQSATSVSHSNSVVPRANLTMANALSQDQGLDSLMCSQTSGEL

PRKPGARRQEKPPYSYIALIVMAIQSSREKRLTLSEIYSFLQQHFPFFRGAYQGWKNSVRHNLSLNECFI

KLPKGLGRPGKGHYWTIDPSTEYMFEEGSFRRRPRGFRRKCQALKPQYPQYFSGSGPVSVQTAGYENLGA

GAMEYANGYQNQYQNYQEYAMYAGPGAADWPYPEPAYKTPPIAEVTYKTTEVTYKTGESSVYRNGEIVAF

KSEPGYVARSQEQLTYRAADGFSVKDHQQHHPDPVYKENEAMMSYKCPASNPAPTTQPPGQDYYVGYGLA

GVNNTGNNVNVGMQGMPEQGTGNSSPVGGNPVNSPHSGCQTPVTDNSIKMQCSNSNSNSSGGGLIDRKPS

YFGHPAGSVTLSSLSSLSSLNLSNIGGLSSGLSISNIPGAVSSNIHHATTTPSTTMYYDPIKYNMSNV

>XP_003693156.1[Apis florea]

MESNNYQNHPFSISKIYSCDLSRGCELWSTEEAFPTMCPLPNYYSLVPDIINPRSIPLSWDLPIIRYKPQ

PRYEKPPYSYIALIAMAINSSPKQRLTLSGIYRFIMDRFPYYRENRQGWQNSIRHNLSLNDCFVKIPRDK

VVGNDNAEDQAGKGSYWTLDPSASEMFEHGNYRRRRMRRQKGFIQDKQMEQDRTMVSVSSEILSNLKCKE

KNEQEYKTVDTKNAEGNTRNSKIIYTEAQGHCVKSMMFTIENILRKSTNKTPV

>XP_003693368.1[Apis florea]

MPRPSRDTYGDQKPPYSYISLTAMAIWSSRDKMLPLAEIYKFIADRFPYYRKDTRRWQNSLRHNLSFNDC

FIKVPRGPHRPGKGAYWALHPAALSMFENGSLLRRRKRFKLHKPDKELLKSELQALASAMPPPHSESSPV

LSINPNAQTSSLTVANLHRLRDDLLRWELQERRLAMTSGGNSSPSFTTPEAGSSYYLLSPEVRQRLAGTD

EILRGYESNLLQAGSWSFPGFQVSPYVSQLSYFQTDIPESRQICAGNAEAGVPEKTDSRLFLETARGATI

ESADGKVRCPPLEAGISNQTSIQPGQKKKKKPFTIENIIAPDDEQISSNSEDEKRSSGHLLVPRPLYAGF

SFGLPTSKVPYETAT

>XP_003694169.1[Apis florea]

MNYDLDKPRMVSHVIPVAGELQQGNGNLGVTGLGGMQESSHSLKIKQEPFQVSPPSPLPPLHQVSSFVSE

MQNGCIGVTSKELDSSVNLTRGLTSHHTSLNHHHSHHSLHSPNSAVSMHSTGSTHHHLDEKGSSPTGALH

STTNSNPSTPSTPSTPTTVADGSSTATVTGNGNSNNDSASKKPPYSYVALIAMAIQHSAQKRATLSEIYA

YITARFPYFQKNKKGWQNSIRHNLSLNECFVKVPRDGGGERKGNFWTLDPQYVDMFENGNYRRRRRMKRP

YRNAPYKPLFGDPFTPTHVHLGPRNLFGPSPPSYAPSTYTRYDTSAWSLQQSQLPYSHCPSLQPQLQPMQ

SMQIPNMNGYSQLGTSLSNYLDVPGGTTSSSGSMTSGSFGSSFARRHDAAMTQETVPTRCYWPEMVNVKE

EPGTVAVSSTSVGSVGVPSGMMGSTTPSGVSTTGFAPMEFQSRSKCFM

>XP_003694600.1[Apis florea]

MVNMEGSGEQQTVTASPPPRVPLKSSFSIRSILPEACAGTPAPAVTRPSSPEISHVDESEDSSDLDVTGD

GGAETPPLDCSRNAATTVNSSEQKDSKDRQSDDKKKCEKPPYSYNALIMMAIRQSPEKRLTLNGIYEYIM

RHFPYYENNKQGWQNSIRHNLSLNKCFVKVPRHYDDPGKGNYWMLDPSSEDVFIGGTTGKLRRRTTAASR

SRLAAFKRSVVLGGLYPSAYAPPGWPASLYTLPYLHRAAAAYPPATGAYSTPAGYPASLLPGAATSSSTS

LPCKPQPLPATAAPPQHGPFSMERLLQPPTAAGYPAGIAASGIPVSGTPYDFYSTLRSLAAHQHQSTAAV

FAHNQQQSARYHVNQPPLLSQPTSATASPGSSPEPMSPHSPPVTICNSVGVQQPRSLPHSPPQLLLKPIT

VLTGRQS

>XP_003695390.1[Apis florea]

MIGPSSGIGGVSEHELSDNQSRSGKKRIFEDLDIENFCEEIQNGHKRYQYQEIEAPMEMVASNQVINDTA

TLFSPLSAQEAVDECSVGRLEVLLVDGTNCTWTTVSELESQQSIADITASPSTSTSSSENRQEQVIQEVQ

IEETSYPIDCDYWEPVVTVQSSNIQQNKPMMLSGNNQQQQFDDQETGNLSWLLDFKLDPFIEAADEKSTV

SLSKDIHNGNKARMNGRSYGSAYINDAKKNYNENGSLHQESNHNYSSLDNRNFASSRCNGPKKPPFTYTE

LIEHALRERGELTVSAIYQWISEHFPYYKSNDDRWKNSVRHNLSINPHFRKGSKAPHGAGHLWAIANRSG

DSRPRQTINNSIINSSTKQINKNSIEIENLRKNVSQMNSIDEVEAATASITQQSNEEETENMVNSVTLEH

CAEEILSGIKKEVEVQYLVPMMVSNNESTHPNQQTQELHYPVKESDFLNPVSKEVVAEECGLISEGYLVT

DLNPTALGLNMIEPEIITPENLFGEELSFQFYELSSPSQIQSA

>XP_012339603.1[Apis florea]

MPPDRPDTSDVETGIGLSTQVFNFGETVYTMKEKENANVFVETNSTDELSMSEKSIEKNSQSDDDLTSLS

WLHQQNLLKGLDISNPTKEMKNENVLNNNICDDMADYSENTNSISSLDDGYCPGDSNSKINNTTSHGNNQ

NYQVSSKNGQKSMQIFQESVKNHYSISQNNQTKISLNNNNLPVSNRNKHPTHIPYDPHLHRNSKPPYSFS

CLIFMAIEDSPVKALPVKEVYAWILDHFPYFRNAPTGWKNSVRHNLSLNKCFRKVEKAPNLGKGSLWMVD

AQYRPNLIQALSRAPFPPPTVQTLSSPEKPQKKNISTRLPDPTLFPYLSKRLASSNIIDNTDTEVDSDVD

AAAAAMLSFKHGPIILNHNKDRKRKVPESEMLVPVITRSSSEDHTYSCITSVRQESKYPRKEANTDFDEQ

RKIAEGADALLNLAGVTTPLNHGRAHHIQSINSTPKSESSSKLKKRSASNDYSNISEKRRKHWPKWSEGK

RYLKQII

>XP_003692163.2[Apis florea]

MVKMKGESIDVEKRTNRDSPSMKENMHNDDCDVEAELTSLSWLQSLDITSASSLPTPPCSPSPPPVTRQP

RKKLSPLIKAELDLASNAEKYRTDPDAKPPFSYATIICLAMRANNNKVSLSNIYAWIRENFLFYKYADPA

WQNSIRHNLSLNKCFVKLPRSKDEPGKGGFWKLDLERMEEGRKSKRRATMSQRIRGNSKKLTCGKATVTN

DAQSNGAPSINCTLYETPPSSVSPPIPDCLPEIPDSTQVSLSEDDLTGLLIATAGWDENQLDLLDSLLDT

L

>XP_012340462.1[Apis florea]

LSRKAVAAITSAPKLLRHLGSSLVPVQARTNAGEDDGIMDHDTDGDGAINLSTSQRPSAATTPNGDTPSY

GQDQQDNDQATSLFAALKQQQQQQQPRDTVPSSRERENRDRDRLVARNRENNRNEIGGGVTEQSQPTQQQ

QQQQQELTIEYQSNGKLSPAGHAVTAAPMTQQKQPIITQQSQQPSSGAPGPQPSPHQSPQAPQRGSPPNP

SQGPPPGGPPGAPPSQNPSQMMLSPASGIHQMQQLLQQHILSPTQLQSFMQQHSLYLQQQQQQHHQDSSS

EHASNQERFGYFSSLKDHQHQFAELGRKKLEQAIQQLQEQLQLNVIQQTHLLQTADKKKASAPLQQLALQ

QQRLIQQLQITQSQYLLQQGLGLQGHNPSSGLQPGEGLPMWKSDTSDGPESHQNSNVPKSVAGLNGLLNS

TVSSRRSDMNGTTPLDEKPLDVSSNDKVHPLYGHGVCKWPGCEVICEDYQAFLKHLNTEHTLDDRSTAQA

RVQMQVVSQLEIQLQKERDRLTAMMHHLHVAKQMASPEPPKSSESSTGSSIPKLNLSTALMSQPPPNFGV

SQVSPVSMSALVSAVRSPAGGQLPPSAGAPMPPIPNMSNMSGMPPLPNMPGSMPTMPTMPSMAGPIRRRI

SDKSALSLAGGLYDEGTVRRRVAVDRSGIDINEEIQRNREFYKNADVRPPFTYASLIRQSIIESPDKQLT

LNEIYNWFQNTFCYFRRNAATWKNAIRTNLSLHKCFVRYEDDFGSFWMVDDAEFVKRRHLSRGRPRKYDP

TPSPTPPHLSAQ

>XP_012341189.1[Apis florea]

MHTLFSEQNAYYRHATAVPVGMGTPSYPGVGAAPGYYEQYRYGGYATAAGYPVSGITQQHIHHPGKDMVK

PPYSYIALIAMAIQNAPDKKITLNGIYQFIMERFPYYRENKQGWQNSIRHNLSLNECFVKVPRDDKKPGK

GSYWSLDPDSYNMFDNGSYLRRRRRFKKKDALKEKEEALKRQGLVVAEKQRSQEETKPSNIVIPPPSDTS

AKKLGTLETTLCKPKREPVNDTGSHCMAVQAKYGLHSPIQDTKTAVTTTTAVAVPGQSVIQTALGHQVHQ

AHQVHQDAGIQDVSSMGLDPTSFSVDALMTSRENSAALMTRENQHHTHHPHGLQHPHPHSHSIMTSRESM

GAGVVSRDHLPSVCTATTTTTAAVAAMMATTGYGHTSSVSRSNGSPPGTMYAPYCTPTPGYIMDHAEYNS

RNHNNTAGHSQWYQEAASPDAAAIYQDTQSPSCQLYRSSPPTPLSSSAAPSPPLYHRYYQDCNTVNTSGN

LPITLHKY

>XP_012341202.1[Apis florea]

MTMLQSQKLYSDAGSLGGAMTSAAMSTMGSMAPTYSSINSVGCMPMGMSMGVGVGPSCSPQGAGGFNMST

MSSAMGMAASMGGGGMGGYGSASMGGGGACMSAVGYGPLTPGGGAGVTRDPLSLAEPDSPNSALQRARTD

KSYRRSYTHAKPPYSYISLITMAIQNAPTKMLTLSEIYQFIMDLFPFYRQNQQRWQNSIRHSLSFNDCFV

KVARTPDKPGKGSFWTLHPESGNMFENGCYLRRQKRFKDEKKELTRQNNKHQQHHTGAAAAAAAVAVAAA

AGHNSPTSHELAHAAKKSAASSLHHGPQQGQDDKDLHSLVTSHHHHHATLHQHHATLKSDATDIGGLLGP

DLGAAHDELTAMVSRSLHPHLISEPAAALHHGMAGSLKQEPPYTAASHXFSITRLLPGATAATSPGAQDI

KPPEMKMYEQLHQSYANFGSPHHPHAHSAPPSHHHHNGMHAAASNAATPMHNMTNHHHQEYYQSPLYHHA

TSVASSSAPPPSTVATAAPGL

>XP_012342768.1[Apis florea]

MLDTESRHGTELVQGPTRDPWLTMDYYLGTDSLSLQEMLDVDIKCEIEGVIGGHTELGFNFTDMSGLEMD

DDPIGCQNDLSGWFGSTSLLNGNSNSSNSNFNLDLSGSDAASIMVNPNSVMPHMALRSPPPNNNRRHFSF

SPKKDMKEKIDIEEEIENEGSNYTENENENENENENENENENENENENENENENENENENENENENEIEN

ENENENENENENEQENEIENDTETEQYENDITETEEDSEDEQEVSKSVTPSKSKTISISAAKMTSPQYTK

AQTTPKINGMSGIRIQNFKVLQSSNQMPQHVRKQIYNNNVHINPGSTTITTVKREKEFDLSDYDDKPYPK

PAYSYSCLIAMALKNSQTGSLPVSEIYNFMCEHFPYFKTAPNGWKNSVRHNLSLNKCFEKIEKPAGNGNQ

RKGCLWAINPAKVAKMDEEVQKWSRKDPLAIKKAMIYPDHLELLERGEMKYAGSGDVSEETESSGDEAVE

ESTTYEESIHGHITANSVTDSYDESSQDCDIDIHEHLYDEIDIEDNKEALHMHLNISKQESFEYELNSGT

KRQKTLTGAIQGNYVYQPVTTSRRKTPLLVRAGAGNSSFLKID

>XP_012347723.1[Apis florea]

MEPRGMMMSNLSCDGCADSDRDSDSSSMIVDPVSLDKNDLSPSQSSSSPVIPSSPVPPATASSRNRGGSR

SKKGYSMMSGXNGQQKGNAAGQASTYGNEKMSSSLIKPPYSYIALITMAILQSPQKKLTLSGICEFIMSR

FPYYHDKFPAWQNSIRHNLSLNDCFIKIPREPGNPGKGNYWTLDPLAEDMFDNGSFLRRRKRYKRPPPHY

VLRDRAIMATFAICGDRGPCPGGGGGHPGALAYPSAAYLSPPPGLPLLDFSPSTLEALKLGGFLEPPPPL

YKPVPITAPPIRQIDPAPTRTTAIPSGHPPAEKKRNFSIDALIGKQAANDQNCGGLLDLSPSEHREIRSQ

ASAFSPLV

>XP_012348616.1[Apis florea]

MSTTYSRTQESDAWALLALKSAPASPTKMQWNPEAKGAPIARLEGREFEYMVRQRRITIGRNSSKGEVDV

NMGHSSFISRRHLEIFYDHPFFXMTCNGKNGVFVDGVFQRKGAPAFQLPKTCTFRFPSTNIRLVFQSLVD

EQEQSNISVSSPPKHRAPLPPLRINIPDTGYSSPFPSPTGTISAANSCPASPRAGQGRRNISADLQMVAV

YAAAVANDPQNSNMERHDGGQSSNRQISPELGVESRYRSGSNSGPNGTTAHCSPPKDDSKPPYSYAQLIV

QAIASAADKQLTLSGIYSYITKNYPYYRTADKGWQNSIRHNLSLNRYFIKVPRSQEEPGKGSFWRIDPQS

EAKLIEQAFRRRRQRGVPCFRAPFGLSSRSAPASPSHVGISGLMTPECLSREASPGPESYPDSTVSSPAG

QLTSQSAPGSPGHPYASSSQSSHKGRLMQQITVVTNGVSGDTTREDKYVSGNTTEEHSLSPAGQYSPAPV

IVQTTYNYSGSFIGPDAGVGVAKRSHEESDSSPGSPAPLAIVESPEPPEHQQPSTKRQRVHEMDDH

>XP_003484718.1[Bombus impatiens]

MDQTVIVPSTPRGSNVDNARVTMKNESDSNLHIQDQSTVAHSNSTSVVPRTNLSMSNSLNQDQSLDPLMC

NPASTELPRKPGARRQEKPPYSYIALIVMAIQASPGKRLTLSEIYSFLQQHFPFFRGAYQGWKNSVRHNL

SLNECFIKLPKGLGRPGKGHYWTIDPSTEYMFEEGSFRRRPRGFRRKCQALKPQYPQYFSASGPVGVQTP

GYENLTPGAMEYANGYQNQYQNYQEYAMYAAPGAAVSADWSYPEATYKTPPISEVTYKTTEVTYKTGEPS

VYRNGEIVAFKSEPGYAARSQEQLTYRATDGFSVKDHQQHHPETVYKENETMMTYKCPSNPAPTTQAPGQ

DYYVGYGLAGVNNTGSNVNVAMQGIPEQTGNNSPVGNVSSPHSGCQTPVTDNGIKMQCSNSNSNSSGGGL

IDRKPSYFGHPAGSVTLSSLSSLSSLNLSNIGSLSSGLSISNIPGTVSSNIHHTATTPPTYYDQIKYSM

>XP_003487173.1[Bombus impatiens]

MCSNESPPPAKEPLAVGLGNPMTGFLPGLEHYRLQLYHYAMAERLRLAQQLHPQHPGVGHPPSSAPTHLG

MGPGFPAPLPLYPAAAAAAGYPSRLALSMALLHPHHQRIPEEPKPQHSYIGLIAMAILSSPEKKLVLSDI

YQHILEHYPYFRTRGPGWRNSIRHNLSLNDCFVKSGRSANGKGHYWAIHPANLEDFRRGDFRRRKAQRKV

RRHMGLAVDEEPDSPSPPPLPATPPPPATLGPPVASQPMPGIWTPHHHHPHHQQQQQQQQTFQSQTLRQS

STFQPSRKRQFDVASLLAPDDQHQIESDLRPQKARRFSCSEDELHDLQEPDQDEEDVDVDVVAETNTLPS

PNTPSASPEAKVISTAGHWSNQGIQSGQISGLHIQSHLQARNYYVPATSSNGSNV

>XP_003487622.1[Bombus impatiens]

MTQQKQPIITQQSQQPSSGAPGPQPSPHQSPQAPQRGSPPNPSQGPPPGGPPGAPPSQNPPQMMLSPASG

IHQMQQLLQQHILSPTQLQSFMQQHSLYLQQQQQQHHQDSSSEHASNQERFGYFSSLKDHQHQFAELGRK

KLEQAIQQLQEQLQLNVIQQTHLLQTADKKKASAPLQQLALQQQRLIQQLQITQSQYLLQQGLGLQGHNP

SSGLQPGEGLPMWKSDTSDGPETHQNSNVPKSVAGLNVSSRRSEMNGTTPLDEKPLDVSSNDKVHPLYGH

GVCKWPGCEVICEDYQAFLKHLNTEHTLDDRSTAQARVQMQVVSQLEIQLQKERDRLTAMMHHLHVAKQM

ASPEPPKSSESSTGSSIPKLNLSTALMSQPPPNFGVSQVSPVSMSALVSAVRSPAGGQLPPSAGGAPMPP

IPNMSNMSGMPPLPNMPGSMPTMPTMPSMAGPIRRRISDKSALSLAGGLYDEGTVRRRVAVDRSGIDINE

EIQRNREFYKNADVRPPFTYASLIRQSIIESPEKQLTLNEIYNWFQNTFCYFRRNAATWKNAVRHNLSLH

KCFMRVENVKGAVWTVDEVEFYKRRPQRACSTTGGVPSKSPTLTHSPTMYGDALNANLQAALGDSNMGFL

NNSMCTSTTTSPDKEHVLAHNDLMSPLDEPAVHIKQEGQSPEGGKLTRLIKRELVDAPTEQEGEEDQVDE

REYPESHGHDSGQDEDMAEDLSMAPDIMTPEDQIEA

>XP_003489326.1[Bombus impatiens]

MIGPSSGTGGVPEHELSDNQSRVGKKRIFEDLDIESFCEEVQNGHKRYQYQEIEAPMEVVASNQVIGDTA

ALFSPLSAQEAVDECPVARLEVLLVDGTNCTWTTVSELESQQNIVDIAASPTTSSSSSEHRQEQVIQEIQ

IEETSYPIGSDYWEPVVTVPSPNMQQGKTDASSSSHQQQQFDDQETGNLSWLLDFKLDPFIEAADEKSTV

PSPKDVHSGNKTRVNGRSYGSTYVNDVKRSYNENGSSHQESNHSYSSLDNRNFASSRCNGPKKPPFTYTE

LIEHALRERGELTVSAIYQWISEHFPYYKSNDDRWKNSVRHNLSINPHFRKGSKAPHGAGHLWAIANRSG

DPRPRQSINNFNVNPSIKQMSKNTLEVENLRKNISEMNSIDEVEAATASITQQSSEEEADNVVNSVTLEH

CAEEILSGIKKEVEVQYLVPMIVSNNEHESTQSNQQTQELHYPVKESDFLNPVSKEVVAEECGLISEGYL

VTDLNPTTLGLNMVEPEIITPENLFGEELSFQFYELSSPSQIQSA

>XP_003492833.1[Bombus impatiens]

MPRPSRDTYGDQKPPYSYISLTAMAIWSSRDKMLPLAEIYKFIADRFPYYRKDTRRWQNSLRHNLSFNDC

FIKVPRGPHRPGKGAYWALHPAALSMFENGSLLRRRKRFKLHKPDKELLKSELQALASAMPPPHSESSPV

LSINPNAQTSSLTVANLHRLRDDLLRWELQERRLTMASGGNSSPGFTTPETSSSYYLLSPEVRQRLAGTD

EILRGYENNLLQTGSWSFPGFQVSPYVSQLSYFQTDIPESRQICAGTTETSVPEKTDSRVFLETTRGSTV

ESTDSKVRCPVLEAGISSQTSIQPEQKKKKKPFTIENIIAPDDEQISSNSEDEKRSSGHLLVPRPLYAGF

SFGLTANKVPYETAT

>XP_003492949.1[Bombus impatiens]

MLDTESRHGTGLVQGPTRDPWLTMDYYLGTDSLSLQEMLDVDIKCEIEGVIGGHTELGFNFTDMSGLEMD

DDPIGCQNDLSGWFGSTSLLNGNSNSSSSNFNLDLNGSDAASIMVNPNSVMPHIAIRSPTPNNGRRHFSF

SSKKDIREKIDVEEEAENEGSSYTENENENENDNENENENENDNDNDNENEQENEIENDTETEEQYENDI

TETEEDSEDEQEISKSVTPSKSKTIAISVARTTSPQYPKAQSTPKMNGMAGIRIQNFKVLQSPNQTHQHV

RKQIYNNNVHINNSGATSVSTVKREKEFDLSDYDDKPYPKPAYSYSCLIAMALKNSQTGSLPVSEIYNFM

CEHFPYFKTAPNGWKNSVRHNLSLNKCFEKIEKPAGNGNQRKGCLWAINPAKVAKMDEEVQKWSRKDPLA

IKKAMIYPDHLELLERGEMKYAGSGDVSEETESSGDEAVEENTTYEESIHGHIAANSVTDSYDESSQDCD

VDIQEHLYDEIDIEDNKDALHMHLNISKQEPFEYELSSGTKKQKTLTGAIQGNYVYQPVTTSRRKTPLFL

RAGTGSSSFLKID

>XP_012246619.1[Bombus impatiens]

MTMLQSQKLYGDAGSLGGAMTSAAMSTMGSMAPTYSSINSVGCMPMGMSMGVGVGPSCSPQGAGGFNMST

MSSAMGMASMGGGAMGGYGGASMGGGGACMSAVGYGPLTPGGGAGVTRDPLSLTEPDSPNSALQRARTDK

SYRRSYTHAKPPYSYISLITMAIQNAPSKMLTLSEIYQFIMDLFPFYRQNQQRWQNSIRHSLSFNDCFVK

VARTPDKPGKGSFWTLHPESGNMFENGCYLRRQKRFKDEKKELTRQNNKHQQHHTGAAAAAAAAAAAAAA

AAGHNSPTSHELAHAAKKTTSSLHHGPQQQDDKDLHSLVSSHHHHHAASLHQHHASLKSDTTDIGGLLGP

ELGTAHDELTAMVSRSLHPHLISEPTALHHGMTGSLKQEPPYTAASHPFSITRLLPGATAGTSPGAQDIK

PPEMKMYEQLHQSYANFGSPHHPHAHSAPPSHHHHNGMHTGSNAAGPMHNMTNHHHQEYYQSPLYHHATS

VASSSAPPPSSVATAAPGL

>XP_012236317.1[Bombus impatiens]

MHTLFSEQNAYYRHATAVPVGMGTPSYPGVGAAPGYYEQYRYGGYATAAGYPVSGITQQHIHHPGKDMVK

PPYSYIALIAMAIQNAPDKKITLNGIYQFIMERFPYYRENKQGWQNSIRHNLSLNECFVKVPRDDKKPGK

GSYWSLDPDSYNMFDNGSYLRRRRRFKKKDALKEKEEALKRQGLVPEKQRNQDETKPSNIVIAPPSDAST

KKLGTLDTTLCKPKREPVNDAGSHCMAVQAKYGLHSPIQDTKTAVTTTSAVAIAGQSVIQTALGHQVHQA

HQVHQDSIQDVSMGLDPTSFSVDALMTTRENSAALMTRDNPHHTHHPHGLQHTHPHSHSIMTSRESMGSG

VVSRDHLPSVCATTTTTTAAVAAMMATTGYGHSSTVSRSNGSPPGTMYAPYCTPTAGYIMDHTEYNSRNH

NNTAGHSQWYQEAASPDAAAIYQDTQSPSCQLYRSSPPTPLSSSAAPSPPLYHRYYQDCNTVNTSGNLPI

TLHKY

>XP_012237608.1[Bombus impatiens]

MEPNSYQNHPCSLSKIYNCDLRRNYEFWSTEETFPTMCPLPSCYNLVPDVLTTRSVPLSWDLPLLRYKPQ

PRYEKPPYSYIALIAMAINSSPKQRLTLSGIYRFIMDRFPYYRENRQGWQNSIRHNLSLNDCFVKIPRDK

VIGNDNGEDQAGKGSYWTLDPSASEMFEHGNYRRRRMRRQRGLTQDKQEQHHTVVSVSPEIISNLTRKEK

DEQRCETIEKNAEQAQGHCVKSMMFTIENILRKSTNESPI

>XP_012249348.1[Bombus impatiens]

MVNMEGSGEQETVTASPSPRVVLKSSFSIRSILPEACAGTPAPTVRRSASPEISHVDDSEDSSDLDVTGD

GGTETPPLDCSRNAVNSAANSSEQKDSKDRQTDDKKKCEKPPYSYNALIMMAIRQSPEKRLTLNGIYEYI

MRHFPYYENNKQGWQNSIRHNLSLNKCFVKVPRHYDDPGKGNYWMLDPSSEDVFIGGTTGKLRRRTTAAS

RSRLAAFKRSVVLGGLYPSAYAPPGWPASLYTLPYLHRAAGYPPATGAYSTPAGYPRLLQPPTAAGYPTG

IATSAIPVSGSPYDFYSTLRSLAAHQQHQSSAAVFAHNQQPARYHVNQPPLLITVCNTAVVQQPRSLPHS

PPQLLLKPITVLTGRQS

>XP_012236248.1[Bombus impatiens]

MNYDLDKSRMVSHVIPVAGELQQGNGNLGVAGLGGMQESSHSLKIKQEPFQVSPPSPLPPLHQVSSFVSE

MQNGCIGVSKELDSTVSLARGLTSHHASLTHHHSHHSLHSPNSAVSMHSTGSTHHHLDEKGSSPTGALHS

TTNSNPSTPSTPSTPTTAADGSSTATGTGNTSGSNSDSASKKPPYSYVALIAMAIQHSAQKRATLSEIYA

YITARFPYFQKNKKGWQNSIRHNLSLNECFVKVPRDGGGERKGNFWTLDPQYADMFENGNYRRRRRMKRP

YRNAPYHKPLFGDPFSPTHVHLGPRNLFGHSPPSYAPSTYPRYDTSAWSLQQSQLSYSHCQSLQPQLQPM

QSMQIPTMNGYSQLGTSLTFQGNYLDVPGGTTSSPGSMGSGSFGSSFARRHDAAMTQETVPARCYWPEMV

NVKEEPGTVAVSSTSVGGVGVPSGMMGSTTPTGVSTTGFAPMEFQSRSKCFM

>XP_012238217.1[Bombus impatiens]

MMDSVPVSASGSKTGLQPQQGVPYDLESGFEPQTRARSHTWPLPRPEEYIEGNVVPSVAASKEGVEGVES

GTPPQHGSLGQGAGLLPVKKNSSRRNAWGNHSYADLITQAITSAPDERLTLSQIYEWMMQNIPYFREKGE

SNSSAGWKNSIRHNLSLHSRFMRVQNEGTGKSSWWMINRDAKPGKSRRRAITMETSKFEKRRGRVRKKIE

ALRNGGLQTDATPSPSNSVNEGLDLFPDSPLQPGSGFQLSPDFRPRASSNASSCGRLSPIPAIPGKPEWT

PTYTPSYSPEQLAGSLAETMKLESYQIYQTSPSSHQQQTGPPPSYYETQYQRSNSLSTGSSSFALQPTPQ

SGNQQRCPLHGLQPCACQMNLSPVAGMSPSYQQSEPSPTALGSNQQQTLQYMMQTQQCQQQQQQQQQQQQ

QQSQTGATGSSPPQTPTPCGSTPSTMMGQLMGALNNSTLLDDLNINIESLHGGFDCNVEEVIKHELSMDG

TLDFNFQQGVMGTAAIQVSDSNVGQNGTVSQNNVVGTTAGNAPAGVYVSTNAATPAASPSWVH

>XP_012238673.1[Bombus impatiens]

MPPDRPDASGVETGIGLSSTKVFNFGETVYTMKEKENASAFVETNSTDELSMSDKSIEKNSQSDDDLTSL

SWLHQQNLLKGLDISNPTKDMKNENVLNNNVCDDMADFSENTNSVSSLDEGYCPADNNSKINNTTPHGNS

QNYQHSNKNSQKSMQIFQESVKNHYSTSQNNQTKISLNNNNLPVSNRNKHPTHIPYDPHLHRNSKPPYSF

SCLIFMAIEDSPVKALPVKEVYAWILDHFPYFRNAPTGWKNSVRHNLSLNKCFRKVEKAPNLGKGSLWMV

DAQYRPNLIQALSRAPFPPPTAQTLSSPEKPQKKNTSTRLPDPILFPYLSKRLASSNITDNTDTEVDSDV

DAAAAAMLSFKHGPIILNHNKDRKRKVPESEVLVPVITRSSSEDHTYSCITSVKQESKYPRKETNPDFDE

QRKIAEGADALLNLAGVTTPLNHNRTHHVQGINSTPKSDGTSKLKKRSASNDYSNTPEKRRKHWPKWSEG

KRYLKQII

>XP_012243475.1[Bombus impatiens]

MSTTYSRTQESDAWALLALKSAPASPTKMQWNPEAKGAPIARLEGREFEYMVRQHSITIGRNSSKGEVDV

NMGHSSFISRRHLEIYYDHPYFFMVCNGKNGVFVDGIFQRKSAAEFRLPKTCTLRFPSTNIRLVFQSLVD

EQEESNVCVRSPPKHRAPLPPLRINIPDAGYSSPFPSPTGTISAANSCPASPRAGQGRRNISADLQMVAV

YAAAVANDSQNSNMERHEGGQSSNRQISPGTTANCSPPKDDSKPPYSYAQLIVQAIASAADKQLTLSGIY

SYITKNYPYYRTADKGWQNSIRHNLSLNRYFIKVPRSQEEPGKGSFWRIDPQSEAKLIEQAFRQRRQRGV

PCFRAPFGLSSRSAPASPSHVGISGLMTPECLSREVSPGPESYPDSTVSSPAGQLTSQSAPGSPGHPYAS

SSQSSHKGRLMQQITVVTNGVSGDTTREDKYVVSGNTTEEHSLSPAGQYSPAPVIVQTTYNYSGSFIGPD

AGVGVAKRSHEESDSSPGSPAPLAIVESPEPPEHQPPSTKRQRVHDMDDH

>XP_012247232.1[Bombus impatiens]

MVKMKAESIDTKKPTNRGVSIMKENMHDDDYGVEAELTSLSWLQSLDITSASSLPTPPCSPSPPPVTRQP

RKKLSPLIKAELDLAANAEKYRTDPDAKPPFSYATIICLAMRANNNKVSLSNIYAWIRENFLFYKYADPA

WQNSIRHNLSLNKCFVKLPRSKDEPGKGGFWKLDLERMEEGRKSKRRATMSQRIRGSKKLTAAKTMVTNN

IQSNGAPSINCPSTLYETPPSSVSPPIPDCLSEIPDSTQVSLSEDDLTGLLIATAGWDENQLDLLDSLLD

TL

>XP_012248814.1[Bombus impatiens]

MEPRGMMMSNLSCDGCADSDRDSDSSSMIVDPVSLDKNDLSPSQSSSSPIISSSPVPPTTASSRNRGGSR

SKKNYSMMSANSQQKNNSSGQASSYSNDKMSSSLIKPPYSYIALITMAILQSPQKKLTLSGICEFIMSRF

PYYHDKFPAWQNSIRHNLSLNDCFIKIPREPGNPGKGNYWTLDPLAEDMFDNGSFLRRRKRYKRPPPHYV

LRDRAIMATFAICGDRGPCPGGGGGHPGALTYPSAAYLSPPPGLPLLDFSPSTLEALKLGGFLEPPPPLY

KPVPITAPPIRQIDPAPTRTTTIPSSHPPADKKRNFSIDALIGKQAANDQNCGGXLDLSPSEHREIRSQA

SAFSPLV

>XP_012167125.1[Bombus terrestris]

MEPRGMMMSNLSCDGCADSDRDSDSSSMIVDPVSLDKNDLSPSQSSSSPIIPSSPVPPTTASSRNRGGSR

SKKNYSMMSANSQQKNNSSGQASSYSNDKMSSSLIKPPYSYIALITMAILQSPQKKLTLSGICEFIMSRF

PYYHDKFPAWQNSIRHNLSLNDCFIKIPREPGNPGKGNYWTLDPLAEDMFDNGSFLRRRKRYKRPPPHYV

LRDRAIMATFAICGDRGPCPGGGGGHPGALTYPSAAYLSPPPGLPLLDFSPSTLEALKLGGFLEPPPPLY

KPVPITAPPIRQIDPAPTRTTTIPSSHPPADKKRNFSIDALIGKQAANDQNCGGLLDLSPSEHREIRSQA

SAFSPLV

>XP_012165509.1[Bombus terrestris]

MHTLFSEQNAYYRHATAVPVGMGTPSYPGVGAAPGYYEQYRYGGYATAAGYPVSGITQQHIHHPGKDMVK

PPYSYIALIAMAIQNAPDKKITLNGIYQFIMERFPYYRENKQGWQNSIRHNLSLNECFVKVPRDDKKPGK

GSYWSLDPDSYNMFDNGSYLRRRRRFKKKDALKEKEEALKRQGLVPEKQRNQDETKPSNIVIAPPSDAST

KKLGTLETTLCKPKREPVNDAGSHCMAVQAKYGLHSPIQDTKTAVTTTSAVAVAGQSVIQTALGHQVHQA

HQVHQDSIQDVSMGLDPTSFSVDALMTTRENSAALMTRDNPHHTHHPHGLQHPHPHAHSIMTSRESMGSG

VVSRDHLPSVCATTTTTTAAVAAMMATTGYGHNSTVSRSNGSPPGTMYAPYCTPTAGYIMDHAEYNSRNH

NNSAGHSQWYQEAASPDAAAIYQDTQSPSCQLYRSSPPTPLSSSAAPSPPLYHRYYQDCNTVNTSGNLPI

TLHKY

>XP_012167474.1[Bombus terrestris]

MTMLQSQKLYSDAGSLGGAMTSAAMSTMGSMAPTYSSINSVGCMPMGMSMGVGVGPSCSPQGAGGFNMST

MSSAMGMASMGGGAMGGYGSASMGGGGACMSAVGYGPLTPGGGAGVTRDPLSLTEPDSPNSALQRARTDK

SYRRSYTHAKPPYSYISLITMAIQNAPSKMLTLSEIYQFIMDLFPFYRQNQQRWQNSIRHSLSFNDCFVK

VARTPDKPGKGSFWTLHPESGNMFENGCYLRRQKRFKDEKKELTRQNNKHQQHHTGAAAAAAAAAAAAAA

GHNSPTSHELAHAAKKTTSSLHHGPQQQDDKDLHSLVSHHHHHSAGLHQHHASLKSDATDIGGLLGPELG

AAHDELTAMVSRSLHPHLISEPTALHHGMAGSLKQEPPYTAASHPFSITRLLPGATAGTSPGAQDIKPPE

MKMYEQLHQSYANFGSPHHPHAHSAPPTHHHHNGMHTGSNAAGPMHNMTNHHHQEYYQSPLYHHATSVAS

SSAPPPSSVATAAPGL

>XP_003396602.1[Bombus terrestris]

MDQTVIVPSTPRGSNVDNARVTMKNESDSNLHIQDQSTVAHSNSTSVVPRTNLSMSNSLNQDQSLDPLMC

NPASTELPRKPGARRQEKPPYSYIALIVMAIQASPGKRLTLSEIYSFLQQHFPFFRGAYQGWKNSVRHNL

SLNECFIKLPKGLGRPGKGHYWTIDPSTEYMFEEGSFRRRPRGFRRKCQALKPQYPQYFSASGPVGVQTP

GYENLTPGAMEYANGYQNQYQNYQEYAMYAAPGAAVSADWSYPEATYKTPPISEVTYKTTEVTYKTGEPS

VYRNGEIVAFKSEPGYAARSQEQLTYRATDGFSVKDHQQHHPETVYKENETMMTYKCPSNPAPTTQAPGQ

DYYVGYGLAGVNNTGSNVNVAMQGIPEQTGNNSPVGNVSSPHSGCQTPVTDNGIKMQCSNSNSNSSGGGL

IDRKPSYFGHPAGSVTLSSLSSLSSLNLSNIGSLSSGLSISNIPGTVSSNIHHTATTPPTYYDQIKYSM

>XP_003403222.1[Bombus terrestris]

MIGPSSGTGGVPEHELSDNQSRVGKKRIFEDLDIESFCEEVQNGHKRYQYQEIEAPMEVVASNQVIGDTA

ALFSPLSAQEAVDECPVARLEVLLVDGTNCTWTTVSELESQQNMVDIAASPTTSSSSSEHRQEQVIQEIQ

IEETSYPIGSDYWEPVVTVPSPSMQQGKTDVSSSSQQQQQFDDQETGNLSWLLDFKLDPFIEAADEKSTV

PSPKDVHSGNKTRVNGRSYGSTYVNDVKRSYNENGSSHQESNHSYSSLDNRNFASSRCNGPKKPPFTYTE

LIEHALRERGELTVSAIYQWISEHFPYYKSNDDRWKNSVRHNLSINPHFRKGSKAPHGAGHLWAIANRSG

DPRPRQSINNFNANSSIKQMSKNTVEVENLRKNISEMNPIDEVEAATASITQQSSEEESDNVVNSVTLEH

CAEEILSGIKKEVEVQYLVPMIVSNNEHESTQPNQQTQELHYPVKESDFLNPVSKEVVAEECGLISEGYL

VTDLNPTTLGLNMVEPEIITPENLFGEELSFQFYELSSPSQIQSA

>XP_003401813.1[Bombus terrestris]

MPRPSRDTYGDQKPPYSYISLTAMAIWSSRDKMLPLAEIYKFIADRFPYYRKDTRRWQNSLRHNLSFNDC

FIKVPRGPHRPGKGAYWALHPAALSMFENGSLLRRRKRFKLHKPDKELLKSELQALASAMPPPHSESSPV

LSINPNAQTSSLTVANLHRLRDDLLRWELQERRLTMASGGNSSPGFTTPETSSSYYLLSPEVRQRLAGTD

EILRGYENNLLQTGSWSFPGFQVSPYVSQLSYFQTDIPESRQICAGTTETSVPEKTDSRVFLETTRGSTV

ESTDSKVRCPVLEAGISSQTSIQPEQKKKKKPFTIENIIAPDDEQISSNSEDEKRSSGHLLVPRPLYAGF

SFGLTANKVPYETAT

>XP_003394823.1[Bombus terrestris]

MVNMEGSGEQQTVTASPSPRVVLKSSFSIRSILPEACAGTPAPTVRRSASPEISRVDDSEDSSDLDVTGD

GGAETPPLDCSRNSVNSANSPEQKDSKDRQADDKKKCEKPPYSYNALIMMAIRQSPEKRLTLNGIYEYIM

RHFPYYENNKQGWQNSIRHNLSLNKCFVKVPRHYDDPGKGNYWMLDPSSEDVFIGGTTGKLRRRTTAASR

SRLAAFKRSVVLGGLYPSAYAPPGWPASLYTLPYLHRAAGYPPATGAYSTPAGYPASLLPGAATSSTANL

PCKPQPLPATAASPQHGPFSMERLLQPPTAAGYPTGIATSAIPVSGSPYDFYSTLRSLAAHQQHQSTAAV

FAHNQQPARYHVNQPPLLGQPTSATASPGSSPEPMSPNSPPITVCNTAVVQQPRSLPHSPPQLLLKPITV

LTGRQS

>XP_003396763.1[Bombus terrestris]

MEPNSCQNHACSLSKIYNYNLRRSYEFWSTEETFPTMCPLPSCYNLVPDVLTTRSVPLSWDLPLLRYKPQ

PRYEKPPYSYIALIAMAINSSPKQRLTLSGIYRFIMDRFPYYRENRQGWQNSIRHNLSLNDCFVKIPRDK

VIGNDNGEDQAGKGSYWTLDPSASEMFEHGNYRRRRMRRQRGLTQDKQEQHHTVVSVSPEIISNLTRKEK

DEQRCETIEKNVERAQGHCVKSMMFTIENILRKSTNESPI

>XP_012170688.1[Bombus terrestris]

MLEPRWRPVQGHIGENPFDNGSWGKEQFQPSTVPWQLNPRGHARPSDDGIMDHDTDGDGAINLSTSQRPS

AATTPNGDTPSYGQDQQDNDQATSLFAALKQQQQQPRDSIPSSRERENRERDRLSVRSRENNRNEIGGGV

TEQSQQPQQPQQQQQQQELTIEYQSNGKLSPAGHTVTAAPMTQQKQPIITQQSQQPSSGAPGPQPSPHQS

PQAPQRGSPPNPSQGPPPGGPPGAPPSQNPPQMMLSPASGIHQMQQLLQQHILSPTQLQSFMQQHSLYLQ

QQQQQHHQDSSSEHASNQERFGYFSSLKDHQHQFAELGRKKLEQAIQQLQEQLQLNVIQQTHLLQTADKK

KASAPLQQLALQQQRLIQQLQITQSQYLLQQGLGLQGHNPSSGLQPGEGLPMWKSDTSDGPETHQNSNVP

KSVAGLNGLLNSTVSSRRSEMNGTTPLDEKPLDVSSNDKVHPLYGHGVCKWPGCEVICEDYQAFLKHLNT

EHTLDDRSTAQARVQMQVVSQLEIQLQKERDRLTAMMHHLHVAKQMASPEPPKSSESSTGSSIPKLNLST

ALMSQPPPNFGVSQVSPVSMSALVSAVRSPAGGQLPPSAGGAPMPPIPNMSNMSGMPPLPNMPGSMPTMP

TMPSMAGPIRRRISDKSALSLAGGLYDEGTVRRRVAVDRSGIDINEEIQRNREFYKNADVRPPFTYASLI

RQSIIESPEKQLTLNEIYNWFQNTFCYFRRNAATWKNAIRTNLSLHKCFVRYEDDFGSFWMVDDAEFVKR

RHLSRGRPRKYDPTPSPTPPHLSAQGVPSKSPTLTHSPTMYGDALNANLQYFQAALGDSNMGFLNNSMCT

STTTSPDKEHVLAHNDLMSPLDEPAVHIKQEGQSPEGGKLTRLIKRELVDAPTEQEGEEDQVDEREYPES

HGHDSGQDEDMAEDLSMAPDIMTPEDQIEA

>XP_003401894.1[Bombus terrestris]

MLDTESRHGTGLVQGPTRDPWLTMDYYLGTDSLSLQEMLDVDIKCEIEGVIGGHTELGFNFTDMSGLEMD

DDPIGCQNDLSGWFGSTSLLNGNSNSSNSNFNLDLSGSDAASIMVNPNSVMPHIAIRSPTPNNGRRHFSF

SSKKDIREKIDVEEETENEGSSYTENENENENENENENENENENENENENDNDNENEQENEIENDTETEE

QYENDITETEEDSEDEQEISKSVTPSKSKTIAISVARTTSPQYPKAQTTPKMNGMAGIRIQNFKVLQSPN

QTHQHVRKQIYNNNVHINNSGASSVSTVKREKEFDLSDYDDKPYPKPAYSYSCLIAMALKNSQTGSLPVS

EIYNFMCEHFPYFKTAPNGWKNSVRHNLSLNKCFEKIEKPAGNGNQRKGCLWAINPAKVAKMDEEVQKWS

RKDPLAIKKAMIYPDHLELLERGEMKYAGNGDVSEETESSGDEAVEENTTYEESIHGHIAANSVTDSYDE

SSQDCDVDIQEHLYDEIDIEDNKDALHMHLNISKQEPFEYELSSSTKRQKTLTGAIQGNYVYQPVTTSRR

KTPFFLRAGTGSSSFLKID

>XP_012174951.1[Bombus terrestris]

MNYDLDKSRMVSHVIPVAGELQQGNGNLGVAGLGGMQESSHSLKIKQEPFQVSPPSPLPPLHQVSSFVSE

MQNGCIGVSKELDSTVSLARGLTSHHASLSHHHSHHSLHSPNSAVSMHSTGSTHHHLDEKGSSPSGALHS

TTNSNPSTPSTPSTPTTAADGSSTATGTGNTSGSNSDSASKKPPYSYVALIAMAIQHSAQKRATLSEIYA

YITARFPYFQKNKKGWQNSIRHNLSLNECFVKVPRDGGGERKGNFWTLDPQYADMFENGNYRRRRRMKRP

YRNAPYHKPLFGDPFSPTHVHLGPRNLFGHSPPSYAPSTYPRYDTSAWSLQQSQLSYSHCQSLQPQLQPM

QSMQIPTMNGYSQLGTSLTFQGNYLDVPGGTTSSPGSMGSGSFGSSFARRHDAAMTQETVPARCYWPEMV

NVKEEPGTVAVSSTSVGGVGVPSGMMGSTTPTGVSTSGFAPMEFQSRSKCFM

>XP_012171467.1[Bombus terrestris]

MLGIKVEYTEETNMMDSVPVSASGSKTGLQPQQGVPYDLESGFEPQTRARSHTWPLPRPEEYIEGNVVPS

VAASKEGVEGVESGTPPQHGSLGQGTGLLPVKKNSSRRNAWGNHSYADLITQAITSAPDERLTLSQIYEW

MMQNIPYFREKGESNSSAGWKNSIRHNLSLHSRFMRVQNEGTGKSSWWMINRDAKPGKSRRRAITMETSK

FEKRRGRVRKKIEALRNGGLQTDATPSPSNSVNEGLDLFPDSPLQPGSGFQLSPDFRPRASSNASSCGRL

SPIPAIPGKPEWTPTYTPSYSPEQLAGSLAETMKLESYQMYQTSPPSHQQQTGPPPSYYETQYQRSNSLS

TGSSSFALQPTPQSGNQQRCPLHGLQPCACQMNLSPVAGMSPSYQQSEPSPTALGSNQQQTLQYMMQTQQ

CQQQQQQQQQQQQQQSQTGATGSSPPQTPTPCGSTPSTMMGQLMGALNNSTLLDDLNINIESLHGGFDCN

VEEVIKHELSMDGTLDFNFQQGVMGTAAIQVSDSNVGQNGTVSQNNVVGTTAGNAPAGVYVSTNAATPAA

SPSWVH

>XP_003400940.1[Bombus terrestris]

MSTTYSRTQESDAWALLALKSAPASPTKMQWNPEAKGAPIARLEGREFEYMVRQHSITIGRNSSKGEVDV

NMGHSSFISRRHLEIYYDHPYFFMVCNGKNGVFVDGIFQRKSAAEFRLPKTCTLRFPSTNIRLVFQSLVD

EQEESNVCVRSPPKHRAPLPPLRINIPDAGYSSPFPSPTGTISAANSCPASPRAGQGRRNISADLQMVAV

YAAAVANDSQNSNMERHEGGQSSNRQISPGTTANCSPPKDDSKPPYSYAQLIVQAIASAADKQLTLSGIY

SYITKNYPYYRTADKGWQNSIRHNLSLNRYFIKVPRSQEEPGKGSFWRIDPQSEAKLIEQAFRQRRQRGV

PCFRAPFGLSSRSAPASPSHVGISGLMTPECLSREVSPGPESYPDSTVSSPAGQLTSQSAPGSPGHPYAS

SSQSSHKGRLMQQITVVTNGVSGDTTREDKYVVSGNTTEEHSLSPAGQYSPAPVIVQTTYNYSGSFIGPD

AGVGVAKRSHEESDSSPGSPAPLAIVESPEPPEHQPPSTKRQRVHDMDDH

>XP_003398898.1[Bombus terrestris]

MCSNESPPPAKEPLAVGLGNPMTGFLPGLEHYRLQLYHYAMAERLRLAQQLHPQHPGVGHPPSSAPTHLG

MGPGFPAPLPLYPAAAAAAGYPSRLALSMALLHPHHQRIPEEPKPQHSYIGLIAMAILSSPEKKLVLSDI

YQHILEHYPYFRTRGPGWRNSIRHNLSLNDCFVKSGRSANGKGHYWAIHPANLEDFRRGDFRRRKAQRKV

RRHMGLAVDEEPDSPSPPPLPATPPPPATLGPPVASQPMPGIWTPHHHHPHHQQQQQQQQTFQSQTLRQS

STFQPSRKRQFDVASLLAPDDQHQIESDLRPQKARRFSCSEDELHDLQEPDQDEEDVDVDVVAETNALPS

PNTPSASPEAKVISTAGHWTNQGIQSGQISGLHIQSHLQARNYYVPATSSNGSNV

>XP_012169351.1[Bombus terrestris]

MTSKYGVSISWVLCRVCLRCPSLENQRRLPSVYSDTDEKSTXPNMPPDRPDASGVETGIGLSSTKVFNFG

ETVYTMKEKENASAFVETNSTDELSMSDKSIEKNSQSDDDLTSLSWLHQQNLLKGLDISNPTKDMKNENI

LNNNVCDDMADFSENTNSVSSLDEGYCPADNNSKINNTTSHGNSQNYQHSNKNSQKSMQIFQESVKNHYS

TSQNNQTKISLNNNNLPVSNRNKHPTHIPYDPHLHRNSKPPYSFSCLIFMAIEDSPVKALPVKEVYAWIL

DHFPYFRNAPTGWKNSVRHNLSLNKCFRKVEKAPNLGKGSLWMVDAQYRPNLIQALSRAPFPPPTAQTLS

SPEKPQKKNTSTRLPDPILFPYLSKRLASSNITDNTDTEVDSDVDAAAAAMLSFKHGPIILNHNKDRKRK

VPESEVLVPVITRSSSEDHTYSCITSVKQESKYPRKETNPDFDEQRKIAEGADALLNLAGVTTPLNHNRT

HHVQGINSTPKSDGTSKLKKRSASNDYSNTPEKRRKHWPKWSEGKRYLKQII

>XP_012169563.1[Bombus terrestris]

MVKMKGESIDTKKPTNRGVSIMKENMHNDDYGVEAELTSLSWLQSLDITSASSLPTPPCSPSPPPLTRQP

RKKLSPLIKAELDLAANAEKYRTDPDAKPPFSYATIICLAMRANNNKVSLSNIYAWIRENFLFYKYADPA

WQNSIRHNLSLNKCFVKLPRSKDEPGKGGFWKLDLERMEEGRKSKRRATMSQRIRGSKKLTAAKTMVTNN

IQSNGAPSINCPSTLYETPPSSVSPPIPDCLSEIPDSTQVSLSEDDLTGLLIATAGWDENQLDLLDSLLD

TL

>XP_012169562.1[Bombus terrestris]

MVKMKGESIDTKKPTNRGVSIMKENMHNDDYGVEAELTSLSWLQSLDITSASSLPTPPCSPSPPPLTRQP

RKKLSPLIKAELDLAANAEKYRTDPDAKPPFSYATIICLAMRANNNKVSLSNIYAWIRENFLFYKYADPA

WQNSIRHNLSLNKCFVKLPRSKDEPGKGGFWKLDLERMEEGRKSKRRATMSQRIRGSKKLTAAKTMVTNN

IQSNGAPSINCPSTLYETPPSSVSPPIPDCLSEIPDSTQVSLSEDDLTGLLIATAGWDENQLDLLDSLLD

TL

>XP_003704862.2[Megachile rotundata]

RDDRFDLEEKIKHVAGGNRSKKSYSMMSSSGQQKSSTGQPSSYNNDKMSSSLIKPPYSYIALITMAILQS

PQKKLTLSGICEFIMSRFPYYHDKFPAWQNSIRHNLSLNDCFIKIPREPGNPGKGNYWTLDPLAEDMFDN

GSFLRRRKRYKRPPPHYVLRDRAIMATFAICGDRGPCPGSGGGHPGALAYPSAAYLSPPPGLPLLDFSPS

TLEALKLGGFLEPPPPLYKPVPITAPPMRQLDPTPTRTTTIPSSHPPVEKKRNFSIDALIGKQTASDQNC

GGLLDLSPSEHREIRSQASAFSPLV

>XP_012144318.1[Megachile rotundata]

MSGRPCAHEAITMEPRGMMMPNLSCDGCVDSDRDSDSSSMIVDPVSLDKNELSPSQSSSSPIISSSKKSY

SMMSSSGQQKSSTGQPSSYNNDKMSSSLIKPPYSYIALITMAILQSPQKKLTLSGICEFIMSRFPYYHDK

FPAWQNSIRHNLSLNDCFIKIPREPGNPGKGNYWTLDPLAEDMFDNGSFLRRRKRYKRPPPHYVLRDRAI

MATFAICGDRGPCPGSGGGHPGALAYPSAAYLSPPPGLPLLDFSPSTLEALKLGGFLEPPPPLYKPVPIT

APPMRQLDPTPTRTTTIPSSHPPVEKKRNFSIDALIGKQTASDQNCGGLLDLSPSEHREIRSQASAFSPL

V

>XP_012151060.1[Megachile rotundata]

MTMLQSQKLYSDAGSLGGAMTSATMSTMGSMAPTYSSINTMGCVSMGMSMGVGVGPSCSPQSAGGFNMST

MSSAVGMASMGGGGMGTYGSASMGGGSACMSAVGYGPLTTVGSAGVTRDPLSLAEPESPNSALQRARTDK

PYRRSYTHAKPPYSYISLITMAIQNAPTKMLTLSEIYQFIMDLFPFYRQNQQRWQNSIRHSLSFNDCFVK

VPRTPDKPGKGSFWTLHPESGNMFENGCYLRRQKRFKDEKKELTRQNNKHQQHQQHHGGPAAAGAAAAIA

AGHNSPSSHDLTHAGKKTPSSLHHGPQQQDDKDLHSLVSSHHHHHVSSLHQHHAALKSDGTDIGGLLGPD

LGAAHDELTAMVSRSLHPHLISDPAALHHGMTGSLKQEPPYTAASHPFSITRLLPGATAGTSPGAQDTKP

PEMKMYEQLHQSYANFGSPHHPHAHSAPPSHHHHNGMHTGSNAGGPMHNMTNHHHQEYYQSPLYHHATSV

ASSSAPPPPSVATAAPGL

>XP_012142878.1[Megachile rotundata]

MLEPRWRPVQGHIGENPFDNGSWGKEQFQPSTVPWQLNPRGHARPSDDGIMDHDTDGDGAINLSTSQRPS

AATTPNGDTPSYGQDQQDNDQATSLFAALKQHQQQPRDSVSSSRERENRERDRLSVRNRENTRSNELSGG

VTEQQQQQQQQQQQQQQQQQQQQDLTIEYQSNGKLSPAGLAVTTAPMTQQKQPIITQQSQQPSSGAPGPQ

PSPHQSPQAPQRASPPNPSQGPPPGGPPGAPPSQNPSQMMLNPASGIHQMQQLLQQHILSPTQLQSFMQQ

HTLYLQQQQQQHHQDTTSEHASNQERFGYFSSLKDHQHQFAELGRKKLEQAIQQLQEQLQLNVIQQSHLL

QTADKKKASAPLQQLALQQQRLIQQLQITQSQYLLQQGLGLQGHNPSSGLQPGEGLPMWKSETLDGPESH

QNSNVPKSGAGLNGLLNSTVSSRRSEMNGTTPLDEKPLDVSSNDKAHPLYGHGVCKWPGCEVICEDYQAF

LKHLNTEHTLDDRSTAQARVQMQVVSQLEIQLQKERDRLTAMMHHLHMAKQMASPEPPKSSESSTGSSIP

KLNLSTALMSQPPPNFGVSQVSPVSMSALVSAVRSPAGSQQQPSGGAPMPPIPNMSNMSGLPPFSNMPGS

MQSMAGPVRRRISDKLSLAGGLYDEGTVRRRVAVDRSGIDINEEIQRNREFYKNADIRPPFTYASLIRQS

IIESPEKQLTLNEIYNWFQNTFCYFRRNAATWKNAVRHNLSLHKCFMRVENVKGAVWTVDEVEFYKRRPQ

RACSTTGGVPSKSPTLTHSPTMYGDALNANIQYFQATLGDSNMGFLSNSMCTSMTTSPDKEHVLAHNDLM

SPLDEPAVHIKQEGQSPEGGKLTRLIKRELVDAPAEQEGEEDQVDEREYPESHGHDSGQDEDVAEDLSMA

PDIMTPEDQIEA

>XP_003702099.1[Megachile rotundata]

MDQTVIVPSTPRGTVDSTRIMKNESDASLHLPDQTSVAHANSASVVPRTGLSMSSNSNQDQSLDPLMCNP

SSTELPRKPGARRQEKPPYSYIALIVMAIQSSPGKRLTLSEIYSFLQQHFPFFRGAYQGWKNSVRHNLSL

NECFIKLPKGLGRPGKGHYWTIDPSTEYMFEEGSFRRRPRGFRRKCQALKPQYPQYFSGSGPVGVQAPGY

ENLTPGSMEYPNGYQNQYQNYQEYAMYAPGAAVSADWAYPEATYKTPPIAEVTYKTTEVTYKTGEPSVYR

NGEIVAFKSEPGYASRSQDQLAYRATEGFSVKDHQQHHPETVYKENDTMMSYKCPSNPAPTTQAPGQDYY

VGYGLAGVNNAGSNVNVPMQGIPEQTGNTSPGGNVSSPHSGCQTPVTDNGIKMQCSNSNSNSSGGGLIDR

KPSYFGHPAGSVTLSSLSSLSSLNLSNIGGLSISNIPGTVSSNIHHTTTTPSTTMYYDQIKYSM

>XP_012144745.1[Megachile rotundata]

MCTHSNNKYKLKLNIDSMIGPSSGTGGVPEHKVLDNQSRSGKKRIFEDLDIENFCEEVQNSRKRYQHQEI

DAPMEMVASNQGIGDTAALFSPMSSQETVDECPVARLEVLLVDGTNYTWTTVSELESHQNVHEIATSPSA

SSASSEHRQEQVIQEVQIEEMSYPVSSDYWEPVITVPSLNAQQNKTGGFYHSQQQQQQQQFDDQETGNLS

WLLDFKLDPFIEAADDKSTATPTRDNYAGVKVYSYSTPIKKKTVERKSLPLKCCHISQRLFPSFCSGNKV

KINGRPYGSVCSNDVKRSYTENGSLHQDSYQSYSNFDNRNFASSRCNGPKKPPFTYTELIEHALRERGEL

TVSAIYQWISEHFPYYKSNDDRWKNSVRHNLSINPHFRKGSKAPHGAGHLWAIANRSGDSRPKQTINAIN

SSIKQAVKHSIEAENNRKNISQTNPIDEVEAATASIIQQPSEEETGNIVNSVTLEHCAEEILSGIKKEVE

VQYLVPMMVSNNQHNSTQSDQQTQELHYPIKESDFLNPVSKEVVAEECGLISEGYLVTDLNPTTLGLNMV

DPEIITPENLFGEELSFQFYELSSPSQIQSA

>XP_003705315.1[Megachile rotundata]

MPRPSRDTYGDQKPPYSYISLTAMAIWSSRDKMLPLAEIYKFIADRFPYYRKDTRRWQNSLRHNLSFNDC

FIKVPRGPHRPGKGAYWALHPAALSMFENGSLLRRRKRFKLHKPDKELLKSELQALASAMPPPHSESSPV

ISINPAAQTSSLTVANLHRLRDDLLRWELHERRLTMTSGDSSPGFSAPEAGSSYYLLSPEVRQRLAGTDE

ILRGYESNLLQTGSWNFPGFQVTPYVSQLSYFQTDIPDSRQICPGTAETIPDKTESRPFLDTARSSLEPA

SGKRSTLEPGISSQTTIQPEQKKKKKPFTIENIIAPDDEQISGNVEDEKRGHLLVPRPLYAGFPFGLSTT

KVTYETAT

>XP_012138143.1[Megachile rotundata]

MHTLFGEQNAYYRHATAVPVGMGTPSYPGVGAAPGYYEQYRYGGYATAAGYPVSGITQQHIHHPGKDMVK

PPYSYIALIAMAIQHAPDKKITLNGIYQFIMERFPYYRENKQGWQNSIRHNLSLNECFVKVPRDDKKPGK

GSYWSLDPDSYNMFDNGSYLRRRRRFKKKDALKEKEEALKRQCLAPEKRSQDEAKPSNIVIPPPSDASSK

KLNSLDTALCKPKREPVNDAGSHCMAVQAKYGLHSPIQDTKTTATTTAAVAVAGQSVIQTALGHQVHPTH

QVHQDAIQDVSMGLDPTSFSVDALMTTRENTATLMTRDNQHHTHHPHGLQHPHPHSHSIMTSRESMGTGV

VSRDHLSSVCTTTTTTTAAVAAMMATSGYGHASTVSRSNGSPPGTMYAPYCAPTAGYIMDHAEYNSRNHN

NTAGHSQWYQEAASPDTAAIYQDTQSPSCQLYRSSPPTPLSTSAAPSPPLYHRYYQDCNTVNTSGNLPIT

LHKY

>XP_003702171.2[Megachile rotundata]

MESNVQGHNCNVSKMYGDLWRNYELWNTEEVFPTMCPLPSYYNLVPSTLNSRHVPLSWGSPLIRYKPQPR

YEKPPYSYIALIAMAINSSPKQRLTLSGIYRFIMDRFPYYRENRQGWQNSIRHNLSLNDCFVKIPRDKVI

GNDNEQDQAGKGSYWTLDPSASGMFEHGNYRRRRMRRQRGPGNDKQDQDQAIVSVSSKLLTNLKHQERND

GPEIIEVKDSKENARKMKTKYSESQGQRMKSMMFTIENILRKSTNESPI

>XP_003701245.1[Megachile rotundata]

MLDTESRHGTGLVQGPTRDPWLTMDYYLGTDSLSLQEMLDVDIKCEIEGVIGGHTELGFNFTDMSTLEMD

DDPIGCQNDISGWFGSTSFINGNSNSSNSNFNLDLSGSDAASIMVNPNSVMPHIAIRSPSPNNGRRHFSF

SMKKEIKEEKIDIEDEPENDTNSYIENENENENENDNENEIENENENENDNDNENENENENENGEENEIG

NDTETEQYENDITETEEDSDDEQELSKSVTPSKLKTVSISTIKTSSPQFIKTQSTPKINGMSGIRVQNFK

LLQSPQTPQHVRKQIYNNNVHVSPGATTVASMKREKEFDLSDYDDKPYPKPAYSYSCLIAMALKNSQTGS

LPVSEIYNFMCEHFPYFKTAPNGWKNSVRHNLSLNKCFEKIEKPAGNGNQRKGCLWAINPAKVAKMDEEV

QKWSRKDPLAIKKAMIYPDHLELLERGEMKYAGSGDVSEETESSGDEAAEESTTYDESIHSHIAANSVTD

SYDESSQDCDIDITEHLYDEIDIEEHKDALHMQLNISKQESFEYELSPSTKRQKTLTGAIQGNYLYQPVT

TSRRKTPLFLRAGMGNGSFLKID

>XP_012154430.1[Megachile rotundata]

MPPDRPDATGTETEIELSGTQVFNFEETSITMKEEENSNGLVETNSADELSMSEKSIEKNSQSDDDLTSL

SWLHQQNLLKGLDISNPSKDIKTKNVLNNNVCDDMADFSENTNSVSSLDDSYCPADNNGRMNNTTSHGNS

QSYQHSNKNGQKSIFQESVKTHYNTSQNNQTKISLNNNNLPVSNRNKHPTHIPYDPHLHRNSKPPYSFSC

LIFMAIEDSPVKALPVKEVYAWILEHFPYFRNAPTGWKNSVRHNLSLNKCFRKVEKAPNLGKGSLWMVDA

QYRPNLIQALSRAPFPPPTAQTLSSSEKPQKKNTSTRLPDPILFPYLSKRLASSNITDNTDTEVDSDVDA

AAAAMLSFKHGPIILNHNKDRKRKVPESEVLVPVITRSSSEDHTYSCITSVRQESKYTRKETNPDVDEQR

KLVEGVDALLNLAGVTTPSNPSRTHPGQGINLTSKSESTSKLKKRSAPNDYPNLPEKRRKHWPKWSDGKR

YLKQTI

>XP_003702563.1[Megachile rotundata]

MCSNESPPPAKEPLAVGLGNPMAGFLPGLEHYRLQLYHYAMAERLRLAQQLHPQHPGVGHPPSSAPAHLG

MGPGFPAPLPLYPAAGYPSRLALSMALLHPHHQRIPEEPKPQHSYIGLIAMAILSSPEKKLVLSDIYQHI

LEHYPYFRTRGPGWRNSIRHNLSLNDCFVKSGRSANGKGHYWAIHPANLEDFRRGDFRRRKAQRKVRRHM

GLAVDEEPDSPSPPPLPATPPPPATLGSPVASQPIPGIWTPHHPHHQQQQQQFQGQTLRQSSFQPSRKRQ

FDVASLLAPDDQHQIESDLRSAKSRRFSCSEDELHDLQEVEPDEEDVDVDVVAETNALPSPNTPSASPES

KVVSPAGHWTNQSLQNANGQQIPGLHLQNHLQVRSYYVPASSTGPSV

>XP_012138798.1[Megachile rotundata]

MNYADLERTRMVSHVIPVAGELQQGNGSLGVTGLGMQESSHSLKIKQEPFQVSPPSPLPSLHQVSSFVSE

MQNGCLGATSKELDSSIGRTLASHHSLSHHHSHHSLHSPNSVVSMHSTGSTHHHLDEKGSSPSGVLHSTT

NSNPSTPSAPSTPTTAADGSGSASGTGNGSASNNDSASKKPPFSYVALIAMAIQHSAQKRATLSEIYAFI

TARFPYFQKNKKGWQNSIRHNLSLNECFVKVPRDGGGERKGNFWTLDPQYADMFENGNYRRRRRMKRPYR

NAPYHKTLFGDPFSPTHVHLGPRNLFGHSPPSYAPSTYTRYDTSAWSLQQSQLSYSHCQSLQPQLQPMQS

MQIPTMNGYSQLGTSLSKAFQGNYLDVPGGTASSPGSMGSSSFGSSFARRHDAAMTPETVPTRCYWPEMV

NVKEEPGSVTVSSTSVGGVVPSGMMGSTTPTGVSTTGFSPMEFQSRSKCFM

>XP_003706042.1[Megachile rotundata]

MVNMEGSGEQHTVTASPPPRAALKSNFSIRSILPEACAGTPAPSVSRSTSPEISHVEDSEDSSDLDVTGD

GGNETPPLDCSRNATNSVSSSEPKDTKDRQSDEKKKCEKPPYSYNALIMMAIRQSPEKRLTLNGIYEYIM

RHFPYYENNKQGWQNSIRHNLSLNKCFVKVPRHYDDPGKGNYWMLDPSSEDVFIGGTTGKLRRRTTAASR

SRLAAFKRSVVLGGLYPSAYGPPGWPASLYTLPYLHRAAGYPPASSAYSTPAGYPASLLPGAATSSTSNL

PCKPQPLPATAAPPAHGPFSMERLLQPPTATGYPNGIATSGIPISGNPYDFYSTLRSLAAHQQHQTTAAM

FGHNQQPARYHVNQPPVLGQPTSATASPGSSPEPMSPHSPPVTICNSSSVQQPRSLLHSPPQLLLKPITV

LTGRQS

>XP_012147019.1[Megachile rotundata]

MVNMEGSGEQHTVTASPPPRAALKSNFSIRSILPEACAGTPAPSVSRSTSPEISHVEDSEDSSDLDVTGD

GGNETPPLDCSRNATNSVSSSEPKDTKDRQSDEKKKCEKPPYSYNALIMMAIRQSPEKRLTLNGIYEYIM

RHFPYYENNKQGWQNSIRHNLSLNKCFVKVPRHYDDPGKGNYWMLDPSSEDVFIGGTTGKLRRRTTAASR

SRLAAFKRSVVLGGLYPSAYGPPGWPASLYTLPYLHRAAGYPPASSAYSTPAGYPASLLPGAATSSTSNL

PCKPQPLPATAAPPAHGPFSMERLLQPPTATGYPNGIATSGIPISGNPYDFYSTLRSLAAHQQHQTTAAM

FGHNQQPARYHVNQPPVLGQPTSATASPGSSPEPMSPHSPPVTICNSSSVQQPRSLLHSPPQLLLKPITV

LTGRQS

>XP_012144591.1[Megachile rotundata]

MSFSLMVLSMILTDINPWPGSDTRILTRHSYSCPIRTNSVSFSGGNSLVLYPPSASMYLVICSVICLSIS

ICLLVLFLSLILSRRRVCSLGAMSTYSRTQESDAWALLALKSAPASPTKMQWNPEAKGAPIARLEGREFE

YMVRQRRITIGRNSSRGEVDVNMGHSSFISRRHLEIFYDHPFFFMICNGKNGVFVDGVFQRKGAPAFQLP

KTCTFRFPSTNIRLVFQSLVDEQEQNNVRLPSPPKHRAPLPPLRINIPDTGYSSPFPSPTGTISAANSCP

ASPRAGQGRRNISADLQMVAVYAAAVANDPQNSNMERHDGGQSSSRQISPELGVESRYRGGSSSGPNGTT

ANCSPPKDDSKPPYSYAQLIVQAIASATDKQLTLSGIYSYITKNYPYYRTADKGWQNSIRHNLSLNRYFI

KVPRSQEEPGKGSFWRIDPQSEAKLIEQAFRRRRQRGVPCFRAPFGLSSRSAPASPSHVGISGLMTPECL

SRETSPGPESYPDSSVPSPAGQLTSQSAPGSPGHPYAPSNQSSHKGRLMQQITVVTNGVTSDTAREDKYV

VSGNVTEEHSLSPAGQYSPAPVIVQTTYNYSGSFIGPDAGVGVAKRSHEESDSSPGSPAPLAIVESPEPS

EHQQPQTKRQRVHDMDDH

>XP_012145278.1[Megachile rotundata]

MDPVSVSASGSKTGLQPQQGVPYDLETGFEPQSRARSHTWPLPRPEEYIEGNVVPSVQAKEGVEGVESGT

PSQHRSLGQGTGLLPVKKNSSRRNAWGNHSYADLITQAITSAPEERLTLSQIYEWMMQNIPYFREKGESN

SSAGWKNSIRHNLSLHSRFMRVQNEGTGKSSWWMINRDAKPGKSSRRRAITMETSKFDKRRRQVRKKIEA

LKNGGLQPDATPSPSNSVNEGLDLFPDSPLQPGSGFQLSPDFRPRASSNASSGGRLSPIPSIPGKPEWTP

TYTPSYNLEQLAGSLAEAMKLESYQMYQTSPPNHQQQTGPPPSYYETQYQRSNSLTTGSSSFVMQPTTQS

ANQQRCSIHGLQPCACQMNLSPVAGMSPSYQQSEPSPTALGNSQQQTLQYIMQTQQQSQQQQQQQQQQQQ

QQQPQTGATGSSPPHTPTPCEPTPSTMMGQLMGALNNSTLLDDLNINIESLHGGFDCNVEEVIKHELSMD

GTLDFNFQQGVMSTAAIQVSDSNVGQNGGVSQNNVVVGTTAGNAPAGVYVSSNAATPAAPPSWVH

>XP_003706526.1[Megachile rotundata]

MVKMKAETIDAETPIDCDSSDSKENMHSEDYGVEAELTSLTWLQSLDITSASSLPTPPCSPSPPPVTRQP

KKKLSPLIKAELDLAANAEKYRTDPDAKPPFSYATIICLAMRANNNKVSLSNIYAWIRENFLFYKYADPA

WQNSIRHNLSLNKCFVKLPRSKDEPGKGGFWKLDLERMEEGRKSKRRATMSQRVRGTKNLTLAKTTVTNS

AQNNGASSINSLSTLYETPPSSVSPPIPDCLSEIPESTQVSLSEDDLTGLLIATAGWDENQLDLLDSLLD

TL

>BGIBMGA005101_BmFoxA

MISQKLSYGD VPTSASLSSL SPGLAPPYVN GMGCMPAQPY PNLYSNNMVA GGSCMGSPSV

GYSPPSTMAS CMGGAGAVPY GSLPREQEAD SPTSALQRAR NDKTYRRSYT HAKPPYSYIS

LITMAIQNNP SRMLTLSEIY QFIMDLFPFY RQNQQRWQNS IRHSLSFNDC FVKVPRTPDK

PGKGSFWTLH PDSGNMFENG CFLRRQKRFK DEKKETLRQA QKAQQTHGHH GGSHDKRGEH

GHDKSAPPGP GEDKEMRDEL LAQLHAAPEL CLPEHTPLAL EHYAQLKQEP SGYAPAQHPF

SITRLLPGAD TKADLKMYDV NYGYGHSPAD NYYQSPLYHH HHAHAQPPL

>BGIBMGA003547_BmFoxB1_BmFoxB

MPRPSRDSYG DQKPPYSYIS LTAMAIWSSP ERMLPLSEIY RFITDRFPYY RRNTQRWQNS

LRHNLSFNDC FVKVPRRPDR PGKGAYWTLH PQAFDMFENG SLLRRRKRFK LQKGEKDNLN

AELAALANFN RAFLARQAAG PPPPASIPTA TLYTSTLCSQ LSPEPAELPD VTPITMLPRE

RPRRAFTIDA LLEPDSPRLS PSPPQPMISL EARMAAPYVL AAQRYHAELL QAAAHALRPC

YLPPPPLPVA

>BGIBMGA003641_BmFoxB2_BmFoxB

MAIWSSPERM LPLSEIYRFI TDRFPYYRRN TQRWQNSLRH NLSFNDCFVK VPRRPDRPGK

GAYWTLHPQA FDMFENGSLL RRRKRFKLHK GEKDSLNAEL AALASFNRAF LARQASAPPT

PTNASSGGLY PSASTYMSRL SPESTEAPDT AALLPVAPRP RRAFTIDALL EPDPRRSSPS

PPLQLPPLQP HCPLPLPAPY LLAAQRYHAE LLAGLQQSCL PPLWTWRESN QFSHYTLNS

>BGIBMGA013330_BmFoxC2_BmFoxC

MHALFGEQSP YAYRGGAAGG YSAGVAPYAY EQYRYGYGAP YLHPHQQHVG APKDMVKPPY

SYIALIAMAI QNAPERRITL NGIYQFIMER FPYYRENKQG WQNSIRHNLS LNECFVKVAR

DDKKPGKGSY WTLDPDSYNM FDNGSYLRRR RRFKKKDALK EKEEALKRQQ QLQQAQEALA

AQEALSAADA LGQQRDVKPD VKPRIFECRP KREPGAECTR YDKPVDSIDE FNEPRLPPSA

VYCSPQPYSL AAEDFRAATS GWYTAPEPPA EQLPPAFRDL FEPPSCQLAG FRGGSPPPEA

YRASPPHHHY RTPAPSYYHH QACVAAAPAP AHKSY

>BGIBMGA001488_BmFoxC1_BmFoxC

MCSGAEGGSW PLGKDAPTVT AAAIDHYRLQ LYNYAVAERL RLYPPTVAPC YGPYGPRLAL

SMSLLQQRAL QPEEPKPQHS YIGLIAMAIL SSPERKLVLS DIYQHILDNY PYFRTRGPGW

RNSIRHNLSL NDCFVKAGRS ANGKGHYWAI HPANVEDFRK GDFRRRKAQR KVRKHMGLAV

DDDGEDSPSP PPQSPPPTAL PLPFWNAARL AGGGQPRKRQ FDVASLLAPD ETPEKRQRRD

SSCDEEPEED IDVVASDQEE RGVDEETRGP LTTATQYPLL GGWWPALEPA LLQQLRRHAT

PAPPSPPDQH RPPDI

>BGIBMGA004141_BmFoxD

MEDCDLAQYR DMIIEGELPL DLRPPPRLYL PHYLRDDGDS EYPQDDTYVS VDEDTPDLPP

PSSDSERYDA PSPTDKKPAS PKKPKQKSSS QLIKPPYSYI ALITMAILQS PHKKLTLSGI

CEFIMTRFPY YREKFPAWQN SIRHNLSLND CFIKIPREPG NPGKGNYWTL DPLAEDMFDN

GSFLRRRKRY KRPAPSLQHA HAVVAMLARE AYAPLLPLPC YLPPTPLLAL PRPPPATLRP

VPLPPRVSET TEPRSKRSRN GFSIESIIGS QESMSEPRRS AFLPLHPGNS PPDDWAR

>BGIBMGA012276_BmFoxF2_FoxF

MKTEDQTTEE AARRPPATRR QEKPPFSYIA LIVMAIRHSP NKRLTLSEIY AFLQQQFPFF

RSSYQGWKNS VRHNLSLNEC FVKLPKGLGR PGKGHYWTID PSSEFMFEEG SFRRRPRGFR

RKCQALKPQF GSGGYLCGGG MPTLPPSQPT GYELAGSSSS GASSGPAIDY GACAYSHAGS

TSQQLGYGSE YCTYGTMSER EWPLAYGAVE PGYRPPPTSP PPPHHDLPDI IPNYQYAVAN

EHGKFLSFTL LKHLCPILIN Q

>BGIBMGA010490_BmFoxG2_FoxG

MSPPHSFAIR DILPEALSRS PSPSDAELDV TGTETPPPCA STSNENKKNE KPAYSYNALI

MMAIRSSPEK RLTLNGIYEY IMKNFPYYKE NKQGWQNSIR HNLSLNKCFV KVPRHYDDPG

KGNYWMLDPS SDDVFIGGTT GKLRRRSTAA SRSRLAAFKR GALLHPMFGN PYASLVGLYP

PLLSVYGRYA LPSPLLRLPP PPPSPYHQLY RRLHGLAAPT SPPPEPDHLV KHS

>BGIBMGA010297_BmFoxG1_FoxG

MVKFEGDFSI NSILMNHAAS KAPLSPAPST EASPSETDLS DSELDVTGTE PVDCSKPKDD

DNGEKKHEKP AYSYNALIMM AIRNSPEKRL TLNGIYEYIM TNFPYYRENR QGWQNSIRHN

LSLNKCFVKV PRHYDDPGKG NYWMLDASAD DVFIGGTTGK LRRRSALTGR ARLACFKRPL

FPGAAPYPPA AYSQLVGLYS QLLYQRYAPM QMKTMPVAPS AVHPAFRDAV GYSALQYSPS

LYDRMPTAPF LGQASLLANT PLAQPQLLTP SPPSLHSPPT SGSSSPELPS PSHHPLSPHI

YKPVTVLTRQ

>BGIBMGA005669_BmFoxJ1_FoxJ1/4

MLDTATIHFY GDSSEQYGEA YFAVDHTVPE SSHTVEIEYV YEQPESYTIN RDDANKQTPT

NKPKHAQEGV PKKSLKKMKP EKVIDDEQDL TNLTWLQNIT NIMAVPQFPI SPMSPTPPLK

SQNTRLQKFN QTIAKCQNDF MENKEEYQRN SDKKPPYSYS TLICMAMRYN NDKMTLSAIY

SWIRENFKYY RNADPTWQNS IRHNLSLNKV FVKVARSKHE PGKGGFWKLD LAHLEGSKRI

SNRAHKKKTK SSPDTRADNT SEEGKAVERI LQDSITNEMY EPITLNLPDY NLQSIESLDN

IQLSNNIGAN VIVEPAIPPP LLPEDDLTNL LLNPTEWDDL QLDMLDNYLD FSFK

>BGIBMGA014268_BmFoxJ2_FoxJ2/3

MSESPGVEWL PAYSPGTPRH SPLGPIPRFL YEDVQPLALQ QENNNKDEQK ENTNKIKHAK

PAYSYASMIR LAISSSPNGK MTLNEIYTYI CNAFPYYKEA GKGWMNSIRH NLSLNKCFMK

VARSKDDPGK GSYWAMDTSY KMAEVTPRRR RSLRMAPYSP ECSSNSSGEA GPPAATPTPP

PTPTTPTTPT SPHVLPVKEE PVTNEQSDSK HTDDALSALM NEELMTDVDI TRERWLCEPG

RRHVCVVARH SSLTDDYGNF GHNPPRPDDC DCPSDPQYYE PDAL

>BGIBMGA000635_BmFoxL21_FoxL2

MTPSSPGNSE IKAQTTSSNS QALTKPPYSY VALITMAIQN SQTKRATLSE IYAYITKEFP

FFEKNKKGWQ NSIRHNLSLN ECFIKVPREG GGERKGNYWT LDPQCGEMFE NGNFRRRRRM

KRPFRANPYS KALFGDGYHV SHVAQHVPPH MQPLPLGPRN YFGSGSPYHP PSYPRYDA

>BGIBMGA000838_BmFoxL22_FoxL2

MATKTPESEK WRRRKLSKNI DNDNDCASLA WLLNFRLDEV VNVRVPDDEP VVKEAKVDVE

SPAVRKPPYT YPELIERALR ENGELTVSGI YQWISDRFPF YKANDERWKN SVRHNLSINP

HFRKGARAPQ GAGHLWSLAA NAIDLLPLRN TPMPEEKAAE PLHTAKIIGF PKVIVLDEAA

IAAASIIPDQ DLFSGSTMFL NPVSAEQVIR KCGLITVD

>BGIBMGA000445_BmFoxN4_FoxN1/4

MSLICQCTHK HISVPKEVIS WCSDMDLYIT DSLQDMLDID IKNEIATDLS SISDFQIGTK

TIISGNIHIL DAQSIGQSRT ILANGNKNQA TILIDNSSLS NGRQLMKTSI SNTYTVDNSQ

TKYLSAHGKT VGEFPKPAYS YSCLIAMALK NSRTGSLPVS EIYNFMCQHF PYFKTAPNGW

KNSVRHNLSL NKCFEKIEKP STNGSQRKGC LWAMNPSKVG KMDEEVQKWS RKDPQAIKKA

MVYPESLEAL ERGEMKYSAV GGDTDIEDDA DVDGDTEMDA DVEIDPEVKE EVEEEETVEQ

EVSDQELEVD EVVEGTGMVG GTYRLLATGP SSLTSYITDV ESGEEVSDVE VLDNSYEEVD

FGKPIKFDLM TENYTKFGKV RALAQSALLF

>BGIBMGA008311_BmFoxN3_FoxN2/3

MDLEETDAVR GQLNERLCTG ALSFSSRDSP RTFDSSYYNV YIRILGLPRG VSTSMYIQRD

VFRLTDYTTT ISRKLPGSRP CGIIARGIVA VIQCAPSLGR MLKRIVKFFT ILVESIAGNS

SWAEVACGGN AGNLPPADND DDLTSLSWLQ DKNLLRGINL TKSDIEDTKT MGSQIILKTE

SKTPPPVVRP PSPPRTPVKA PPSPPAVTHT KPPYSFSCLI FMAIEAAPAR ALPVKEIYAW

IIKHFPYFKH APQGWKNSVR HNLSLNKCFH KAFGRQPAPT PAGAAEPSGG KNSPDPTLFP

YLARRLAAEP GADEYLAAAT VLAMKYGPSV LEQLPPAASL VISRCTRDEH SYSGGGGEER

RTAEALLHLA GLPPSPALAP S

>BGIBMGA008092-BGIBMGA008094_BmFoxO_BmFoxO

MSIQGGGGYQ SPWSSQTGLS ELEGTMAELE PLGELAEVGF EPQTRARSNT WPLPRPDNYV

EQVDEAGSKK NSNQNLSGAP PIPAKKNSSR RNAWGNLSYA DLITQAITSA QDNRLTLSQI

YEWMVQNVPY FKDKGDNNSS AGWKLWLRTV LSRVIRDIG

>BGIBMGA004582_BmFoxP1_FoxP

MLSLGSGVGT EPKSRWGREA AAYQLIRAGP LSGPLSTAVT EWLGPLQPGA RVVSRGQRGR

HRWGSLSATT GRTGPARNAA DRPAHAAPGL NETKALPSAP DAKTGSLHQQ LGAQQQQLVQ

QLQAVQRQYL MHGPLSVPPN APPGLMLWGN EMEEHPLFGR GVCKWPGCDA LADDYQAFLK

HLEAAHTLDD RSAAQARVQM QVVAQLELQL RRERDRLAAM MRHLHAARDH HAHKHASGGP

SEGTSPGPVR RRVSDKSGVA IAGEIQRNRE FYKTADVRPP FTYASLIRQA IIESPDKQLT

LNEIYNWFQS TFCYFRRNAA TWKNAVRHNL SLHKCFMRVE NVKGAVWTVD EVEFYKRRPQ

RAAHAPPPMH HAGYARPTNA LYRLTHSHCL HHSSHHTRTV YITVPIILAL SM

>FBpp0303365_FoxA

MQKLYAEPPPSSAPVSMASSGGGGPPSGGGGGGGGGGGGGPPPPSNNNPN

PTSNGGSMSPLARSAYTMNSMGLPVGGMSSVSPQAAATFSSSVLDSAAAV

ASMSASMSASMSASMNASMNGSMGAAAMNSMGGNCMTPSSMSYASMGSPL

GNMGGCMAMSAASMSAAGLSGTYGAMPPGSREMETGSPNSLGRSRVDKPT

TYRRSYTHAKPPYSYISLITMAIQNNPTRMLTLSEIYQFIMDLFPFYRQN

QQRWQNSIRHSLSFNDCFVKIPRTPDKPGKGSFWTLHPDSGNMFENGCYL

RRQKRFKDEKKEAIRQLHKSPSHSSLEATSPGKKDHEDSHHMHHHHHSRL

DHHQHHKEAGGASIAGVNVLSAAHSKDAEALAMLHANAELCLSQQPQHVP

THHHHQHHQLQQEELSAMMANRCHPSLITDYHSSMHPLKQEPSGYTPSSH

PFSINRLLPTESKADIKMYDMSQYAGYNALSPLTNSHAALGQDSYYQSLG

YHAPAGTTSLXHHQYPLAXGRAEQMLRSQQQQHLQQQHQQHQQQQQQHQL

HQQQQQMQQSAQQLTSASNTPATSAKASGKAGSASASGSGSGSGSGSGSN

YSQKLQQQHQQQQQQQAAAQQQHHQQQQQLLQELQEDSSNITSDLSEEQL

QQHQAAQQQLYNNYQQYAAAAGYRNWNHGLAFNGAAALQNLL

>FBpp0084145_FoxB1

MPRPSRESYGEQKPPYSYISLTAMAIWSSPEKMLPLSDIYKFITDRFPYY

RKNTQRWQNSLRHNLSFNDCFIKVPRRPDRPGKGAYWALHPQAFDMFENG

SLLRRRKRFKLHKNDKDLLNEELTALANLNRFFFTTRNGGSAAHMSPLDM

NNAAAMRLDPLPRSTAHMPNSLGPGVPLPHVMPASMSGADHTNLADMGLT

NLPALTSSEIEGPLSLRPKRSFTIESLITPDKPEHPSEDEDDEDDRVDID

VVECSGISRYPTTPAASEEYMSASRSSRTEDPLPPMHTINAGAHVPFLHY

ATGANVAGLPASGIPNSPTTYELAISHPLFMMAAPIANMHNIYYNNVTLV

APAQQYRSPEVQNRIDNDMRTI

>FBpp0084146_FoxB1

MPRPLKMSYGDQKPPYSYISLTAMAIIHSPQRLLPLSEIYRFIMDQFPFY

RKNTQKWQNSLRHNLSFNDCFIKVPRNVTKAGKGSYWTLHPMAFDMFENG

SLLRRRKRFRVKQLEKDISNWKLAAAANTEMVTHYLDDQLTQMAFADPAR

HGHVLANASAAQMSPYKATPPILPTTVTQLPARPKRAFTIESLMAPDPAS

TPNEGLVPMEYGSPDAVALEKPPFNLPFNFNELAAQYQLYFPSFFYNGQY

GNIPCYQKTPPLFHNGPLPVF

>FBpp0078049_FoxC

MHTLFSDQNSFTRHYAQTAAGYGSASAVAAASSASAAAAAHYAYDQYSRY

PYSASAYGLGAPHQNKEIVKPPYSYIALIAMAIQNAADKKVTLNGIYQYI

MERFPYYRDNKQGWQNSIRHNLSLNECFVKVARDDKKPGKGSYWTLDPDS

YNMFDNGSFLRRRRRFKKKDVMREKEEAIKRQAMMNEKLAEMKPLKLMTN

GILEAKHMAAHAAHFKKEPLMDLGCLSGKEVSHAAMLNSCHDSLAQMNHL

AGGGVEHPGFTVDSLMNVYNPRIHHSAYPYHLNEDNLATVASSQMHHVHH

AAAAHHAQQLQRHVAHVAHPLTPGGQGAGGQSSGHSPTTISTPHGPAHGG

WYTPETPPSEPVPHNGQQGTPTHPGHNNNNSSSVLNHNGVGNGGGGGGGG

GGGSSSVLTSSPTSALGFRDMIFEQNQSCQLDTGSPTGSLQSASPPASAS

VAAASAAAAAAVISSHHHHHHHHAALSGNLGQLGQLSNLSHYRPHVGHYQ

EYGIKYGV

>FBpp0088311_FoxC1

MAHSDICALQTTTADQDQGTSSRDSNVPKPSLPNIYPLGTTRTSQAQSTT

MTLEQYRLQLYNYALNIERLRCPQYGGTGGSGASAPWLHLSPYGHHGSGS

LPSVNRMAALSTISLFPQTQRIFQPEEPKPQHSYIGLIAMAILSSTDMKL

VLSDIYQYILDNYPYFRSRGPGWRNSIRHNLSLNDCFIKSGRSANGKGHY

WAIHPANMEDFRKGDFRRRKAQRKVRKHMGLSVDDASTDSPSPPPLDLTT

PPPPSSQSALQLSALGYPYHQHYIGQFFNRSSAPGMTHYSPPDPALLMQR

QEANNLDQTIQPTQLQQPHSHHQHFAYINSTTTTTIANMFSQTRKRQFDV

ASLLAPDVQIVDIVSEDQESSVTPTTSARTTTQTHHTVITKQTIHREVVL

GLEKPVQDADIDADIDVEVNVDVVDDSIIPDTDSYTDGEDRKKTRGTLKQ

IFSIEDNNSYLIDGRLSSFDADPDHDPDHDPEDLEQHNFSIASSAARSLG

SSSSQHEESSSLEECPIQECIVAPQTALISSMATAKTSTLSVPISDAYLE

LNQVDQHMLSRYYGSYIAAAARRASIDASNTSRTSSITPPPKIEILSQK

>FBpp0071809_FoxD

MHTSTDPMHTLHDSVSLSPPLIKSSRGGSSIGNGIGCSASSRTAAADAMS

MGCDDSDIEPSSMGGSGAAGGNGDGSGSSGGPLVKPPYSYIALITMAILQ

SPHKKLTLSGICDFIMSRFPYYKDKFPAWQNSIRHNLSLNDCFIKVPREP

GNPGKGNFWTLDPLAEDMFDNGSFLRRRKRYKRAPTMQRFSFPAVFGTLS

PFWIRKPVPLVPVHFNVPNFNGSREFDVVHNPADVFDSALRADKKFNFFA

NAEASFYQGSQSGDKFDRLPFMNRGRGADVLDALPHSSGSGGGVGGGSES

SRGSKYKSPYAFDVATVASAAGIPGHRDYAERLSAGGGYMDLNVYNDDAD

TEADAEAEGDDDSCEDKIDVESGNEQEDSHISDSVDSACTNRLDAPPEAL

IFEASAEASDSSPRRRFDSEPLVLRTSKQSSAKDFRIETLIGHHLHRGGS

QEETSD

>FBpp0076609_FoxF

MIKSEEASDRSVMTMDQLGGYYHDPRAHPPFSHPHAHSHPHQHTHTGHPY

RAGNLLTGGNYQAMGGGESPTELIDEKPNIGYMELKHYMDATPTATPVSA

AQHYSLSALHSMGTPPASSSPIPPYGVLMTAHSAGSASPQSNSKTPTDLP

QDLQYASSSTSTAKVQPLQVQLQPLNHQYASTIKYCSNNTILSANDYQLL

TSQEQAGQQQPQQLPAQQLQHSPGGGYMSRISTSPSQVISNAHGMPVLNY

SSSSSSPAKSLNGSESSPPSQNHLENKVSGSAVVGTGGSSQQDAPSTPDT

TKKSGTRRPEKPALSYINMIGHAIKESPTGKLTLSEIYAYLQKSYEFFRG

PYVGWKNSVRHNLSLNECFKKLPKGMGVGKPGKGNYWTIDENSAHLFEDE

GSLRRRPRGYRSKIKVKPYAGHANGYYASGYGDAGMDNGNYYASPAFASY

DYSAAGATGVSPAGGQGFADPWNAHAAHSGSSSVGVGMGVGPLPQYTNIS

CLAAGGNVNGSATTPPLAHSALGMAPSASSSSSPLGAAATLQSDYAPTAS

LVAAGYSYATSAGSLDNGLRSISLQQLPGLSSIQHAQAQAQAQAHHHHHQ

HHASHPSHSHQGHGSMHQNHGTSSTTPPPSQSGGSHGIDHSPIDRKPAYL

PPISPPPMMVALNGGGGYYEGLKYAN

>FBpp0077188_FoxG

MVKSEMEFKSNFSIDAILAKKPINTATQPIKTEPVHHHHQYVHPYSNSDG

ELSASEDFDSPSRTSTPMSSAAESLSSQNNDKLDVEFDDELEDQLDEDQE

SEDGNPSKKQKMTAGSDTKKPPYSYNALIMMAIQDSPEQRLTLNGIYQYL

INRFPYFKANKRGWQNSIRHNLSLNKCFTKIPRSYDDPGKGNYWILDPSA

EEVFIGETTGKLRRKNPGASRTRLAAYRQAIFSPMMAASPYGAPASSYGY

PAVPFAAAAAAALYQRMNPAAYQAAYQQMQYQQAPQAHHHQAPHPAQMQG

YPQQLNAELFQRMQFFGKFPSS

>FBpp0077189_FoxG

MVKIEEGLPSSEISAHSLHFQHHHHPLPPTTHHSALQSPHPVGLNLTNLM

KMARTPHLKSSFSINSILPETVEHHDEDEEEDVEKKSPAKFPPNHNNNNL

NTTNWGSPEDHEAESDPESDLDVTSMSPAPVANPNESDPDEVDEEFVEED

IECDGETTDGDAENKSNDGKPVKDKKGNEKPPYSYNALIMMAIRQSSEKR

LTLNGIYEYIMTNHPYYRDNKQGWQNSIRHNLSLNKCFVKVPRHYDDPGK

GNYWMLDPSAEDVFIGGSTGKLRRRTTAASRSRLAAFKRSLIGPMFPGLA

AYPQFGQFLTYPPTAPSLLASMYQRYNPFAPKGGPGHPGLPPGLPGLPGP

PGPQGPPGPPPPPFVAPPTSSELYQRLQYQQLLHQHAAAAALAAHQRQLS

VAAASAASQPPPTHHHPHLAVGQAPLSPGGDSPGPSPQPLHKPVTVVSRN

S

>FBpp0088237_FoxJ1_FoxJ1/4

MSYHFTSSAKQNVTLQSCKEENLLSLEEEDSDEERELTNLNWLLRNQNLT

WPKTIDYNPTEILNSKRNTEPTHKSRVHDKQIKMFYSTRENTDKISHIIL

EANAQKSITKRPTASERFEIFVNKIKRDLTEYEKLASKYETDVTEKPPFN

YSHIIGMAMLQNGRITLQQLCSWIEAKFAFFRVRKKWNNSIRHNLSLHHC

FRNRKREERGKGGYWELGVDPKKCDRKRIRNRKIFHPTQNQTAKLQYEHL

TEIQQAKSARKNHSRHIKKSKTSSLPETSEANIFNVVPHKKHNIADENDG

QRQLQTINKDDILKKKSELDTIVSSTESFLTESQNLNCFNSHKTDTTNTP

ISAEKNFCTFNNFYSEMTPVLASNSNIVEDQNVSTDVSWPSHPCNITINY

DYTNFQPIVDSIAEQFQCLHDSAGYDRNDDILENLLDVCVTPY

>FBpp0304898_FoxK1_FoxK

MAATTTTTTTTLATTTGNSSSTNNSSSNNNNSIASSHNNNNNIVTVHTVL

NNPPTNTAGGGVADAATVAIAKDMLQLHHGAAAAATAALNNYNNSSNNNN

NSADQLTGNSNNSANHPPATSSTTNSSSSSTTSANTNNPSANAASTLTSS

SPPYGELRFNQEVLPITDAVTVIGRNSSTSLVHFNVAENNLVSRKHFQVL

YDVELRAFFVQCLSKNGIFVDDFLQRRNVDPLRLPQRCYFRFPSTEIRIE

FESYVPATSSDAIDGHSPSLVVGGGGGVAGGGAHVIITPLPRDELHQQQL

HHHQQQPHLQQQQHPHHPPAHHLPLQQQQQQQQPAHHPLPHTPHHPLHHT

ALHQQQQRSGSIVVAPPAGAAAHLIAGDGPGIYSPLKISIPKKEQKSPYL

SPTGTISAANSCPASPRQGFIQNQPNNYNNYGNNNTQDLFQTPSTASYNH

NEKPPYSYAQLIVQAISAAPDKQLTLSGIYSFIVKHYPYYRKETNKGWQN

SIRHNLSLNRYFIKVARSQDEPGKGSFWRIDPDSGAKLIDHSYKKRRQRS

SQGFRPPYGMPRSAPVSPSHMDNSRESSPLQDIVLQSAPGSPGMSLEQRA

ADPEIIYNSQNAHQQQQQQQQQQQQQTLSNNSNQYSSGSPYYVTNQSSGV

ATPQTHVEGSAASGGGGGGGVGALLALKRNHVMGGGASQHTLHQQQAVAQ

QQHSEIIYEELPTDYSGHIEASEEECVTTATDATVAKRPKYVSEVLXCVR

IQKHRQNKGK

>FBpp0073048_FoxL1

MLPSCYANGSMLPDNEELVNSMLANPDYLRTQVSPNPLAPSAVGGAGMEG

LMCGSFSPAFYYQGIDSFLALHNNIWGLPISFLHNSHRPEKPPFSYIALI

AMAISSAPNQRLTLSGIYKFIMDKFPYYRENKQGWQNSIRHNLSLNDCFV

KIPRDKNTIEDNDSAGKGSYWMLDSSASDMFEQGNYRRRRTRRQRHCGHP

NRYERESGKDSNDGNSSAAEIRSPSEPLSDFDIFCNERPNYSDRITDLHR

QYLSVSLGFNSLFNNEARGLRPLPEIRECPDDVDASSSSSKAMQSSMELH

EELHSPSAFTPPLNRRETSSSGAPVLAEAFNGIKDVVDAPGSSPVASSNR

SKTTLFTIDNIIGKP

>FBpp0070574_FoxL2

MWLKSKTNHRKTSRPKKPPFTYTELIEYALEDKGELTVSGIYQWISHLLP

PAPTIDQTTLTVLCRASKEQKRRLHGAPSRNTQLLEMSLHLNDLRHINVD

MYIKAAKKKHELPS

>FBpp0307179_FoxL2

MIVNMPVNGTGSASSPTSNPSSNNNNNSSTTNQNDTNTNLPGDYNFLKRK

FDDQEDLALGHDYEPIGANPWKKRHLDYDAGDLHFHLQLEQPPSNPPTNA

APAQSSTSFINDLFSYESSLLVPAVAYSHCPPSSAPLDDNGGWTGGELLE

LDHRYNSGLQAELGQLNATLPPPPPTSSSAQNASNHTQQSTQQNVSPGQN

PHQNQHFLVPQKQRIQSAGRRTSSGSVSGANEATEADFEDKNLSWLLNFK

FDEFPHLSPHNQIHGQAVNLPEGSAPPHPPSTAVPIATSPCSSSSPSSAS

AASASASAISTNQSLGSGLVDQRARSPKSCSSAAMPAALGGGGNGSAGAS

AAGGVAGVGSTKTGRKFEELVMEVTSELDGNDMIVAEHVVVEDTSSKAPK

KPPFTYTELIEYALEDKGELTVSGIYQWISDRFPYYKSNDDRWKNSVRHN

LSINPHFRKGVKAPQGAGHLWAISSGDSAENVLAWEHKKQRLDLFFKMES

INRERIQQHQQQQQLQQHQQQHQQHSPQQKQHSAAQQQHMPQASSCMYDE

AAVAVATLTQEMMQTNSGGGSTETSQQQQQQQQHHHHHSHQNHQNHNQHH

QRLLPFNDLLSDDELRKTAGQILNGIHREVEVQSVNSIISTYHDVLLDND

YLNPIHKDVVVTESVLRQQHGTNLGGGNNNQNNQMGGNSHMGGGNSQYYI

TEIDPMELGIQMSHQVEPSEEEVLFSDEFNLNYFGYNPGSDIVA

>FBpp0081715_FoxN4_FoxN1/4

MFELEDYSSGIHEGFFSKYADAAGPSLDFYVSDSMQEMLNVDIRAEIANV

VGSSSSDLTSSLDQTLEAISAINNNQSNGNSSQSASYNANANFLTSSGLH

ASPTAKWMGSSANFWSNSDYYADLGACVNPISVMPLINSTSAGMFSPKKN

KTASSTQGRSGAVPSSPSAERDQHKSHLTFSPAQMKVSAGSMRRDQVMAH

IPKQISVVTGTGTTAPATMATNSVLQRRNSSAVDAVRKDLVTELRKAQSS

PVPNSLEELGKGKGSTLLNASVGATNTIKLAPGIGGLTFANSAAYQKLKQ

TSLVKSPGGISPGAGSNMGLKREDSNKRGLQASTTPKSIASAANSPHHQM

QSNYSLGSPSSLSSSSASSPLGNVSNLVNIANNNTSGAGSGLVKPLQQKV

KLPPVGSPFPKPAYSYSCLIALALKNSRAGSLPVSEIYSFLCQHFPYFEN

APSGWKNSVRHNLSLNKCFEKIERPATNGNQRKGCRWAMNPDRINKMDEE

VQKWSRKDPAAIRGAMVYPQHLESLERGEMKHGSADSDVELDSQSEIEES

SDLEEHEFEDTMVDAMLVEEEDEEEDGDDDEQIINDFDAEDERHANGNQA

NNLPINHPLLGQKSNDFDIEVGDLYDAIDIEDDKESVRRIISNDQHIIEL

NPADLNATDGYNQQPALKRARVDINYAIGPAGELEQQYGQKVKVQQVIQP

QQHPPTYNRRKMPLVNRVI

>FBpp0071036_FoxN3_FoxN2/3

MSTDNPTQLLTTRDLSQLRGHLQSGTVAVPSTTTSVVAAAAATVAAAHGY

RTIAPPQQPANNSSTTTSATAASASASAQPPVIPPVISTSNSNMPASASK

YVFLQHNHNGGFTAYDGSAATTAAGSTGTVGATSGNIVLLAAAPLEASSG

EALVNVRTTEDDGELRSLSWLSDTNLIKGIVTTTAGQAGKRAATVAIKSA

TGAAGTVGSGAPNGNLCIVSDLIEELPTNLSETSEQESTGGAASGSAPAA

STVYSTTTVHKGASGTTTVVEYKASKANQQQQNSPQQQQQQQQQQQQQQQ

QQQQQQQQQQQQQQQQQTTYLPTPTRTTMVGHGYMPVVVSSASQPILSPA

KQQLPQQIYVTSNGSSPAGGGTIYKAASQALSSPTTSSSSSSNTTTHHPH

KKYLREKMHVQMDHSSHVASTVTVVSSPASSNNSSISSSNTGSVSGSGSS

TAAVNGGNSPVPKTSSFSTVFEAVNYATSAASNSSSSSNSTPNSQAILHG

QESPAVVTTTTLISAGTLPPGYSFVNQVASTPHVLSTPAHVASPETPPGA

ALLPGTYYATSTTSAGTTTTTLQARSLQLSVSPPPPNMTPTSQVHNGSLG

GGGGGGGGSGGGGGGGGANLRSPNSYASYDNEDSLKEFDLVVSSRLHTST

PQYSVSKNGANGYGNGNSNVNGSGAGSNTPQKQKHPNNVPYDPLVHTNNK

PPYSFSSLIFMAIEGSNEKALPVKEIYAWIVQHFPYFKTAPNGWKNSVRH

NLSLNKSFVKVEKAPNMGKGSLWRVEPQQRQNLIQALNRSPFFPNSAVDK

ISPSLKSPSGGSAYDSLDGGGSGSVSSAQPVAGGAGVPAAAAVALSTPTK

SNGLALANGASQATNAARPHSPNGGGSGSHARFDPYLFPNLSKAFRNIRE

DTVGQPMLQDALDDELSGDYHNNNNNNSGSKYNYMGANGSGGAGSGGVGS

NANGASDGINFARLARDCGADSIDDVHAAAAMLYLKHGPKIYSEPFQNGS

GPVITSSPSEDHTYSAGGNSNADSGSSTPLTNGNALASVAQAVAQGQNNQ

GGAGSDSNCASSDAAYDSSEENHNITPEEMADRQRHRDGVDALLSLSGSS

IVECGSVATTHYHSNGSSGSSHGSPHKRASSHSLEEEHLQQHREQQLQQQ

QQQQQHHHQQFGSSQAVYTNGSGSKMALLSSAAANVALAQQQQQQIHQDM

YSSTAGHNASMYLGGLNSHCLSGGGGVGVAAGMLPQHLSAATGASNKNKV

KPLRGLRTKFKRKSAWMR

>FBpp0293589_FoxO

MMDGYAQEWPRLTHTDNGLAMDQLGGDLPLDVGFEPQTRARSNTWPCPRP

ENFVEPTDELDSTKASNQQLAPGDSQQAIQNANAAKKNSSRRNAWGNLSY

ADLITHAIGSATDKRLTLSQIYEWMVQNVPYFKDKGDSNSSAGWKNSIRH

NLSLHNRFMRVQNEGTGKSSWWMLNPEAKPGKSVRRRAASMETSRYEKRR

GRAKKRVEALRQAGVVGLNDATPSPSSSVSEGLDHFPESPLHSGGGFQLS

PDFRQRASSNASSCGRLSPIRAQDLEPDWGFPVDYQNTTMTQAHAQALEE

LTGTMADELTLCNQQQQGFSAASGLPSQPPPPPYQPPQHQQAQQQQQQQS

PYALNGPASGYNTLQPQSQCLLHRSLNCSCMHNARDGLSPNSVTTTMSPA

YPNSEPSSDSLNTYSNVVLDGPADTAALMVQQQQQQQQQQQLSASLEDNN

CASTLIGQCLEVLNNEAQPIDEFNLENFPVGNLECNVEELLQQEMSYGGL

LDINIPLATVNTNLVNSSSGPLSISNISNLSNISSNSGSSLSLNQLQAQL

QQQQQQQQAQQQQQAQQQQQQHQQHQQQLLLNNNNNSSSSLELATQTATT

NLNARVQYSQPSVVTSPPSWVH

>FBpp0302969_FoxP1_FoxP

MHRIHDDEYSEDAKESDFKSSIQKEISVKSRHQISIPDICSDAVKNNCFP

PTGFLNNSITFASHVVKCSSPASSIDESSTAAQQHESNPHMHIQGQHMMA

PVPDLGFYNVPEFISEQEKLMFSDAERFLRSKDNEVCNNDFSYMHDEFAM

RKYYHPLFAHGICRWPGCEMDLEDITSFVKHLNTEHGLDDRSTAQARVQM

QVVSQLESHLQKERDRLQAMMHHLYLSKQLLSPTKIDRKDVPGREGKFCR

SPLTVNSIGRPIRQTNSPSPLNLPMVNSTNLCSIKKRNHDKNTFSINGGL

PYMLERAGLDVQQEIHRNREFYKNADVRPPFTYASLIRQAIIDSPDKQLT

LNEIYNWFQNTFCYFRRNAATWKNAVRHNLSLHKCFMRVENVKGAVWTVD

EIEFYKRRPQRTAGIGNNLTGATNSPDTNYFVAMNIGYVGLK

>FBpp0293409_FoxP1_FoxP

MHRIHDDEYSEDAKESDFKSSIQKEISVKSRHQISIPDICSDAVKNNCFP

PTGFLNNSITFASHVVKCSSPASSIDESSTAAQQHESNPHMHIQGQHMMA

PVPDLGFYNVPEFISEQEKLMFSDAERFLRSKDNEVCNNDFSYMHDEFAM

RKYYHPLFAHGICRWPGCEMDLEDITSFVKHLNTEHGLDDRSTAQARVQM

QVVSQLESHLQKERDRLQAMMHHLYLSKQLLSPTKIDRKDVPGREGKFCR

SPLTVNSIGRPIRQTNSPSPLNLPMVNSTNLCSIKKRNHDKNTFSINGGL

PYMLERAGLDVQQEIHRNREFYKNADVRPPFTYASLIRQAIIDSPDKQLT

LNEIYNWFQNTFCYFRRNAATWKNAIRTNLSLHKCFVRYEDDFGSFWMVD

DNEFVKRRHLSRGRPRKYEPSSSPNSCQSGNGVPTDKNPCDNCTQHCTSL

PPGADNPLDSNNPNDLGRIGCLPYCGSDGLSKASKDYSNMDSGMIESNSH

LTIDEYSTNMYESSANEHNR

>FBpp0070031_FoxG

MDTTPIFQSSFSIRSLLSVDKKEESPISKHNSGSSFSSCSSSSSNSSSDSMAAKSNAKPAFTYSALIVMAIWSSSEKRLT

LSGICKWIADNFPYYRTRKSVWQNSIRHNLSLNPFFVRVPRALDDPGRGHYWALDPYAEDLSIGETTGRLRRSNWQQNTG

ARPKVTGHPYQRMPYYGHGHGNGPYIKAHSAYFPIMDHQHHAAMVQHYQAMMHRYQMMPHPHHHQHQHQHQHPHSHFIQQ

SKPLHIQEPYHHTRYHLHQE

>DPOGS200192

MAIQNNPSRMLTLSEIYQFIMDLFPFYRQNQQRWQNSIRHSLSFNDCFVKVPRTPDKPGKGSFWTLHPDSGNMFENGCFLRRQKRFKDEKKESLRQAQKVAQGHGHHGGHDKREHSHEKSGGGAVPGEEKEMRDELLAQLHAAPELCLPEHTPLSLEHYAQLKQEPTGYAPAPHPFSITRLLPGADTKSDLKMYDVNYGYGHAPDQYYQSPLYHHHAHAHAQPPL

>DPOGS215651

MPRPSRESYGDQKPPYSYISLTAMAIWSSPERMLPLSEIYRFIMDRFPYYRRNTQRWQNSLRHNLSFNDCFVKVPRRPDRPGKGAYWTLHPQAFDMFENGSLLRRRKRFKLQKGEKDNLNAELAALASFNRAFLARQAGAPPPPPLPTAGLYTPPMCPQLSPEPAEIPEVASVTILPRERPRRAFTIDALLEPEPPRSSPSPPSPMLPIPVHMPRLELMASPYVVAAQRYQAELLQAVHALRPCYLPPPPLPVA

>DPOGS215763

MPRPTRESYGDQKPPFSYIALTAMAIWSSPERMLPLSEIYRFITDRFPYYRRNTQRWQNSLRHNLSFNDCFVKVPRRPDRPGKGAYWTLHPQAFDMFENGSLLRRRKRFKLHKGEKDSLNAELAALASFNRAFLARQAGTASGNMSSGIYAPSVTLCPRLSPETPEAPDTAALLPTLPRPRRAFTIDALLEPEPRRSSPSPPVVQPHCPMPLPPAPYLLAAQRYHAELLANLQQSCLPPLWTWRDTSQFSHYTLNS

>DPOGS215650

MRFTDASRYHITRRNERGRTRRGLYRPYPETTPSVWLYRSTTLPLHRSTAPANMPRPTRDSYGDQKPPYSYISLTAMAIWSSPERMLPLSEIYRFITERFPYYRRNTQRWQNSLRHNLSYNDCFVKMPRRPDRPGKGAYWTLHPQAFDMFENGSLLRRRKRFRLDKRQHDLREEFVEKQSRPTQRLDLDSCPNVAKPKKLFTIDSLLSNDEDKSSVKSAVREDRSSSFPQPELLALAANVRNDYHRRVQNGRAGMTEQRFSTVKLLVYE

>DPOGS203949

MHALFGEQSHYAYRSGAAGGYSAGVTPYAYDQYRYGYGPPYLHPHQQHVGTPKDMVKPPYSYIALIAMAIQNAPDRRITLNGIYQFIMERFPYYRENKQGWQNSIRHNLSLNECFVKVARDDKKPGKGSYWTLDPDSYNMFDNGSYLRRRRRFKKKDALKEKEEALKRQQQLQQAQELAAQEALSAADALGQARDVKPDVKPRIFECRPKREPGADCTRYDKLSEPIDEFSEPRLPPSAVYCSPQPYSLAAEEFRAATSGWYSAPEPSADQLPPAFRDLFEPPSCQLAGYRGSSPAPDAYRASPPPHHHYRSPAPSYYHHQACVAAAPASAHKSY

>DPOGS208014

MAILSSPDRKLVLSDIYQHILDNYPYFRSRGPGWRNSIRHNLSLNDCFVKAGRSANGKGHYWAIHPANIEDFRKGDFRRRKAQRKVRKHMGLAVDDDGEDSPSPPPQSPPPTALPIPFWGAGRLPGGVQPRKRQFDVASLLAPDDAPEKRARPDSSEEEIEEDIDVVASDQEENREEEPRIPLTPAAHYPLLGGWWPALDPALLQQLRRQAASPPSPNPPDHQRPPDT

>DPOGS214339

MAILQSPHKKLTLSGICEFIMTRFPYYREKFPAWQNSIRHNLSLNDCFIKIPREPGNPGKGNYWTLDPLAEDMFDNGSFLRRRKRYKRPAPSLQHAHAVVAMLAREAYAPLLPLPCYLPPSPLLSMPRPPPAALRPVPLPPRVPETTDTRGKRSRNGFSIESIIGSQENHVSEPRRSAFSPLHLGNGPPDDWA

>DPOGS203943

MKTEEQTTEEAARRAPATRRQEKPPYSYIALIVMAIRHSPNKRLTLSEIYAFLQQQFPFFRSSYQGWKNSVRHNLSLNECFVKLPKGLGRPGKGHYWTIDPSSEFMFEEGSFRRRPRGFRRKCQALKPQFGGGGYLCGTGAGALPPTQPANYELASGSGPSASTTTSVDYGACAYPHSSASQQLTYGGEYCTYGGMSDREWPLAYGPVESAYRPPPPSPTSRHDLPDLIPNYQYAVANDHVVEHPHDYYPTLSEPGHYLWLKYTSGQAI

>DPOGS202851

MSPPSFAIRDLLPEASSRSPSPSDTELDVTGTETPPPGCSTTDEKKNEKPAYSYNALIMMAIRSSPERRLTLNGIYEYIMKNFPYYKENKQGWQNSIRHNLSLNKCFVKVPRHYDDPGKGNYWMLDPSSDDVFIGGTTGKLRRRSTAASRSRLAAFKRGAVLHPMFGNPYASLVGLYPPLLSVYGRYALPSPLLRLPPPPPSPYHQLYRRIHGLSPPGSPPAEPESHLMKHTCLNYDKLGLQ

>DPOGS202854

MVKFEGDFSINAILMNHAVTKPPPSPTTSSTATPSEADLSDSELDVTGTGSEPVDCSKPRMEDDKKDKKHEKPAYSYNALIMMAIRNSPEKRLTLNGIYEYIMTNFPYYKENRQGWQNSIRHNLSLNKCFVKVPRHYDDPGKGNYWMLDASAEDVFIGGTTGKLRRRSALNGRSRLACFKRPLFPGAPLAGPYPPATYSQLVGLYSQLLYQRYAPMQMKTPPVTPGPVHPAFRNEMAYTSLPYSPLYGDRLPSGPFCQPLVHSPPTHSPPTSGSSSPELTSPSPSHHPLSPHIFKPVTVLTRQ

>DPOGS215040

MHITSSFFPASGEMLDASVNLYGDSGDQCGFFTLDEAVPESSHTVEIEYVYEQPDAGKNEELRTSKASSAQPKKTQAKQKQIKVVEEESETDLTNLTWLQNITNIMAMPQFPIPPMSPNPQVKVQPQNTRLQKFNQTIAKCQKDFMENKEEYQKNSDKKPPYSYSTLICMAMRYNNDKMTLSAIYSWIRDSFKYYRNADPTWQNSKVARSKHEPGKGGFWKLDLAHLEGTKRISNRPHKKKKNETKTEAKFDRKVSEEKTAVANIPQVDCVNMEDVGQAVTLHLPEFNLPLPDIEMTNVGANVIVEPVAPPLMPEDDLSSLLLSPTDWEDLQLDMLDNYLDSCFK

>DPOGS201179

MSDSPGVEWLPAYSPGPPRHSPLGPIPRFLYEDVQPPLLQQENNNKEIDQKQDKNKIKHAKPAYSYASMIRLAISSSPNGKMTLNEIYNYICNAFPYYKEAGKGWMMAPYSPECSSNSSGEAGASGVDAAPTPPATPTTPTAPTTPTTTTVLKEEPLVKGEPNLEHTDDALSALMDDELITDADISRESWWWSGARRHVCVVRHSALTDDYGNFLDLRHHCDCPPDTPDTADLAAQAPTTALTPPWHAL

>DPOGS209081

MWAARLCSAALVLDAKCTLRFPSTNIRLEFQSLVEESGVGSGGAGPPLPPLRISIPVDNDGRSPAPSPTGTISATNSCPTSPRGAGSSGRRHPDLGLVAQYAALADHQRPNSNGTAASSTSDSGYSSRDARDAREHREGRDEAKPPYSYAQLIVQAVASAADKQLTLSGIYSYITKHYPYYRTADKGWQNSIRHNLSLNRYFIKVPRSQEEPGKGSFWRIDPQSEGKLIELAFRPRRPRGVQFRAPFGLSSRSAPTSPSQVGVSGLVTPEELSREPTPDLFTAEEHEQQQSGQQRLSSSSQYLFPQRSGVSQSAPGSPGHGVYAGGSGLVMAGHQITVVTNGAGGEREEKYVVGTSGGGLVSIPEEEVQAANLLLHQHSPYYAGYSGDENCALGGELVIEEAPDDPPHKRPKHHVSDIEDRRAY

>DPOGS207171

MCLQESNTPDSFNQPEMKEAEDFSRVYQTLTLSALSRDDSANSPTSSENKPKPKPTPAACPGSSPEMNPQSTTPSSQALTKPPYSYVALIAMAITNSQNKRATLSEIYAYITKKFPFFEKDKKGWQNSIRHNLSLNECFIKVRREGGSESKGNYWTLDPQCGDMFVNGNFRRRRRMKRPFRAAPYKTMFDGYVAHGGQHPHMPIQLGHRNYFGSSTPYPPSYPRYDAWLSQPTGGLGYPAPIARSPPGCSPQASNVNPFSTHQNQGQLQSPLQSMQPMTMNYNTLNVAAIGEFDGSSSPGSGYAAGSFSPNRHHDIVTLSDAVSRFSFWPEGGSSSPNSGYVPTNFSPRRHEAVSSSDAAGRYSFWPDVGVKEESSSSLVANGGYTKYFM

>DPOGS204750

MASKTVSDKAKWRRRKFPKTVDEDNDCASLAWLLNFRLDEFVNVKVPDDEPVEALREVIEDSPPAAKKPPYTYPELIERALREKGELTVSAIYQWISDRFPFYKANDERWKNSVRHNLSINPHFRKGARASQGAGHLWSLAANAIDLLPLRNQTYEKTDVQETQIIECPKVIVLDEAAIAAASIMPDQDLFTGGTMFLNPVSTEQVVRECGLITGL

>DPOGS203265

MDLYITDSLQDMLDMDIKNEIATDLSSITDFSDSLGLNFSEMPPLLDMETDNSVTWLNNSSSFVHNLDLYGSEANAVMVNPNSVMPSTFAETPVKSIVKEEASHLLLTSAANNDLTNNTSLSSPKEEKSHLTFSPNAIKVAKVQESEDTKNKKPMEEATQMVIYVRKQDKTVVKDLLKDLDTNKTKSSTLTPTVRIKSSQQEVLKINNKNCSVLNTNQKLSQSLGTKTIISGNIHILDAQQSRTILANGNKQATILIDNSSLNNSRQIIKTSVTGAFTVDTSQAKYVNNSSKTVAGEFPKPAYSYSCLIAMALKNSRTGSLPVSEIYNFMCQHFPYFKTAPNGWKNSVRHNLSLNKCFEKIEKPSTNGSQRKGCLWAMNPSKVGKMDEEVQKWSRKDPQAIKKAMIYPETLEALERGEMKYSGFGSDNDADEDNDNDNDTEDLDLEIDPEESDQELEVEEVEGTGMVGAYRVLAPGLYGDLSDVEVLDQSYEEIDIDTKPVKLDLSVTENYTIHSAKRAKTSFIYQPVTSQTHTSRRKTPLVNRIALV

>DPOGS202627

MAPMRGERGGSPARGARASLPLPRRLIRQIARHVASSGRSLRRLEVSGRVIDNFDDIDDGSDESDMETQTAVACAGNSGNLPPADHDDDLTSLSWLQDKNLLSGINLTKSDIEDSKLISSQVVVKMAPMRGERGGSPARGARASLPLPRRLIRQIARHVASSGRSLRRLEVSGRVIDNFDDIDDGSDESDMETQTAVACAGNSGNLPPADHDDDLTSLSWLQDKNLLSGINLTKSDIEDSKLISSQVVVKTEPSITPPPSSRVPSPPRTPCKAPPSPPAVNTHTKPPYSFSCLIFMAIEAAPARALPVKEIYAWIVRHFPYFKHAPQGWKNSVRHNLSLNKCFHKVAAAPGLGKGSLWTVDPQHRSSLLQAFGRQPVPPVEVEEQETSTSVKNTPDPQLFPYLARRLADARSPPGADEYLAAATVLAMKYGPAVLDQLPPEAHLVISRCARDEHSYSGGEERRTAEALLNLAGVRPHAPS

>DPOGS208862

MSLQRGIYQSPWSSQTALSELEGMGELEPLGELGEVGFEPQTRARSNTWPLPRPENYIEPDDGGSKKNSNQNLTGAPPLPSTSGTTKKNSSRRNAWGNLSYADLITQAITSSQDNRLTLSQIYEWMVQNVPYFKDKGDSNSSAGWKNRWLLFNVCAQVNNDFRVPFCQSINTVDI

>DPOGS213641

MRKIEQKLEESQLPEEDRMAGDAVWGTIGGPEPALNSSPGGAAKPPPLNSFELDRCLDREESGRWGEAARRRRRSSPRRSPPPGNEPLSLARASAPASPLVTSSPSSPSPARSPLLAPDLLAMQLLDQHSQLQALMKQRLFHQHHLQKQHMSSEAAKRQLEQSRLQDQINLNLLSQSHLQPPETSPSLQQQQLVQQLQAVQRQYLMHAPMSVPPNAPPDYDTGSELEEHPLFGRGVCKWPGCDALAEDFQAFLKHLEAAHTLDDRSAAQARVQMQVVAQLELQLRRERDRLAAMMRHLHAARDNHNKMHVVISMSVSPGPASEGSSPGPVRRRVSDKSGVAIAGGLPYMLERAGLDVQQEIQRNREFYKTADVRPPFTYASLIRQAIIESPDKQLTLNEIYNWFQSTFCYFRRNAATWKNAVRHNLSLHKCFMRVENVKGAVWTVDEVEFYKRRPQRAHAAIHTGYCS
